# Detailed molecular and epigenetic characterization of the pig IPEC-J2 and chicken SL-29 cell lines

**DOI:** 10.1016/j.isci.2023.106252

**Published:** 2023-02-20

**Authors:** Jani de Vos, Richard P.M.A. Crooijmans, Martijn F.L. Derks, Susan L. Kloet, Bert Dibbits, Martien A.M. Groenen, Ole Madsen

**Affiliations:** 1 Animal Breeding and Genomics, Wageningen University & Research, Wageningen 6708PB, the Netherlands; 2 Human Genetics, Leids Universitair Medisch Centrum, Leiden 2333ZC, the Netherlands

**Keywords:** Cell biology

## Abstract

The pig IPEC-J2 and chicken SL-29 cell lines are of interest because of their untransformed nature and wide use in functional studies. Molecular characterization of these cell lines is important to gain insight into possible molecular aberrations. The aim of this paper is to provide a molecular and epigenetic characterization of the IPEC-J2 and SL-29 cell lines, a cell-line reference for the FAANG community, and future biomedical research. Whole genome sequencing, gene expression, DNA methylation, chromatin accessibility, and ChIP-seq of four histone marks (H3K4me1, H3K4me3, H3K27ac, H3K27me3) and an insulator (CTCF) are used to achieve these aims. Heteroploidy (aneuploidy) of various chromosomes was observed from whole genome sequencing analysis in both cell lines. Furthermore, higher gene expression for genes located on chromosomes with aneuploidy in comparison to diploid chromosomes was observed. Regulatory complexity of gene expression, DNA methylation, and chromatin accessibility was investigated through an integrative approach.

## Highlights

• Untransformed, nontumorigenic IPECJ2 and SL-29 cell lines show some aneuploidy
• Aneuploid chromosomes result in increased gene expression
• Positive correlation between hypomethylation, H3K4me1, H3K27ac, and gene expression
• Identified enhancer and promoter regions play an important role as a reference

## Graphical abstract

**Figure:**
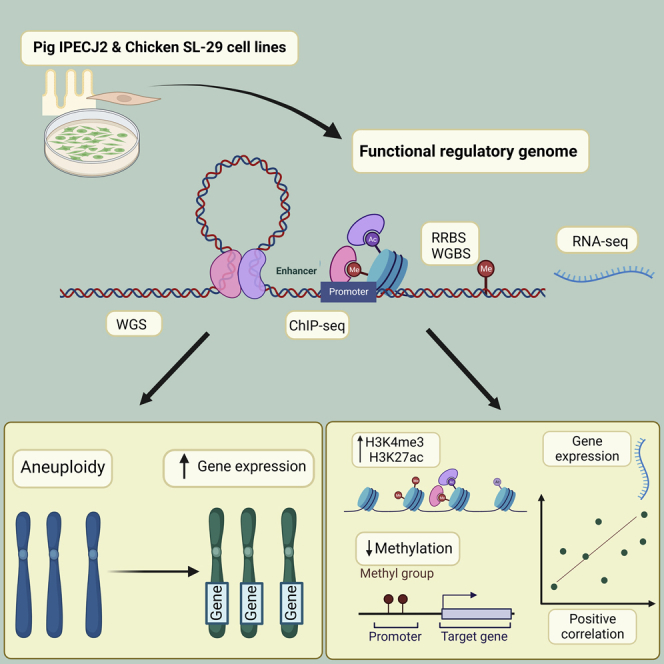

## Introduction

The genome of all eukaryotic species is regulated at the chromosome level,^1^ where DNA is packaged in a highly organized structure of DNA and histones. Gene expression is regulated through a network of physical interactions of enhancers, promoters, insulators, epigenetic marks, and chromatin-binding factors, which is responsible for the chromatin accessibility. Epigenetic marks such as DNA methylation, noncoding RNAs, and histone modifications can be investigated to obtain insight into regulation of the epigenome. Some histone modifications are highly informative regarding gene expression and are associated with transcriptional activation, promoters, and enhancers.^2^^,^^3^^,^^4^^,^^5^ In addition, DNA methylation is important in identifying gene expression and gene silencing, as methylation and gene expression generally show an inverse correlation. Together these (epi)genetic marks can be used to annotate the functional genomic elements that determine gene expression.

The Functional Annotation of Animal Genomes (FAANG) consortium is a scientific driven community, with the aim of providing the functional annotation (functional maps) specifically for farm and companion animals.^6^ Earlier projects in human^7^ and model animals (e.g. Mouse ENCODE) provided strategies for using omics data to obtain insights into the functional genome; this is achieved by performing genome-wide analysis focusing on genome expression, regulatory functions, methylation, chromatin accessibility, and modifications, providing insights into the functional genome.

Cell lines provide an interesting model to study the genomic architecture and regulatory genome of species of interest. Cell lines directly derived from tissues or organs of an animal are referred to as primary cell lines. Such cells can then either continue growing indefinitely or die off after a certain number of cell divisions.^8^^,^^9^^,^^10^ Cell lines that can be grown indefinitely (i.e. that have become immortalized) often show cell aneuploidy or heteroploidy, which is most pronounced in cancer cell lines.^10^^,^^11^ In this study, a pig IPEC-J2 and chicken SL-29 (CRL) cell lines were used. The pig IPEC-J2 cell line is frequently used in e.g. intestinal transport studies due to the uniqueness of the cell line being neither transformed nor tumorigenic in nature. Chicken SL-29 (CRL) is useful for investigation of the substrate of virus propagation, recombinant protein expression, and recombinant virus production.^12^ Characterizing commonly used cell lines holds value for the FAANG community, where further comparative and/or combined studies will be performed. Determination of technical variation in data between different labs is important to identify, as it will be useful in future comparative analyses in identifying differences due to biological variation. The main aim of the current research was the molecular characterization of the pig IPEC-J2 and chicken SL-29 cell lines using an integrative approach of a variety of omics data (genome sequencing, epigenomic modifications, DNA methylation, and RNA-seq).

## Results

The IPEC-J2 cell line in pigs and the chicken SL-29 cell line are of interest for the animal genomics community because of the untransformed nature and wide use in functional studies in these cells. We have analyzed both the pig and the chicken cell lines with a range of whole genome-based assays. We first report the results from the pig IPEC-J2 cell line followed by the chicken SL-29 cell line. For each of these cell lines, we first focus on chromosome level structural variation followed by an in-depth analysis of the expression, chromatin accessibility, and methylation of the cell line genomes.

### Pig IPEC-J2 cell line

#### Chromosomal abnormalities and variations within the genome

**Figure 1:**
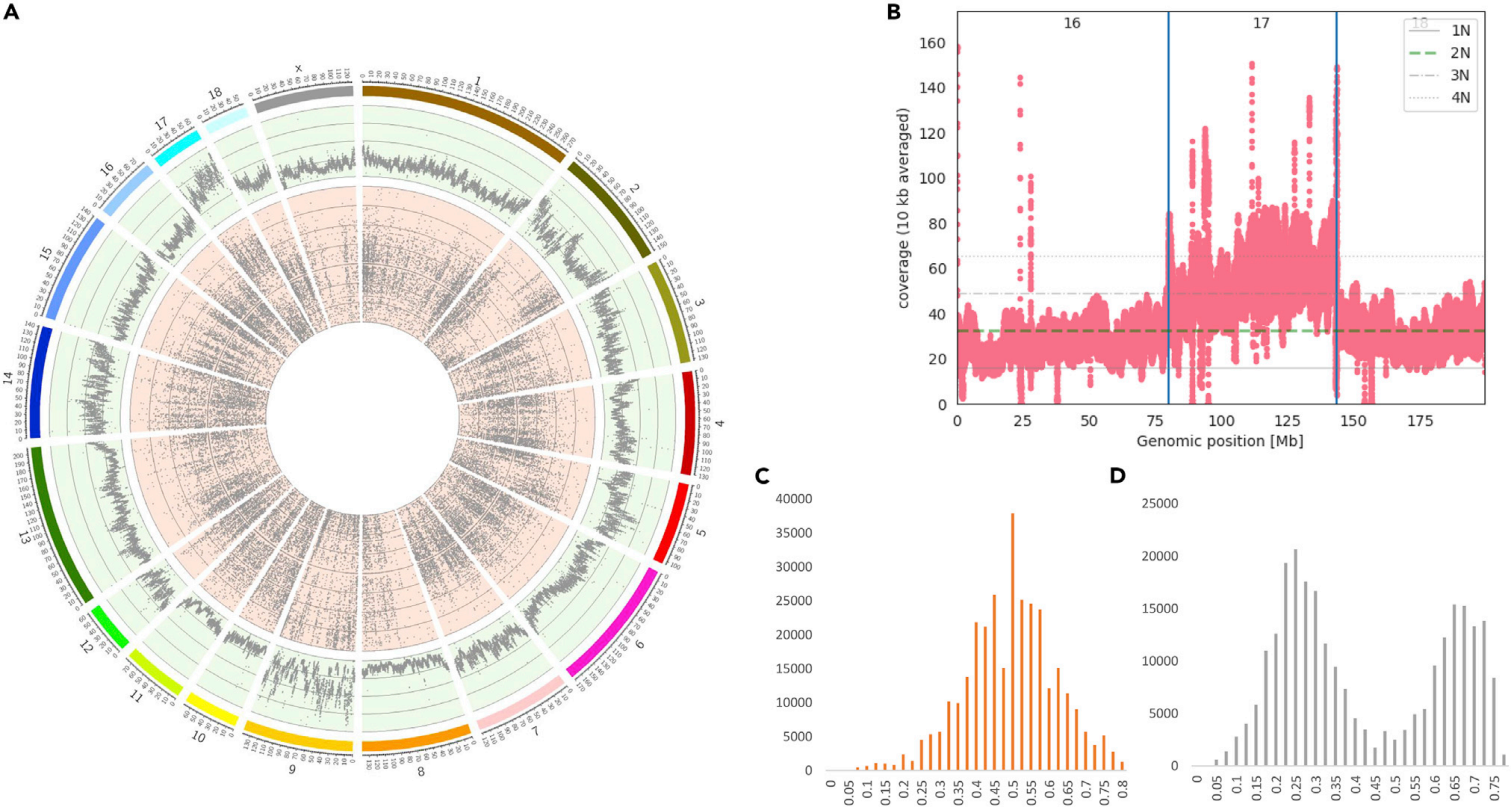
Chromosomal abnormalities in pig IPEC-J2 For a Figure360 author presentation of this figure, see https://doi.org/10.1016/j.isci.2023.106252. (A) Circos plot showing the read-depth per chromosome in bins of 50 kb on the genome-wide level (outer track shown in green) and the allele support for heterozygous SNPs called from the WGS data per chromosome (inside track shown in red). The scale of the read-depth track starts from a minimum of 10 and increases in counts of 20 per line up to a maximum of 90. From the SNP distribution on the inner track of the plot a normal diploid chromosome will result in many heterozygous SNPs where both alleles are supported by ∼50% of the reads. However, a triploid chromosome would result in heterozygous SNPs supported by ∼33% or 67% of the reads. (B) Representation of WGS read-depth for chromosomes 16, 17, and 18, indicating triploidy of chromosome 17. (C and D) Histogram of the (ratio of the allele) count of reference allele for heterozygous SNPs of chromosome 16 (C) and chromosome 17 (D).

The whole genome sequence data were investigated in different ways to determine the structure of the genome. Chromosomal abnormalities such as aneuploidy and heteroploidy can occur in cell lines that grow indefinitely. We therefore first investigated the chromosomal structures and possible changes in aneuploidy. Aneuploidy events can be detected using whole genome sequencing (WGS) data by examining both the read-depth and the ratio of reads that support the alternative allele for heterozygous SNPs; this should be around 0.5 for diploid chromosomes (Figure 1D), whereas e.g. around 0.33 or 0.67 is expected for triploid chromosomes. Figure 1A provides an overview of the read-depth and ratio of read support for heterozygous SNPs called from WGS data.

Some chromosomes, e.g. chromosome 17 (Figure 1D), show clear evidence of aneuploidy, whereas other chromosomes contain large structural variations such as chromosome 2. This chromosome shows a diploid allele ratio distribution of 0.5 for the first part of the chromosome and a distribution of the SNPs around 0.75 and 0.25 toward the end of this chromosome, indicating possible triploidy for this segment of the chromosome. This possible triploidy is less clear from the read-depth for this part of this chromosome. A low number of heterozygous SNPs is observed for the first half of chromosome 8 (which could indicate partial monosomy), whereas the second half of chromosome 8 has SNP support ratios at around 0.25 and 0.75, supporting higher ploidy. However, this observation on chromosome 8 is not very well reflected in the read-depth for this chromosome. On chromosome 9 and 17, the read-depth is significantly higher compared with other chromosomes, indicating higher ploidy levels of these chromosomes; this is supported by the allele ratio distributions of these two chromosomes, where most SNPs are observed at a frequency of 0.25–0.35 and 0.65–0.75, indicating possible triploidy. Much variation in read-depth is observed for the individual chromosomes (Figure S3), specifically for chromosome 16 (Figure 1B), showing a likely deletion between position 9 and 17 Mb.

**Figure 2:**
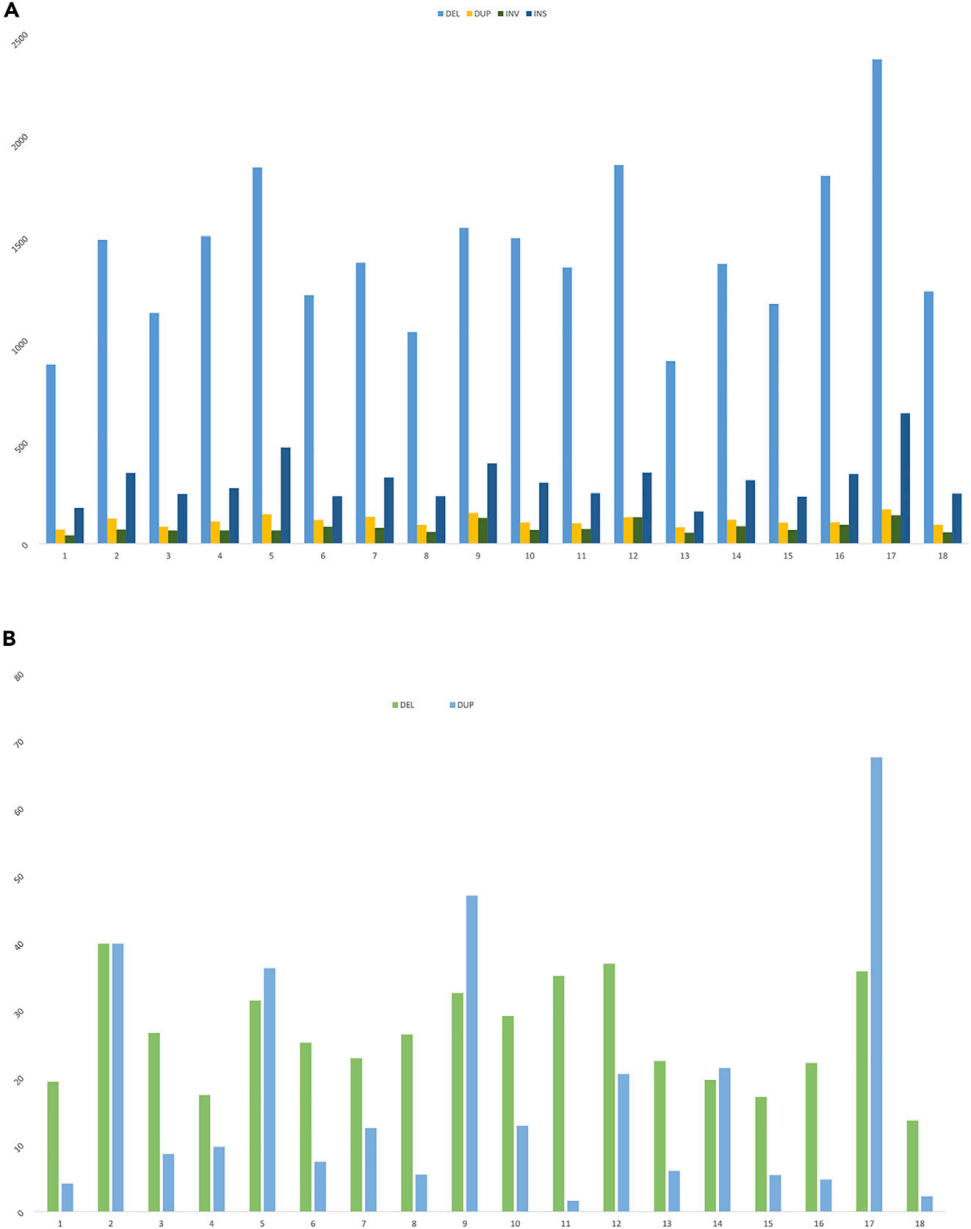
Structural variations observed in pig IPEC-J2 cell line Relative number of SVs per chromosome. Data normalized for size of chromosomes, with normalized counts shown on the y axis, detected by (A). Manta and (B) CNVnator. DEL, deletion; DUP, duplication; INV, inversion; and INS, insertion.

We further investigated additional structural variants within the genome of this cell line with Manta (Figure 2A) for the detection of small variants and CNVnator for large variants (>1 Mbp) (Figure 2B).

For small variants, deletions are the most abundant type of variant for all chromosomes, and relatively more insertions and deletions are observed on chromosome 17. For large variants, the number of deletions and duplications vary between the chromosomes, and the chromosomes showing evidence of triploidy (Figure 1) have a relatively large number of duplications. We further investigated the effects of the structural variation, i.e. variants potentially affecting genes or regulatory regions using the VEP tool. As expected, most structural variants (small and large variants) are found within intron and intergenic regions. The most prominent effect was observed for large variants, with 25% affecting transcription amplification and 17% transcription ablation. Results for both large structural variation (SV) and copy-number variation (CNV) variant effect prediction analyses are shown in the Figures S4 and S5.

#### Gene expression profile

RNA-seq data provide insight into gene expression levels across the genome. These data can provide insight into elements that regulate gene expression like promotors, enhancers, and epigenetic marks, as well as chromosomal abnormalities affecting them. Of the 31,907 genes tested, 10,412 were expressed (transcript per million [TPM] >1).

**Figure 3:**
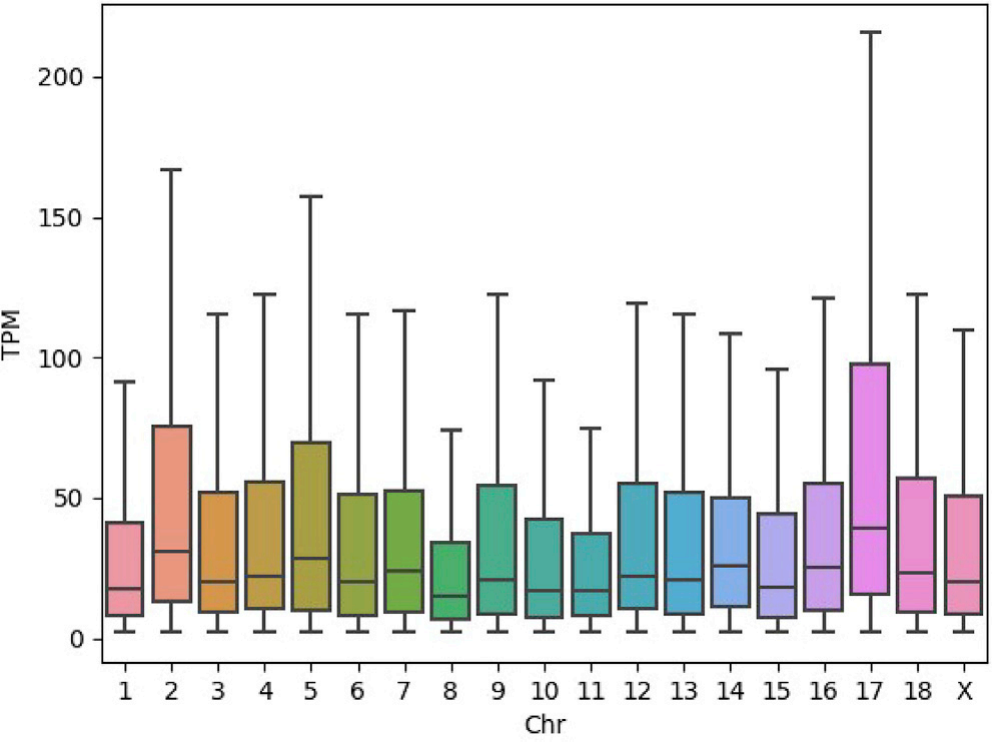
Gene expression profile of the pig IPEC-J2 cell line Boxplots of the TPM expression values of genes per chromosome. Only genes with TPM>2 were used, which removes genes with very low/no expression.

Interestingly, the expression levels on chromosomes 2, 5, and 17 (all chromosomes with ploidy aberration or large structural changes, Figure 3) were higher compared with the diploid chromosomes. Gene expression levels per chromosome in the IPEC-J2 cell line were compared with the gene expression levels for jejunum tissue and jejunum-derived organoids and IPEC-J2 samples cultured for a longer time (van der Hee et al.^13^; related to Figure S6). The gene expression in IPEC-J2 cells seems to be more variable between chromosomes when compared with gene expression in jejunum tissue and the organoids derived of the jejunum tissue (Table S3, supplemental text S1). A clear elevated gene expression is detected on chromosome 17 in the IPEC-J2 cells compared with tissue and organoids, and a notably high gene expression is observed on chromosome 2 for all samples.

#### Chromatin accessibility and genome architecture

Genome-wide profiling of histone modifications provides insight into chromatin structure, as well as the location of regulatory elements such as promoter and enhancer regions. Therefore, ChIP-seq data were generated and analyzed for four different histone modifications (Table 1) and the insulator CTCF to provide insight into the regulatory genome of the IPEC-J2 cell line. Furthermore, to assess the conformity between ChIP-seq experiments, ChIP-seq for three marks was repeated in another laboratory. The results of peak calling from the two respective experiments are shown in Table 1.

**Table 1:** Number of ChIP-seq reads, peaks identified per mark, and overlap between rounds of experiments

Histone modification	Round1 # Reads	Round2 # Reads	Read coverage correlation of overlapping regions	# Peaks Round1	# Peaks Round2	Overlap	Signal value correlation of overlapping peaks
H3K4me1^b^	27080156	32321068	0.52	99735	59260	54607	0.1
H3K4me3^a^	18423318	28552134	0.9	22074	24448	16759	0.62
H3K27ac^a^	25285609	20466802	0.8	52584	48000	45199	0.3
H3K27me3^b^	26873342	na	na	46470	na	na	na
CTCF^a^	34767672	na	na	7555	na	na	na

na, not analyzed.

a Narrow peak.

b Broad peak.

A high number of peaks were shared between the two experiments (70%–95%), with histone mark H3K4me3 sharing the lowest number of peaks most likely resulting from the lower number of reads for this mark in the first experiment. We assessed the relationship of the shared peaks and investigated the similarities between the two experiments, by calculating Pearson correlations (Table 1). A high correlation is seen for the read coverage of overlapping peak regions for H3K4me3 and H3K27ac. Correlations between the signal values (measure of overall enrichment of the region) of overlapping peak regions for each experiment are low for H3K4me1 and H3K27ac and moderate for H3K4me3. Histone modifications enriched around the transcription start site (TSS) (+/− 1000 bp) are generally indicative of promoter activity. Histone marks H3K4me3 and H3K27ac are enriched around the TSS of expressed genes (TPM > 1) (Figure S7, supplemental text S2), with histone mark H3K4me3 enriched at approximately ∼17.5% of the TSS and histone mark H3K27ac enriched at ∼5% of the TSS.

**Figure 4:**
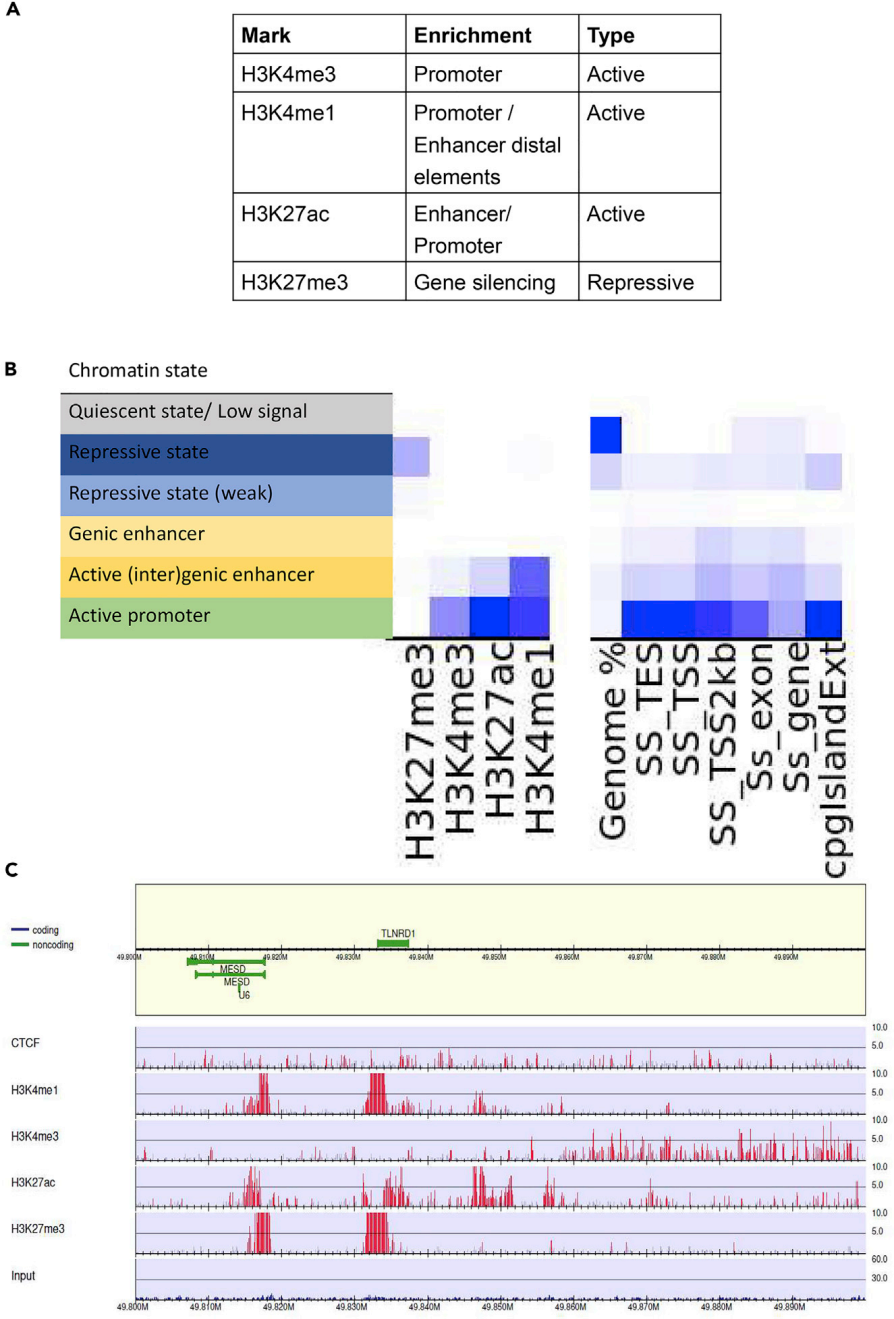
Histone modifications in pig IPEC-J2 cell line (A) Summary of the informativity of the histone marks. (B) Six chromatin states were defined using 4 histone modifications (H3K4me1, H3K27me3, H3K4me3, and H3K27ac), with the left panel describing the chromatin state annotations, central panel showing the emission coefficients in the ChromHMM model, and the right panel showing the relative enrichment of coverage for the whole genome (genome%), transcription start site (SS_TSS), transcription end site (SS_TES), 2000 bp surrounding the TSS (SS_TSS2kb), exon regions (Ss_exon), genic regions (Ss_gene), and CpG islands. (C) Individual histone modification and CTCF profiles in the IPEC-J2 cell line for the *MESD* (TPM = 110.85) and *TLNRD1* (TPM = 42.63) genes (both involved in mesodermal development) on chromosome 7.

In ChromHMM analyses, the active promoter state is identified by an enrichment of histone marks H3K4me3, H3K27ac, and H3K4me1 (Figure 4). The two enhancer states show an enrichment in H3K4me1 and H3K27ac. The histone mark H3K27me3 indicates the gene silencing state. Most of the genome is in a quiescent state (without any of the assayed histone marks; Figure 4B right panel), whereas a small fraction is in the weakly repressive state. Both the TSS as well as TSS +/− 2 kb show enrichment in promoter and enhancer states (Figure 4B, right panel). The CpG islands show strong enrichment for the repressive state as well as the promoter state. The TSS is highly enriched for the active promoter and enhancer states. Figure 4C shows a typical example of the genome distribution of the histone marks. A strong enrichment of H3K27me3, H3K4me1, and H3K27ac can be seen around the *MESD* and *TLNRD1* TSS (see Figure S8 for read alignments in this region). Further downstream of these genes, enrichment of the H3K4me3 mark can be observed in a gene-poor region (gene desert). Annotation of the peak regions identified for each histone modification provides insight into the types of functional elements close to the histone modifications (Figure S9). The annotation of these peak regions shows distribution of histone modification peaks in different genomic regions, which confirm the enrichment of H3K4me3 and H3K27ac around the promoter region.

Overrepresented sequence motif analysis for histone marks associated with promoter and enhancer regions identifies types of transcriptions factors (TF) related to gene expression. The peaks called for H3K4me3, H3K27ac, and H3K4me1 were provided to Homer for identification of enriched TF involved in gene expression in IPEC-J2. Table 2 shows the three most enriched motifs and their corresponding TF identified for three histone marks associated with promoter and enhancer regions. Other motifs enriched (motifs with p values <1e-12) are shown in supplementary results (Data S1)

**Table 2:** Motif enrichment in histone H3K4me3, H3K27ac, and H3K4me1 peak regions

Histone mark	Motif	Transcription factor	% of target regions	p-value
H3K4me3		PB0075.1_Sp100_1	54.95	1e-316
		IRF2(IRF)	4.45.	1e-287
		SD0001.1_at_AC_acceptor	68.6	1e-220
H3K27ac		E2F	49.69	1e-141
		ZNF449	45.56%	1e-120
		REL	41.29%	1e-111
H3K4me1		Fos(bZIP)	8.24%	1e-305
		TEAD(TEA)	20.48%	1e-188
		HOXC13	50.85%	1e-120

Top three enriched known binding motifs identified from consensus peaks. Motifs are shown in color in the supplemental files.

The significant transcription factors identified were Sp100 and IRF for the peak regions of H3K4me3, E2F and ZNF449 for H3K27ac, and FOS and TEAD for H3K4me1. We identified 36,638 enhancer regions, and motif analysis for these enhancers is shown in Data S1. Significant TFs identified within the enhancer regions include Fra1, TEAD3, EWS-ERG fusion, and FOXN3, which are responsible for cell growth, tumor suppression, and suppression of transcription of transforming growth factor and play a role in several cancers. The CTCF motif generated using both MEME and homer tools (Figure S10) shows high similarity with the human consensus sequence supporting the conserved nature of CTCF-binding sites.

#### DNA methylation profile of the genome

Gene expression is negatively correlated with DNA methylation; therefore, investigating the methylome provides further insight into specific characteristics of the cell line’s genome characteristics. The methylome was investigated using both reduced representation bisulfite sequencing (RRBS) and whole genome bisulfite sequencing (WGBS) (Table 3).

**Table 3:** Average methylation levels for cytosine in different base content between RRBS and WGBS for pig IPEC-J2 cell line

Site	Assay	Average methylation level (%)
CpG	RRBS	42.76
CHG		0.6
CHH		0.53
CpG	WGBS	45.6
CHG		0.35
CHH		0.33

Most DNA methylation is observed at CpG sites (42.76%–45.6%, Table 3), whereas at non-CpG sites little DNA methylation is detected (0.68%–1.13%). Therefore, as expected, the cell line displays a methylation pattern similar to what has been observed in porcine tissue methylation studies.^14^ The average coverage for the RRBS methylation of chromosomes is 14.2, and most chromosomes are similarly covered (Figure S11), except for chromosome 17, which has a higher read coverage of 22.4, confirming the triploid status of this chromosome also supported by the RRBS data. A global view of WGBS CpG methylation levels per chromosome shows methylation levels to fluctuate between 0.3 and 0.6 (Figure S12).

WGBS is referred to as the gold standard for investigating DNA methylation, in particular because it provides more information on a whole genome level compared with RRBS. RRBS is usually perceived as being more cost-effective, as a reduced number of sites are sequenced (usually more focus on CpG regions). We therefore investigated whether all sites identified by RRBS are also covered by WGBS. To do so, we disregarded any sites with a coverage <10 for both types of data as not being sufficiently informative. In total, 23,952 sites covered by RRBS are not covered by WGBS. Further details on possible functional relevance of these RRBS-specific sites is done for the chicken cell line (see later discussion).

#### Integrative insight into epigenome marks

**Figure 5:**
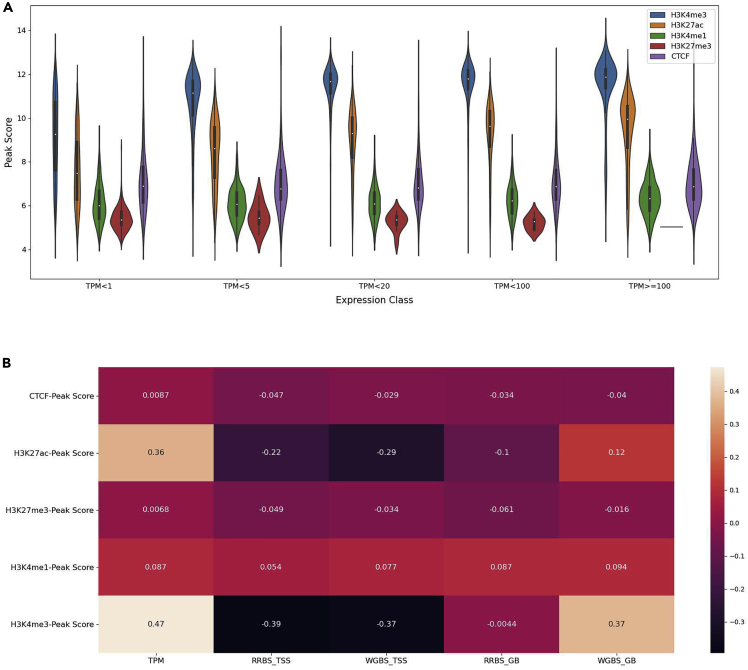
Integrative insight into histone marks and DNA methylation together with expression in pig IPEC-J2 Integrative analysis of various histone marks and DNA methylation together with gene expression. (A) Violin plots of the peak score for each histone mark relative to the TPM expression values. TPM values are divided into different classes ranging from very lowly expressed (TPM>1) to very high expression values (TPM>100). (B) Heatmap of the peak score values for each histone mark relative to the TPM expression values as well as methylation levels (RRBS and WGBS) at different positions. RRBS_TSS = RRBS level at the transcription start site (TSS), RRBS_GB = RRBS level within the gene-body (GB), and WGBS level at the same locations TSS and GB. Levels of correlations are shown by color panel on the right, and the value of each correlation is also shown on the heatmap.

The interactions between regulatory elements and methylation play a critical role in gene expression. We studied these complex interactions and the relation to gene expression using an integrative approach. The individual relationships between the regulation of gene expression and histone modifications (H3K4me1, H3K4me3, H3K27ac, H3K27me3) and CTCF are shown in Figure 5A.

The histone modifications associated with promoter and enhancer regions (H3K4me3, H3K4me1, and H3K27ac) show patterns as expected with a positive correlation with gene expression. Histone mark H3K27me3, which is associated with gene suppression, shows a negative correlation with gene expression (Figure 5B). As expected, there is a negative correlation for the methylation data at the TSS with the promoter and enhancer regions (H3K4me3 and H3K27ac). The methylation levels within the gene-body (GB) show both a weaker negative correlation to CTCF and H3K27me3 and a positive correlation (WGBS_GB) with H3K4me3 and H3K27ac. A positive correlation is also observed between gene expression and the histone marks H3K4me3 and H3K27ac.

**Figure 6:**
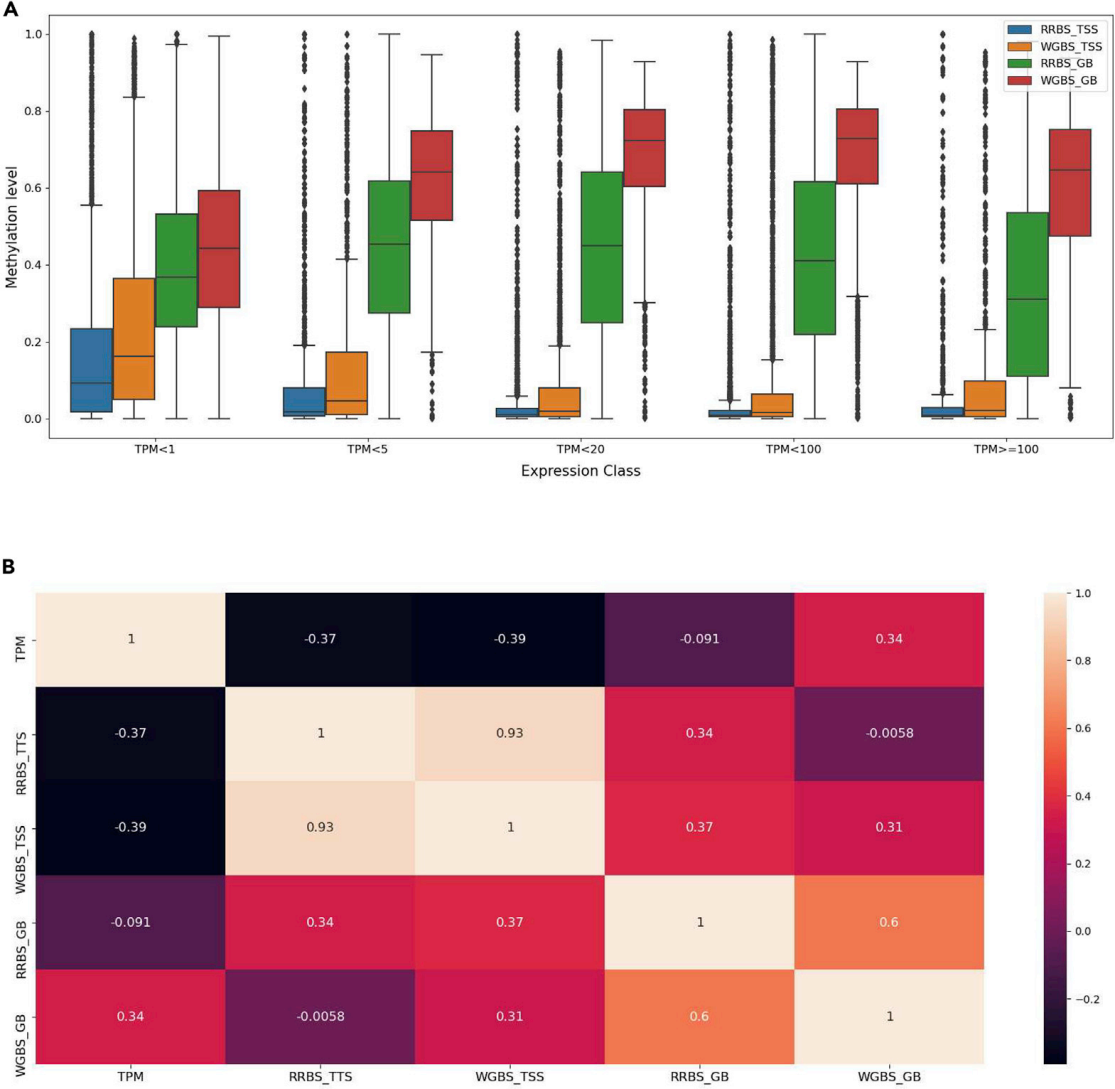
Integrative insight into DNA methylation together with expression in pigIPEC-J2 Box showing integrative analysis plots for methylation levels and expression levels. (A) Average methylation level at various regions (RRBS and WGBS data at the TSS and GB, notations are as above in Figure 11) for different levels of expression. (B) Heatmap of the various groups of methylation levels (RRBS and WGBS at the TSS and GB) together with expression.

The methylation levels at the TSS and within the gene-body for RRBS and WGBS (Figure 6A) follow the expectation that methylation levels are negatively correlated with gene expression (i.e. highly expressed genes show lower methylation levels and vice versa) (Figure 6B). This relationship between methylation and expression values is especially evident at the TSS. Low methylation of RRBS within the gene-body can likely be explained by a lack of coverage in the gene-body compared with WGBS. In addition, for WGBS the methylation level in the gene-body increases at higher gene expression.

### Chicken SL-29 cell line

#### Chromosomal abnormalities and variants in the genome

**Figure 7:**
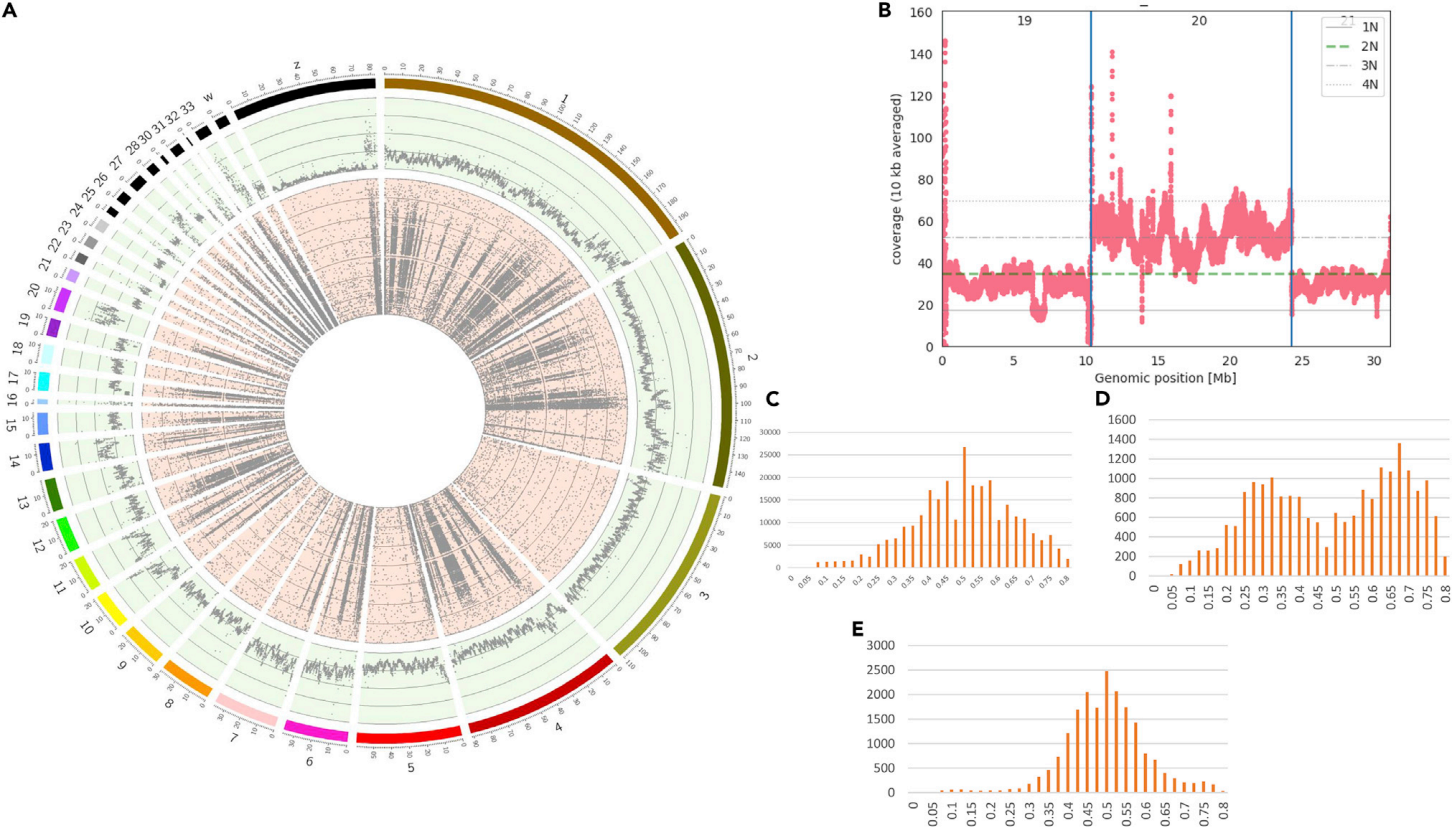
Chromosomal abnormalities in chicken SL-29 (A) Circos plot showing the read-depth per chromosome in bins of 50 kb on the genome-wide level (outer track shown in green) and the inside track (in red) showing the allele support for heterozygous SNPs called from the WGS data per chromosome. (B) Read-depth for individual chromosomes 19, 20, and 21, which show the higher read-depth in a tetraploid chromosome (chromosome 20) compared with chromosomes 19 and 21. (C–E) Examples of SNP distribution of a diploid. (C) Chromosome 1: it can be observed that many heterozygous SNPs are supported by ∼50% of the reads. (D) Chromosome 11: it would result in heterozygous SNPs supported by ∼33% or 67% of the reads. (E) Chromosome 20: many heterozygous SNPs are supported by ∼50% of the reads.

As with the pig cell line, WGS for the chicken cell line provided a comprehensive insight into genetic variation, chromosomal abnormalities, and structural variation at the global genome level. Multiple chromosomal abnormalities such as aneuploidy and heteroploidy are observed for the chicken SL-29 cell line (Figure 7).

**Figure 8:**
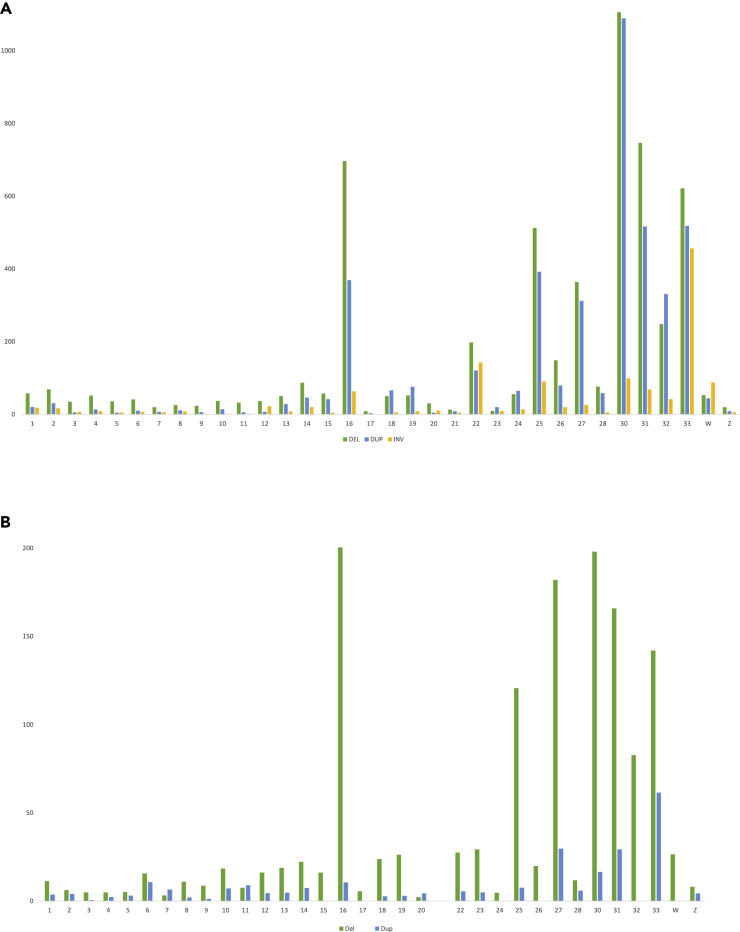
Structural variations observed in chicken SL-29 cell line Normalized count of SVs per chromosome, with normalized counts on the y axis. (A) Manta for copy-number variants and (B) CNVnator for structural variants >1 kb.

A higher read-depth (Figure 7A, first outer line of the read-depth track) is seen for chromosomes 6, 7, 11, 14, 20, 27, and 33 in comparison with other chromosomes in the genome. Figures 7C–7E show the SNP distribution for chromosomes 1, 11, and 20, with chromosome 20 classified as a possible tetraploid. The region at 7–8 Mb on chromosome 19 shows low read-depth (Figure 7B), suggesting a possible large deletion at that position within this cell line. We assessed the presence of structural variants, in particular deletions and duplications, using Manta. Results show different small structural variants within the genome of this cell line (Figure 8A). Several large copy-number variants such as deletions and duplications of more than 1Kbp were detected using CNVnator, (Figure 8B).

Relative abundance of copy-number variants is higher for some of the small micro-chromosomes (i.e. chromosomes 16, 25, 30, 31, 32, 33), whereas the macro-chromosomes have relatively fewer copy-number variants. Intron variants (48% of total variants) and intergenic variants (28% of total variants) are the most abundant consequences from the copy-number variants in this cell line. The effects, as determined by VEP, of both copy-number variants and large structural variants identified through CNVnator are shown in Figures S13 and S14.

#### Expression profile of the genome

The expression profile for this cell line was investigated to obtain further insight into the genes expressed and interaction of regulatory elements, aneuploidy, and CNVs affecting gene expression. We tested the expression of 24,356 genes, of which 13,546 were expressed (TPM > 1).

**Figure 9:**
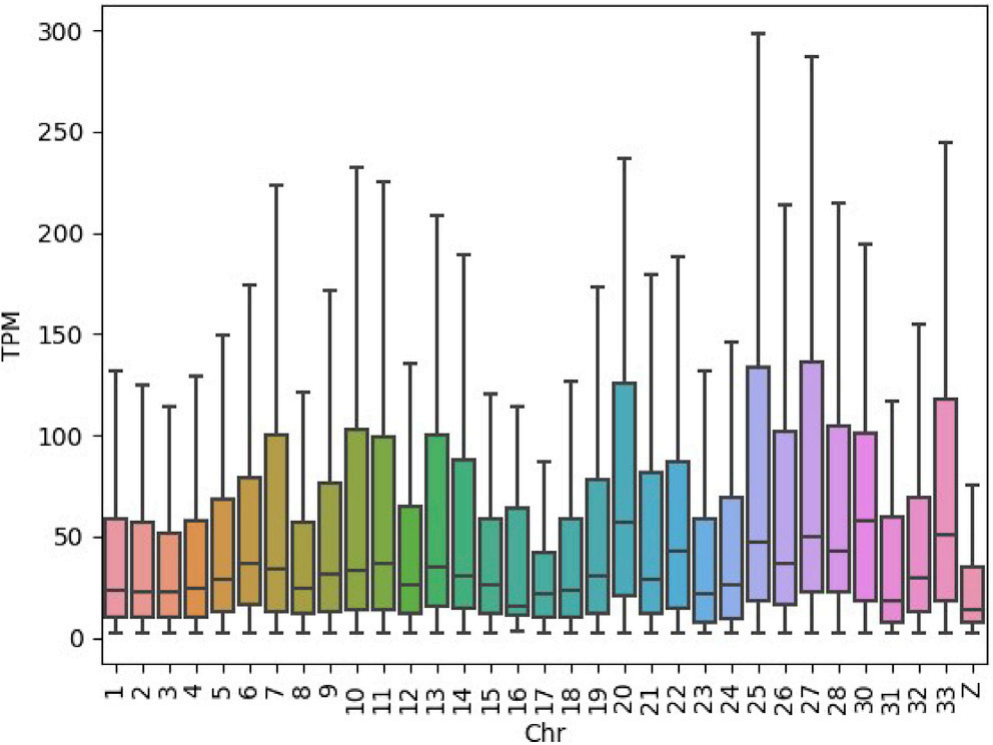
Boxplot of the expression levels of genes per chromosome

Higher levels of gene expression are observed on chromosomes 20, 25, 27, and 33 (Figure 9, supplemental text S4). This shows the influence of aneuploidy and structural variation on gene expression levels, e.g. with tetraploid chromosome 20 showing a higher expression level compared with the diploid chromosomes (e.g. chr 1).

#### Chromatin accessibility and genome architecture

As described for pigs, histone modifications are of importance to investigate chromatin accessibility and provide further insight into regulatory elements. The peak calling results for the chicken cell line are shown in Table 4.

**Table 4:** Number of peaks identified per mark for the respective rounds of the experiments, overlap, and number of reads for each round of experiment

Histone modification	Round1 # Reads	Round2 # Reads	Read coverage correlation of overlapping regions	# Peaks Round1	# Peaks Round2	Overlap	Signal value correlation of overlapping peaks
H3K4me1^b^	36204354	33307742	0.90	46568	58599	42902	0.15
H3K4me3^a^	51732192	28850772	0.92	17168	16376	12278	0.6
H3K27ac^a^	42046042	20967712	1.00	30157	55507	21974	0.3
H3K27me3^b^	53134788	na	na	51652	na	na	na
CTCF^a^	86075418	na	na	44922	na	na	na

na, not analyzed.

a Narrow peak.

b Broad peak.

The peaks called show much overlap between the replicates for H3K4me1, H3K4me3, and H3K27ac. The two ChIP-seq experiments were compared using Pearson correlations (Table 4). High correlations between the read coverage of overlapping regions are observed, together with low correlations between the signal values from overlapping peaks for H3K27ac and H3K4me1. H3K4me3 shows a higher correlation of signal values of overlapping peaks.

**Figure 10:**
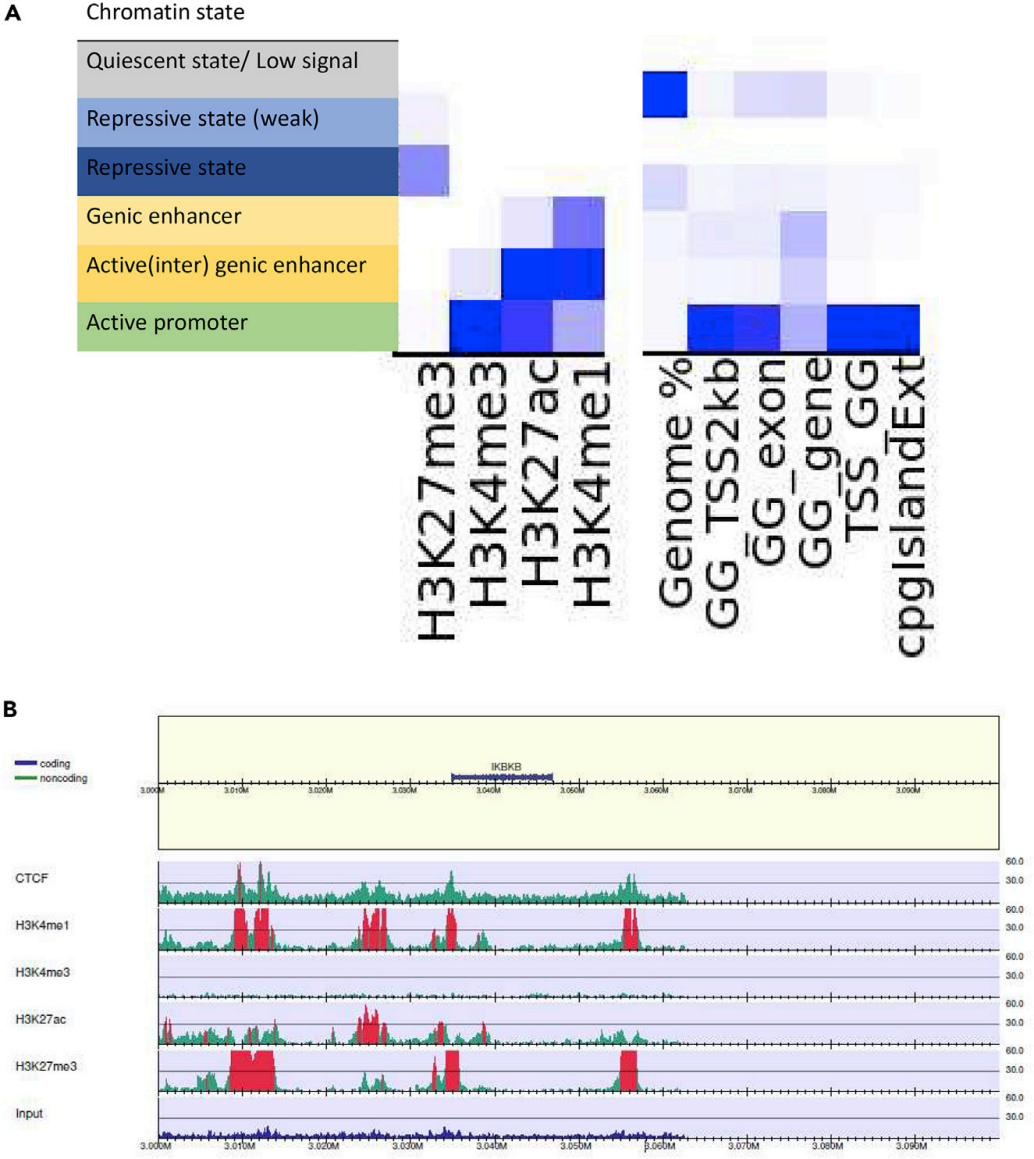
Histone modifications investigated for the chicken SL-29 cell line (A) Six chromatin states were defined using the 4 histone modifications (H3K4me1, H3K27me3, H3K4me3, and H3K27ac), with the left panel describing the chromatin state annotations, central panel showing the emission coefficients in ChromHMM model, and the right panel showing the relative enrichment of coverage in whole genome (genome %) and in different genomic regions(transcription start site (GG_TSS), transcription end site (GG_TES), 2000bp surrounding the TSS (GG_TSS2kb), exon regions (GG_exon), genic regions (GG_gene), and CpG islands. (B) View of the individual histone modification and CTCF profiles in the SL-29 cell line for MICAL1 (TPM = 0.54), TULP1 (TPM = 0.03), and FKBP5 (TPM = 100.06) on chromosome 26.

Various chromatin states were identified, through identification of presence or absence of histone marks using ChromHMM, which provides insight into interactions between different histone marks. Histone marks H3K4me3 and H3K27ac are enriched around the TSS of expressed genes, with H3K4me3 enriched at approximately 20% of the TSS and H3K27ac enriched at >5% of the TSS (Figure S15). The chromatin dynamics (Figure 10A) displays states 2 and 3 as repressed states due to the presence of H3K27me3. States 4, 5, and 6 display active dynamics because these are associated with the presence of H3K27ac/H3K4me1, H3K27ac/H3K4me3, and H3K4me1/H3K4me3.The states identified around 2 kb of the TSS are states 4, 5, and 6, with states 5 and 6 showing a very strong enrichment. State 6 is enriched within exons, whereas states 1, 4, 5, and 6 are enriched within genes. State 6 also shows enrichment within CpG islands. A strong enrichment of H3K4me1 and H3K27ac is seen for the expressed gene *FKBP5* (Figure 10B); enrichments of H3K27me3 are seen around the lowly expressed genes (*MICAL1* and *TULP1*).

Annotation of the peak regions to genomic features provides insight into functional elements related to the histone modifications. As for the histone/CTCF marks in the pig cell line, most marks are found in intron and intergenic regions of the genome. Furthermore, a large percentage (16%–21%) of the H3K4me3, CTCF, and H3K27ac histone modifications are found within promoter regions (Figure S16), and 11% of H3K4me3 has been identified in exon regions of the genome.

Significant consensus sequences for known motifs identified the TFs IRF1, ZNF711, YY2, AP-2alpha, Atf3, and Pax8 for the three histone marks (H3K4me3, H3K4me1, and H3K27ac) (Table 5). In the H3K4me1 consensus sequence of the peak region, a core promoter factor PSE (SNAPc) is observed. In this cell line, a total of 18,516 enhancer regions were identified, and the motif analysis is shown in Data S2. Significant TFs identified within the enhancer regions include SMAD2:SMAD3, EWS-ERG fusion, TWIST1, and TEAD3, which play important roles in regulation of transcription in transforming growth factors and embryonic development and are associated with cancers. The motif sequence for CTCF identified with both homer and MEME (Figure S17) is similar to the human consensus sequence, supporting the conservation of the CTCF-binding site beyond mammals.

**Table 5:** Motif enrichment in histone marks peaks of H3K4me3, H3K27ac, and H3K4me1

Histone mark	Motif	Transcription factor	% of target regions	p-value
H3K4me3		IRF1(IRF)	3.4	1e-232
		OVOL2	58.27	1e-137
		MSANTD3	28.26	1e-105
H3K27ac		ZNF711(Zf)	9.38	1e-86
		YY2	6.05	1e-78
		AP-2alpha(AP2)	13.78	1e-59
H3K4me1		Atf3(bZIP)	3.93	1e-89
		Pax8(Paired, Homeobox)	0.09	1e-42
		PSE(SNAPc)	0.07	1e-42

Top three enriched known binding motifs identified from consensus peaks. Further results shown in Data S2 for motifs with p values <1e-12.

#### Genome wide chromatin accessibility

Chromatin accessibility was profiled in the chicken SL-29 cell line using ATAC-seq data, from which 86,983 peak regions were identified. To infer the functional significance of accessible regions that were identified, consensus peaks were characterized by genomic localization. Annotation of ATAC-seq showed most accessible (open) chromatin is found in the intron and intergenic regions of the genome, (Figure S18), with 12% of accessible chromatin found at promoters (as defined by TSS location). To interrogate the potential function of accessible regions (peaks), they were subjected to a consensus motif enrichment analysis.

Overall, consensus peaks identified for PAX6 recognition sites as most significant, with about 1.74% of accessible regions harboring this consensus motif (Table 6). Roles of the TFs identified are as expected related to this cell line, e.g. PRDM1 TF, which is involved in immunity, and PRDM15, which regulates transcription of WNT, and TFs involved in the MAPK-ERK signaling pathway that is related to pluripotency of a cell.

**Table 6:** Consensus motif enrichment in predicted open chromatin regions

Motif	Known transcription factor motif	% of target regions	p-value
	PAX6	1.74	1e-1706
	IRF6	1.78	1e-1220
	PB0201.1_Zfp281_2	1.62	1e-1184
	PRDM1	1.28	1e-1081
	PRDM15(Zf)	2.06	1e-697
	PB0152.1_Nkx3-1_2	6.81	1e-464
	SF1(NR)	7.81	1e-393
	Zac1(Zf)	11.27	1e-376
	ZBTB26	12.4	1e-335
	ZSCAN29	4.53	1e-329

Top ten enriched known binding motifs identified from the consensus peaks. Related to Data S2.

#### DNA methylation profile

We also determined the average methylation levels for cytosine for the chicken cell line, calculated from both RRBS and WGBS data (Table 7).

**Table 7:** Average methylation levels for different sites between RRBS and WGBS for chicken SL-29 cell line

Site	Assay	Average methylation level (%)
CpG	RRBS	37.44
CHG		0.55
CHH		0.55
CpG	WGBS	59.66
CHG		0.98
CHH		1.05

As expected, average methylation (>10 reads) is observed at CpG sites (37%–59%), and slightly higher average non-CpG methylation (1%–2%) is observed. Average chromosome level methylation levels estimated from WGBS fluctuate around 0.2–0.6 (Figure S19). The average read coverage calculated for the WGBS data was 55.1, with chromosome 20 having a very high (∼95) read coverage (Figure S20).

**Figure 11:**
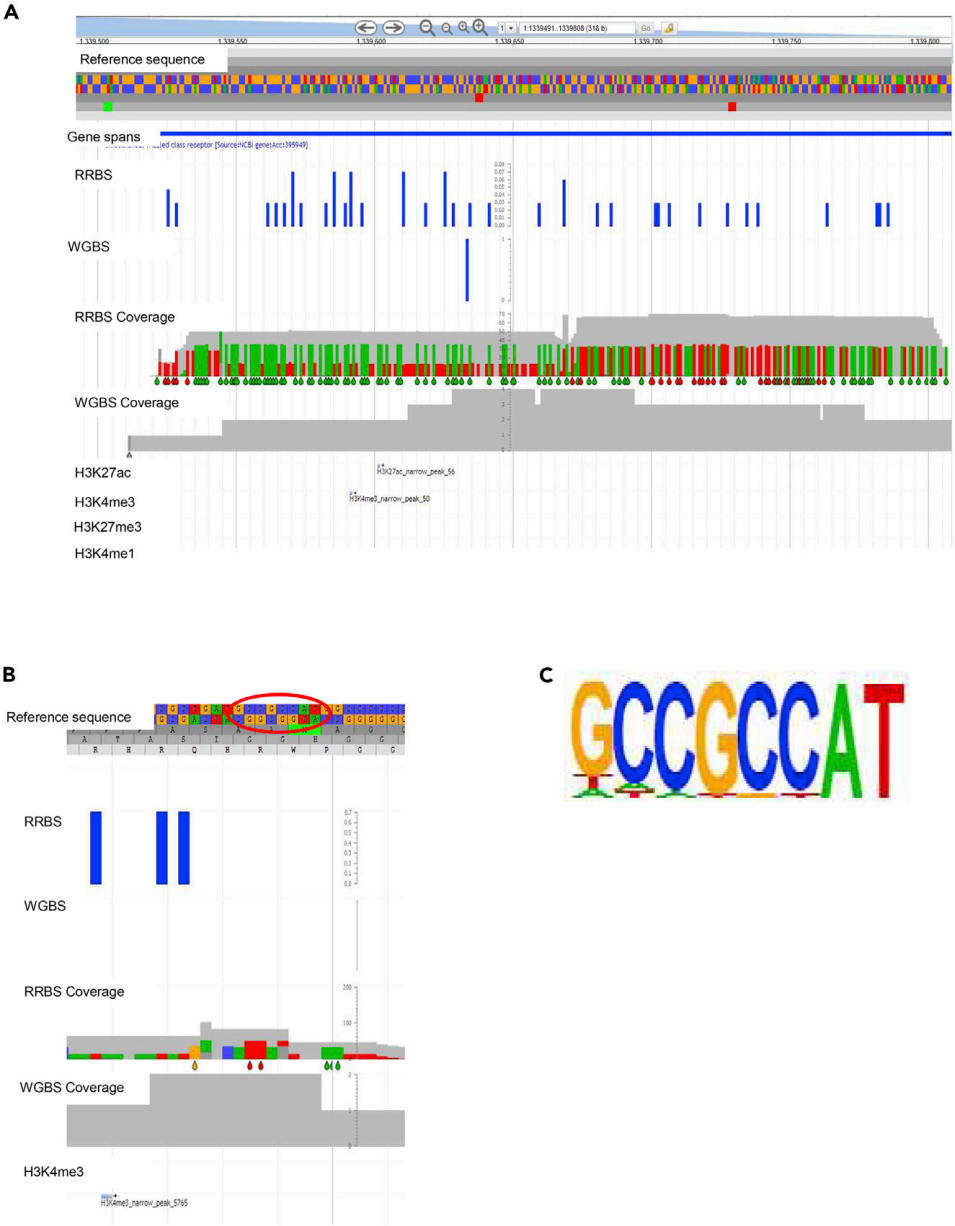
Sites covered by RRBS data while not covered by WGBS (A) Example of a region on chromosome 1 (1339500-1339800) with high RRBS coverage (∼70×) and lower than 10X WGBS coverage (≤4×). This region contains part of the SMO gene (TPM = 210.09), and the histone marks H3K4me3 and H3K27ac are identified and show a peak within this region, which indicates possible promoter/enhancer regions. The histone marks H3K4me1 and H3K27me3 are also displayed here but no peaks were observed within this region. The difference in RRBS and WGBS data coverage is very evident in this example, together with the presence of promoter and enhancer histone marks within a region that is well covered by RRBS data and sparsely covered by WGBS data. (B) Region on chromosome 2 (900,400-900425), with RRBS methylated sites (0.7) at a high coverage (∼83×) and WGBS at a low coverage (2). The H3K4me3 mark is identified here together with (C) the most significant (p-value = 1e-55) motif of identified enhancers (transcription factor YY2).

As stated earlier for the pig, it is of importance to investigate sites covered by RRBS data while not covered by WGBS (and vice versa), as WGBS is considered as the gold standard for insight into whole genome DNA methylation; 926,495 sites were identified by RRBS and not by WGBS in the chicken cell-line (the total number of WGBS sites is 35,805,306 and for RRBS it is 2,830,991). We further investigated if these sites specifically covered by RRBS overlayed regions within predicted promoters and enhancer regions (from the ChIP-seq data). Examples of such regions (chromosome 1 and 2) covered by only RRBS data including predicted active enhancers/promoters are shown in Figure 11.

The transcription factor motif analysis of identified enhancers that overlap with the identified specific RRBS regions is shown in Data S2. One of these motifs, for transcription factor YY2, has a strong CpG consensus sequence, suggesting that these regions only covered by RRBS may include important regions involved in regulation of gene expression.

#### Integrative insight into epigenome marks

**Figure 12:**
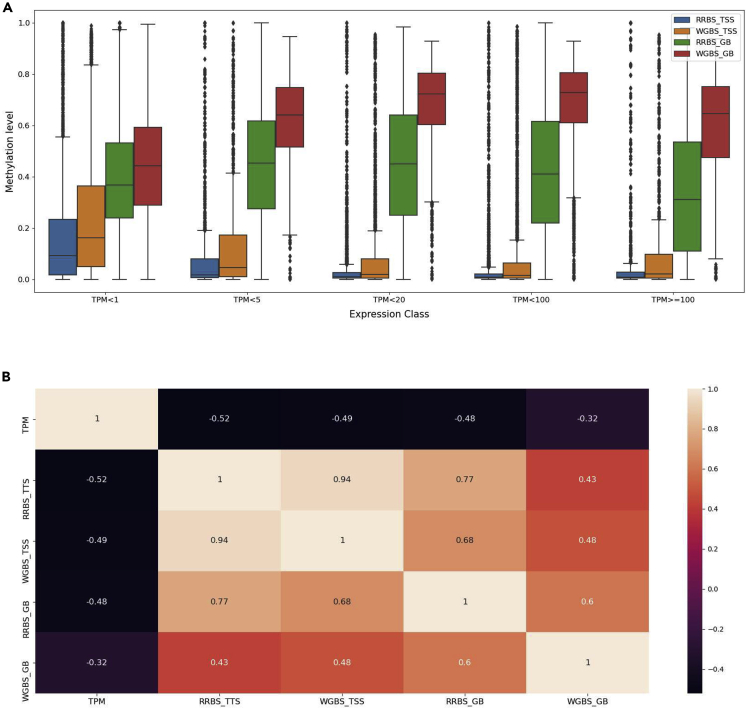
Integrative approach for investigation into regulation of gene expression by epigenomic marks in chicken SL-29 (A) Boxplots of the methylation levels at TSS and GB for RRBS and WGBS data across 5 classes of gene expression levels. (B) Heatmap of the correlations between methylation levels and TPM expression values.

An integrative approach was used to gain insight into the dynamics of methylation and histone modifications for regulation of gene expression (Figure 12). As expected, methylation levels are negatively correlated with gene expression (i.e. highly expressed genes show lower methylation levels and vice versa) at the TSS. Within the gene-body we observe a slight increase in methylation levels with WGBS followed by a decrease with higher expression levels (20 > TPM < 100). The low methylation seen for RRBS within the gene-body can be explained by a lack of coverage in the gene-body compared with WGBS. Heatmap correlations reflect the results observed in the boxplots, with negative correlations between methylation levels both at the TSS and within the gene-body and gene expression.

**Figure 13:**
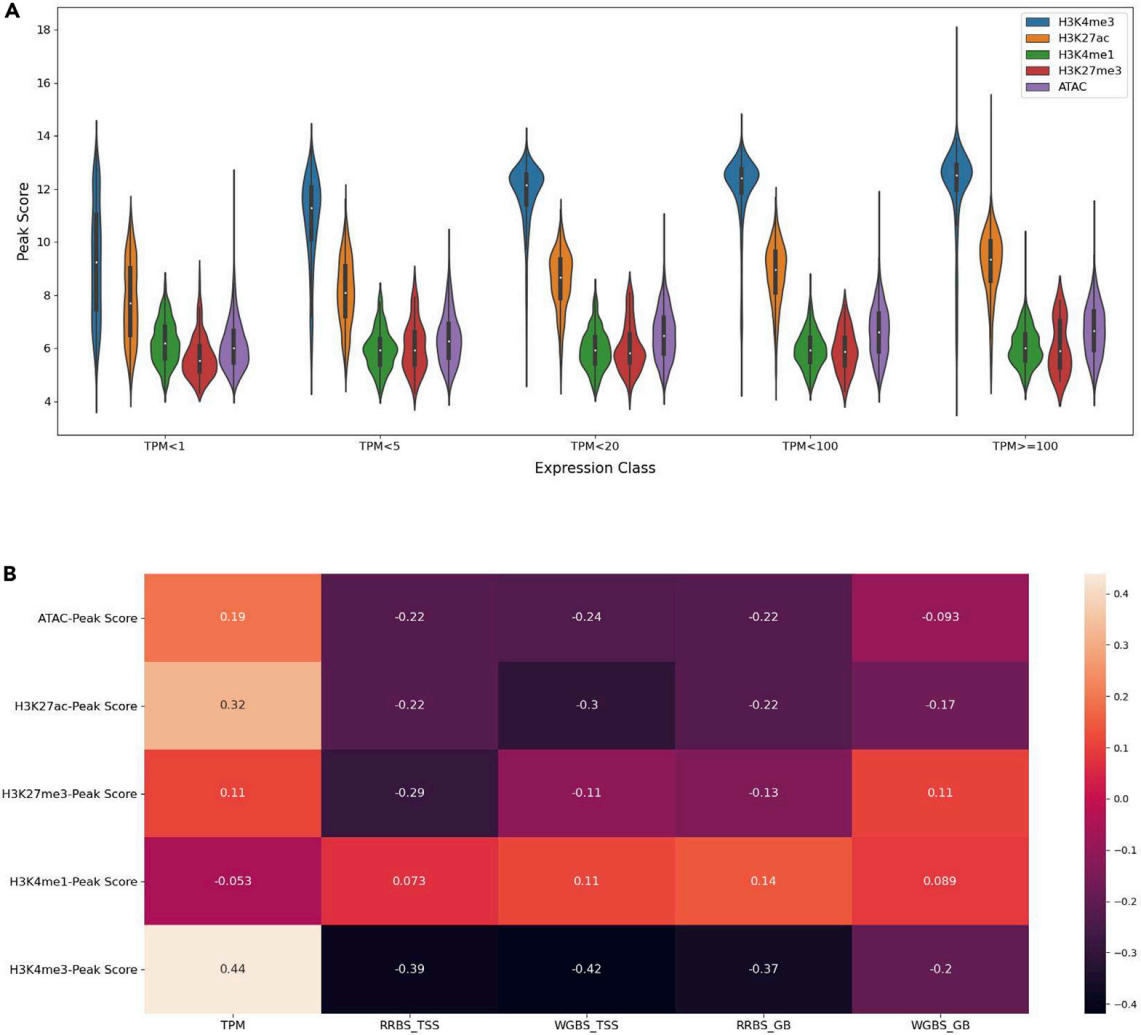
Integrative analysis of histone marks (H3K4me1, H3K4me3, H3K27ac and H3K27me3) and ATAC-seq with gene expression in chicken SL-29 (A) Violin plots of the relationship between the 4 histone marks, ATAC-seq, and 5 classes of gene expression levels (TPM). (B) Heatmap showing the correlations between the 4 histone marks, ATAC-seq, gene expression, and methylation levels of both RRBS and WGBS data at the TSS, as well as gene-body.

The correlations between the 4 histone marks (H3K4me1, H3K4me3, H3K27ac, and H3K27me3), ATAC-seq, and distinct classes of gene expression levels are visualized in Figure 13. Enhancer histone marks, H3K27ac and H3K4me3, together with ATAC-seq show an increase in peak score for genes with a higher gene expression level. H3K4me1 and H3K27me3 show little variation in peak scores across different classes of gene expression. For H3K27me3, slightly lower peak scores were observed for very highly expressed genes, whereas higher peak score were seen for very lowly expressed genes.

The positive correlation observed for H3K4me3 and H3K27ac with the gene expression (Figure 13B) is higher compared with the methylation and gene expression results presented in Figure 12B. Negative correlations are observed between methylation levels at both the TSS and gene-body for RRBS and WGBS with promoter and enhancer marks (H3K4me3, H3K27ac), as well as with ATAC-seq. Low correlations between the histone marks H3K4me1 and H3K27me3 with methylation are observed. An example of a region with genes and all epigenomic modifications is shown is Figure S21.

## Discussion

Cell lines provide an ethical approach for research in animal production, and thus, molecular characterizations are necessary for functionally relevant cell lines. The pig IPEC-J2 and chicken SL-29 cell lines have never been characterized using an integrative genomics approach. These two cell lines were chosen specifically for their usability and application to research in animal production as well as biomedical research. Using different omics data (WGS, RNA-seq, ChIP-seq, ATAC-seq, RRBS, WGBS), the genome structure, transcriptome, methylome, and chromatin accessibility were investigated and characterized for these cell lines; this provides a reference of the genome architecture of these cell lines for future functional studies using these cell lines as well as for farm animal research community.

Results for both pig and chicken cell lines show that aneuploidy is common in both cell lines, as we observed various chromosomes that were either (partly) monoploid, triploid, and even tetraploid. More aneuploidy as well as more structural variants were observed for the chicken SL-29 cell line in comparison with the pig IPEC-J2 cell line. For SV this could be due to the additional filtering of common structural variants from the pig cell line. Previous studies have shown that more chromosomal abnormalities, as well as structural variants occur when cell lines are maintained in culture for a longer time (more passages),^15^^,^^16^ emphasizing the importance of limiting the number of cell passages for cell line experiments. It has also been suggested that the culture conditions can influence the chromosomal stability.^17^ Conditions such as techniques for cell detachment and disaggregation and oxygen concentration during culture can also affect the chromosomal stability and genomic integrity over a longer period of culture.^18^ To ensure that conditions do not influence the genomic integrity, precisely defined protocols for cell culture should be followed as much as possible.

The increase in ploidy leads to an increase in expression of the genes on these chromosomes, likely affecting functional relevant aspects of these cell lines. A comparison of the results from the pig cell line with similar tissue type and organoid samples showed pronounced higher rates of expression of the genes on the triploid chromosome 17 of the pig cell line. From this comparison it is evident that these cell lines show higher rates of gene expression on all chromosomes, followed by organoids, and with tissue showing the lowest rates of gene expression; this is in agreement with studies showing that organoids resemble gene expression levels and physiology of tissue more closely than cell lines.^19^^,^^13^ It has also been shown that aneuploidies and structural variants can influence gene expression level; specifically, structural variants can cause changes in *cis*-regulatory elements, promoters, and enhancers.^20^^,^^21^^,^^22^ These expression observations in cell lines provide a useful resource for studies where potential genes of interest can be identified and investigated for increased expression levels.

The results from the WGS and RNA-seq show the potential of using these assays to detect chromosomal abnormalities, in addition to investigation of variation in the genome^23^^,^^24^ and gene expression, respectively. Traditional methods such as karyotyping and staining (multifluor-fluorescence in-situ hybridization) are limited to detection of chromosomal abnormalities, specifically chromosomal rearrangements such as translocations, and provide little insight into genome variation.^25^ WGS as a tool for detection of pre- and postnatal anomalies is investigated and implemented more regularly.^26^^,^^27^ An example of using NGS (next-generation sequencing) for detection of prenatal anomalies is discussed in Guseh 2020^28^, where trisomy 21 is detected when a higher proportion of DNA fragments are mapped to chromosome 21 in comparison to a Ref. 28; this shows the potential of WGS for detection of chromosomal abnormalities.

Chromatin accessibility and histone modifications were investigated to gain further insight into the genome architecture of both cell lines using ChIP-seq data for CTCF and four histone modifications: H3K4me1, H3K4me3, H3K27ac, H3K27me3. The importance of using a standardized protocol for comparative ChIP-seq studies was explored using two technical replicates of the ChIP-seq experiments for three histone marks, performed in two different laboratories and using different inputs as a background (immunoglobulin G [IgG] and DNA, respectively); this provides insight into the reproducibility of results between different laboratories and the use of DNA or IgG as an input. We conclude there is a high consensus between overlapping peaks of the experiments, and secondly, the read coverage between experiments showed medium to high consensus between experiments. However, signal values show little correlation between experiments, suggesting that the confidence related to the high number of overlapping peaks is limited and should therefore be used with care if the signal values are used for comparative analysis. A reason for this low correlation of signal values could be the differences in background signal in the two experiments. ChIP-seq guidelines and practices from (mod)ENCODE have found that an IgG control mimics a ChIP experiment more closely than a DNA input control. Cases of strong sonication bias are rarely observed, but this can potentially affect peak calling.^29^ Thus, for comparative studies utilizing ChIP-sequencing, similar protocol should be followed.

Peak regions identify possible binding sites of proteins associated with DNA (protein-chromatin interactions) and provide insight into regulatory regions and elements. The number of histone marks identified are different between tissues, stages of development, and number of reads sequenced. ENCODE standards have shown that the number of peaks that can be identified ranges from thousands to tens of thousands of peaks.^29^^,^^30^ Coincidentally the number of peak regions called for narrow peaks (H3K4me3 and H3K27ac) for both pig and chicken cell lines was similar to that in previous studies in monogastric species.^31^^,^^32^ We confirmed the quality of the data by investigating the occurrences of the respective marks around 2 kb of the TSS. We observed an elevation of the marks H3K4me3 and H3K27ac around the TSS. It has been observed that H3K4me3 is most often found at TSS (regardless of H3K27ac) and indicative of a promoter, as seen in this study. H3K4me3 is often co-occupied by H3K27ac in the genome^33^^,^^34^^,^^35^; however, H3K27ac is not always found to be co-occupied by H3K4me3 and is also observed further from the TSS site. H3K4me1 and H2K27ac are indicative for enhancer regions.^33^ The number of broad peak regions of H3K27me3 for both cell lines is similar to that observed in other vertebrate species, with variations between tissues.^36^ This mark is associated with gene silencing, as shown in chromatin state analyses.^34^^,^^35^ Combinations of histone modifications result in variable chromatin structures, leading to different levels of transcription, which is also reflected in the integrative analysis.

To evaluate the quality of the experiments and the success thereof, we compared the consensus motif for the CTCF sites identified for pig and chicken with the human CTCF consensus sequence. There was good similarity between the consensus motifs for both pig and chicken and human. Furthermore, this motif was also identified in the human K562 cell line as a CTCF mark. CTCF is a highly conserved protein in mammals (between pig and mouse) as well as in vertebrates,^37^^,^^38^^,^^39^ which is indeed confirmed by our CTCF results, supporting the good quality of our results for CTCF.

Further investigation into motifs identified for the histone marks in both cell lines, together with comparison of similarities thereof to known motifs, provides insight into possible TFs. Firstly, interferon receptor factor (IRF) was identified in both cell lines (pig IRF2 and chicken IRF1). IRF is part of a TF family that is found in humans as super enhancer TFs and is highly conserved within species.^40^ This TF plays a role in immunity, cell growth, differentiation, and anti-tumor defences in vertebrates.^41^^,^^42^^,^^43^ TFs such as YY2, TEAD, and E2F, which regulate cell growth and proliferation as well as development were also identified.^44^^,^^45^

Open chromatin was investigated using ATAC-seq data for the chicken cell line with a number of regions identified, slightly lower than expected when following ENCODE recommendations, which suggests >100,000 peaks. However, this is similar to the number of enriched regions identified by other studies in animals.^46^^,^^47^ Most of the accessible chromatin was identified within intronic, intergenic, and promoter regions, which is in line with previous research in multiple species and suggests similar patterns between cell lines and tissues.^46^^,^^47^

Further insight into gene expression and the molecular characteristics of both cell lines was obtained through characterization of the methylome, which ensures comprehensive characterization of the functional genome. DNA methylation is an epigenetic mark that is found in most species and has been found to be inherited and influenced by environmental factors (and often used for comparative analysis and fundamental research into e.g. adaptation).^48^^,^^49^ In both cell lines a higher coverage in the methylation data (RRBS and WGBS) was observed on aneuploid chromosomes, relating to the change in copy number that is reflected in the methylome as well as on a whole genome level. Methylated cytosines occur primarily at CpG sites in most cell types, whereas non-CpG methylation (CHG, CHH) occurs only in specific cell types such as brain, oocytes, and stem cells^50^; this is in agreement with our observation in our cell lines, where CpG methylation is the primary type of methylation. In addition, the non-CpG methylation levels in the chicken cell line are slightly higher than observed in tissues of birds, excluding brain tissue.^51^^,^^52^ This occurrence could be due to the chromosomal aberrations and higher ploidy observed and the cell lines behaving in a tumorigenic manner. Ploidy effects on DNA methylation (epigenome) have been theorized in studies on plants^53^^,^^54^ and tumors.^55^^,^^56^ We observed regions within the genome covered by RRBS that were not identified by WGBS or had a low coverage (<10 ×) by WGBS. Some of these regions are highly relevant, as they are located at the promoter of specific genes (close to the start site of the gene) or overlapping with enhancer elements. It is noteworthy that the coverage of the WGBS data is high (>50×) and therefore should theoretically cover all of the genome, especially at informative sites.^57^ This is a relevant observation, as WGBS is often seen as the gold standard for investigating the methylome, as it is supposed to cover almost all sites in contrast to RRBS, which is seen as a cost-effective alternative method. RRBS usually shows reduced coverage of methylated sites in intergenic and distal regulatory regions, especially in comparison with WGBS^48^ but our study suggests that RRBS is complementary to WGBS, and to obtain the most comprehensive genome-wide estimation of DNA methylation the two methods should be combined.

Finally, we attempted to integrate the various epigenetic marks together with gene expression to show how the functional genome regulates expression levels. Correlations between the expression data and methylation indicate that promoter methylation has a reverse relationship with gene expression, with, as expected, methylated sites inhibiting gene expression. Interestingly, the methylation level within the gene-body in the pig cell line increases slightly with higher expression levels, whereas the methylation in the gene-body of the chicken cell line decreases slightly with increased expression levels; this is similar to what has been observed within the gene-body in earlier studies of the methylome in pig and avian species.^14^^,^^51^ This phenomenon has not been studied extensively; however, Derks et al.^51^ suggested possible explanations such as methylation suppressing the transposable elements (TE) and preventing TE insertions, which can be interruptive in the genome. A reason for this could be the higher number of TE in mammals compared with birds, which require higher methylation levels in gene bodies. As expected, the histone modification H3K27me3, which is associated with gene silencing, shows a strong positive correlation to lowly expressed genes and a negative correlation to highly expressed genes. Histone modifications associated with promoter and enhancer regions all show a positive correlation to highly expressed genes and a negative correlation to lowly expressed genes; this confirms previous studies regarding these epigenetic marks regulating gene expression levels.

### Conclusion

This paper is the first to describe the molecular characteristics (structure) of the pig IPEC-J2 and chicken SL-29 cell lines. The genomic approaches provided an insight into the different levels of the epigenome influencing gene expression in these cell lines, as well as provided a description of the architecture of the epigenome. Chromosomal abnormalities, copy-number variations, and aneuploidy, typical for a cell line, were identified for several chromosomes for both cell lines. These cell lines are referred to as nontumorigenic and nontransformed; however, as these cells go through many passages, aneuploidy events do occur. Future researchers should note the characteristics of these cell lines and proceed with caution for interpretation of results. Epigenetic marks such as histone modifications, chromatin accessibility, and DNA methylation were integrated with expression data for both cell lines; this provided insight into the interactions between the epigenetic marks and gene expression. The characteristics as described in this paper for these cell lines will be similar for cells cultured using the same protocol and cells grown for the same number of passages. Deviations from these guidelines/methodologies are expected to result in different genomic and epigenomic characteristics. Understanding these cell lines and the (epi)genetic makeup thereof can provide a better understanding of the limitations of these cell lines as a model for *in vivo* research. We propose these cell lines as a reference for future functional and comparative studies in animals, whereby knowledge of ploidy, expression profile, chromatin landscape, and methylome provide the backbone for the comparison.

### Limitations of the study

The ATAC-seq data were unavailable for the pig IPEC-J2 cell line. Further investigation using traditional methods for confirmation chromosomal abnormalities e.g. karyotypes staining would be beneficial.

## STAR★Methods

### Key resources table

**Table:** 

REAGENT or RESOURCE	SOURCE	IDENTIFIER
**Antibodies**		

insulator-CTCF antibody	Milipore	RRID:AB_11212315
H3K4me1 antibody	Abcam	RRID:AB_306847
H3K4me3 antibody	Diagenode	RRID:AB_2924768
H3K27ac antibody	Abcam	RRID:AB_2118291
H3K27me3 antibody	Diagenode	RRID:AB_2753161

**Critical commercial assays**		

HiSeqX	Illumina	Cat#SY-412-1001
Hi-Seq 4000	Illumina	Cat#SY-401-4001
All Prep DNA/RNA Mini Kit	Qiagen	Cat#80284
TruSeq RNA sample preparation kit	Illumina	Cat#FC-122-1001; Cat#FC-122-1002
Ovation RRBS library	NuGEN	Cat#0353; Cat#0553
TruSeq SBS sequencing kit version 4	Illumina	Cat#FC-401-4003
EZ DNA Methylation Gold Kit	Zymo Research	Cat#D5005; Cat#D5006
HiSeqX S4	Illumina	NA
Bioruptor Pico sonicator	Diagenode	Cat#B01060010
PureProteome Protein A and G magnetic beads	Millipore	Cat#LSKMAGAG10
MinElute PCR Purification columns	Qiagen	Cat#28004
Kapa Hyper Prep Kit	Illumina	Cat#KK8500
QIAquick MinElute	Qiagen	Cat#28004
E-gel iBase	Invirtogen	Cat#G6300
Bioanalyzer 2100 system	Agilent	Cat#G2939BA
HiSeq2000	Illumina	Cat#SY-401-1001
Nextera primers	Ilumina	Cat#G2939BA
KAPA HiFi HotStart ReadyMix (2x)	Kapa Biosystems	Cat#50-196-5217
Qubit 2.0 fluorometer	Life Technologies	Cat#Q32866
Covaris S220 ultrasonicator	Covaris	Cat#500217
Qubit fluorometer	ThermoFisher Scientific	Cat#Q33238
iQ SYBR Green Supermix	Bio-Rad	Cat#1708880
NextFlex adapters	Bioo Scientific	Cat#5118-01
HiSeq2500	Illumina	Cat#SY–401–2501

**Deposited data**		

Pig Jejenum organoid at 3 weeks	ENA	ENA:SAMN14300031
Pig Jejenum organoid at 12 weeks	ENA	ENA:SAMN14300021
Pig Jejenum tissue at 5 weeks	ENA	ENA:SAMN14300018
IPECJ87	ENA	ENA:SAMN14300016
IPECJ91	ENA	ENA:SAMN14299997
IPECJ2 genomic assays	ENA	ENA:PRJEB59474
SL29 genomic assays	ENA	ENA:PRJEB59475
RRBS	ENA	ENA:ERA20415761 (IPECJ2); ENA:ERA20425033 (SL-29)
WGBS	ENA	ENA:ERA20417079 (IPECJ2); ENA:ERA20425013 (SL-29)
ChIP-seq	ENA	ENA:ERA20424890 (IPECJ2); ENA:ERA20425073 (SL-29)
ChIP-seq experiment 2	ENA	ENA:ERA20425077 (IPECJ2) ; ENA:ERA20425081 (SL-29)
ATAC-seq	ENA	ENA:ERA20425080
WGS	ENA	ENA:ERA20424869 (IPECJ2); ENA:ERA20425048 (SL-29)
RNA-seq	ENA	ENA:ERA20424896 (IPECJ2); ENA:ERA20425060 (SL-29)

**Experimental models: Cell lines**		

IPEC-J2 cells	DSMZ	Cat# ACC-701; RRID:CVCL_2246
SL-29 cells	ATCC	Cat# CRL-1590; RRID:CVCL_5587

**Software and algorithms**		

Sickle (v1.33)	Joshi NA, 2011^58^	https://github.com/najoshi/sickle
bwa mem (v0.7.15)	Li, 2013^59^	https://github.com/lh3/bwa
Samblaster (v0.1.26)	Faust and Hall^60^	https://github.com/GregoryFaust/samblaster
Samtools (v1.9)	Li et al.^61^	https://github.com/samtools/samtools
Qualimap (v2.2.1)	García-Alcalde et al.^62^	http://qualimap.conesalab.org/
tinycov package (v0.3.0)	tinycov · PyPI,” n.d.	https://pypi.org/project/tinycov/
FreeBayes (v1.3.1)	Garrison and Marth^63^	https://github.com/freebayes/freebayes
Circos (v-69-9)	Krzywinski et al.^64^	http://circos.ca/
Manta (v1.4.0)	Chen et al.^65^	https://github.com/Illumina/manta
CNVnator (v 0.3.3)	Abyzov et al.^66^	https://github.com/abyzovlab/CNVnator
Variant effect predictor (VEP)	ENSEMBL ; McLaren et al.^67^	https://www.ensembl.org/info/docs/tools/vep/index.html
TrimGalore (v0.6.4)	Martin, 2011^37^	https://github.com/FelixKrueger/TrimGalore
FastQC v0.11.9	Andrews, 2010^68^	https://www.bioinformatics.babraham.ac.uk/projects/fastqc/
RSEM with STAR v2.7.3a	Li and Dewey^69^; Dobin et al.^70^	https://deweylab.github.io/RSEM/: https://github.com/alexdobin/STAR
bowtie2 (v2.3.2)	Langmead and Salzberg^71^	https://bowtie-bio.sourceforge.net/bowtie2/index.shtml
FilterbyTile (v38.20)	BBMap package; Bushnell, n.d.^72^	https://sourceforge.net/projects/bbmap/
MACS2 (v2.7.1)	Feng et al.^73^; Zhang et al.^74^	https://github.com/macs3-project/MACS
deeptools (v3.1.3)	Ramírez et al.^75^	https://deeptools.readthedocs.io/en/develop/
ChromHMM (v1.22)	Ernst and Kellis,^76^^,^^77^	http://compbio.mit.edu/ChromHMM/
MEME-ChIP and MEME from MEME-suite (v5.2.0)	Bailey et al.^78^; Machanick and Bailey,^79^	https://meme-suite.org/meme/
HOMER (v4.1.0)	Heinz et al.^80^	http://homer.ucsd.edu/homer/ngs/annotation.html
DROMPA	Nakato and Shirahige,^81^	http://nakatolab.iqb.u-tokyo.ac.jp/softwares/drompa/index.html
BEDtools (v2.30.0)	Quinlan and Hall,^82^	https://bedtools.readthedocs.io/en/latest/
picard (v2.23.9)	“Picard Tools - By Broad Institute,” n.d.^83^	https://broadinstitute.github.io/picard/
BSseeker2 v2.1.8	Guo et al.	https://github.com/BSSeeker/BSseeker2
CGmaptools (v0.1.2)	Guo et al.^84^	https://cgmaptools.github.io/
MethylKit (v1.16.1)	Akalin et al.^85^	https://www.bioconductor.org/packages/release/bioc/html/methylKit.html#:∼:text=methylKit%20is%20an%20R%20package,and%20whole%20genome%20bisulfite%20sequencing.
Custom scripts	de Vos, 2021^86^	https://github.com/Jani-94/scripts; https://doi.org/10.5281/zenodo.7274310

**Other**		

Pig reference	ENSEMBL Sus Scrofa 11.1	https://www.ensembl.org/Sus_scrofa/Info/Index?db=core
Chicken reference	ENSEMBL *Gallus gallus* GRCg6a	https://apr2022.archive.ensembl.org/Gallus_gallus/Info/Index
Pig annotation	ENSEMBL Sus Scrofa 11.1 - release 103	https://ftp.ensembl.org/pub/release-108/gtf/sus_scrofa/Sus_scrofa.Sscrofa11.1.108.chr.gtf.gz
Chicken annotation	ENSEMBL *Gallus gallus* GRCg6a - release 103	https://apr2022.archive.ensembl.org/Gallus_gallus/Info/Index

### Resource availability

#### Lead contact

Further information and requests for resources and reagents should be directed to and will be fulfilled by the lead contact, Jani de Vos (jani.devos@wur.nl).

#### Materials availability

This study did not generate new unique reagents.

#### Data and code availability

• All data types (WGS, RNA-seq, WGBS, RRBS, ChIP-seq – H3K4me1, H3K4me3, H3K27ac, H3K27me3, CTCF and ATAC-seq) from pig IPECJ2 (PRJEB59474) and chicken CRL (PRJEB59475) cell lines have been deposited at ENA and are publicly available as of the date of publication. Accession numbers are listed in the key resources table.
• All original code has been deposited at Zenodo (https://doi.org/10.5281/zenodo.7274310) and is publicly available as of the date of publication. DOIs are listed in the key resources table.
• Any additional information required to reanalyze the data reported in this paper is available from the lead contact upon request.

### Experimental model and subject details

The intestinal epithelial pig IPECJ2 (ACC-701) cell line was obtained from the cell repository at DSMZ,^87^ which is an intestinal columnar epithelial cell line derived from the mid-jejunum of a neonatal unsuckled female piglet (piglets less than 12 hours old). These cells were originally isolated in 1989 by Helen Berschneider at the University of North Carolina.^88^ For chicken the SL-29 cell line (ATCC CRL-1590), was derived from embryonic fibroblast cells obtained from the cell repository at ATCC.^89^ Cells were cultured at 37°C and 5% CO2 in Dulbecco’s MEM with 5% FBS, Pen/Strep and Glutamax using a standard FAANG operating procedure. The media was refreshed twice a week and progressing to the next passage mostly 1/20 of the cells were transferred to a new flask. These cells were cultured for 4 passages in chicken SL-29 and 67 passages in pig, before harvesting.

### Method details

#### Sequencing and assays

These cells were then used for whole genome sequencing (WGS), RNA sequencing, reduced representation bisulphite sequencing (RRBS), whole genome bisulphite sequencing (WGBS), ChIP-seq and ATAC-seq. DNA and RNA were isolated from the cell lines using the All Prep DNA/RNA Mini Kit (Qiagen) following manufacturer’s instructions. WGS libraries of ∼ 300-400 bp fragments were prepared using Illumina paired-end kits (Illumina, San Diego, CA) and 150 bp paired-end sequenced with Illumina HiSeqX. RNA-seq library preparation and sequencing was done as described in van der Hee et al.^13^ using TruSeq RNA sample preparation kit (Illumina), incorporated within the Novogene manufacturer’s protocol. Thereafter, samples were sequenced with Illumina Hi-Seq 4000 producing raw data with 150 bp paired-end reads. RRBS was done as described in Corbett et al.^90^ In brief, DNA was fragmented using the *MSPI* restriction enzyme followed by a 20-250 bp fragment size selection and library preparation using the Ovation RRBS library (NuGEN). Samples were pooled and sequenced with the TruSeq SBS sequencing kit version 4 on the HiSeq 2500 (Illumina). A biological replicate was also sequenced following the same procedure for both pig IPECJ2 cell line. For WGBS genomic DNA was spiked with lambda DNA, fragmented by sonication to 200-400 bp with Covaris S220 (Covaris, Inc., Woburn, MA, USA), followed by end repair and A-ligation. Cytosine-methylated barcoded adapters were ligated to the sonicated DNA. The DNA bisulfite conversion was performed using the EZ DNA Methylation Gold Kit (Zymo Research, Irvine, USA). DNA fragments were size selected and amplified using the KAPA HiFi HotStart ReadyMix (2X) (Kapa Biosystems, Wilmington, USA). Library concentration was quantified using a Qubit 2.0 fluorometer (Life Technologies, Carlsbad, USA) and qPCR (iCycler, BioRadLaboratories, Hercules, USA). Libraries were sequenced using the HiSeqX S4 flow cell with PE150 strategy.

ChIP-seq for both cell lines was performed for the insulator Anti-CTCF (polyclonal antibody lot # 2887267; Millipore) and histone marks H3K4me1 (polyclonal antibody ChIP grade ab8895; Abcam), H3K4me3 (polyclonal antibody lot # A1052D; Diagenode), H3K27ac (polyclonal antibody ChIP grade 4729; Abcam) and H3K27me3 (polyclonal antibody, lot # a1811-001P; Diagenode). These histone marks were chosen as they provide insight into transcriptional activation and the location of enhancers and promoters. ChIP-seq data sets were generated in two different laboratories (two replicates; experiment 1 and 2) for each cell-line. As input control in the first experiment, an IgG “mock” control was used, whereas in experiment two, an “input” DNA control was used for the ChIP-seq studies. In the second experiment only three histone marks (H3K4me1, H3K4me3 and H3K27ac) were assayed. The same ChIP-seq protocol was applied in both laboratories. Chromatin preparation was performed where cells (cultured in petri-dishes) were cross-linked with 1.1% formaldehyde for 10 min, stopped by adding 1/10 vol of 1.25 M Glycine for 2 min and washed with cold PBS. Cells were harvested by scraping, incubated with different buffers and finally resuspended in an incubation buffer with PIC with a final concentration of 15 million cells/ml. Shearing of the cells was performed in 300 μl cell suspension /tube with 10 cycles 30 seconds on and 30 seconds off at 4°C using the Bioruptor Pico sonicator (Diagenode). Lastly the sonicated material was divided into aliquots and stored at -80 °C. The overnight immunoprecipitation step with the different antibodies at 4°C was performed on the chromatin using 4.5 million cells as input per antibody. Immunoprecipitated chromatin was incubated overnight with a 50:50 mix of PureProteome Protein A and G magnetic beads (Millipore). The beads were washed (6 washes with 4 wash buffers), rotated and de-cross-linked. The de-cross-linked DNA was finally purified (MinElute PCR Purification columns (Qiagen)), and DNA quantities were determined with Qubit fluorometric quantification (ThermoFisher Scientific). A qPCR analysis of ChIP DNA was performed with iQ SYBR Green Supermix (Bio-Rad) on a CFX96 Real-Time System C1000 Thermal Cycler (Bio-Rad). Library prep was performed using the Kapa Hyper Prep Kit for Illumina sequencing using the manufacturers protocol, with the following adjustments. DNA was used as an input together with NextFlex adapters (Bioo Scientific), followed by PCR amplification. Post-amplification cleanup was performed using QIAquick MinElute columns (Qiagen) and library size selection (300-bp fragments) was performed using the E-gel iBase (Invirtogen). Thereafter the quality and quantity of the library was examined using a High Sensitivity DNA Chip on a Bioanalyzer 2100 system (Agilent). Finally, the libraries were paired-end sequenced using Illumina high-throughput sequencing protocol on a HiSeq2000 (Illumina). For the second experiment sequencing was performed on a HiSeq4000 (Illumina).

Lastly ATAC-sequencing was completed following the Fast-ATAC-sequencing protocol as described in Corces et al.*,* 2016, with the following exceptions we used 25k cells as input and the standard Illumina Nextera primers for library amplification. These libraries were sequenced on a HiSeq4000 (Illumina), paired-end with 150bp.

#### Data analysis

Pig (Sus Scrofa 11.1) and chicken (*Gallus gallus* GRCg6a) reference genomes, together with ENSEMBL annotations (Sus Scrofa 11.1 - release 103 & *Gallus gallus* GRCg6a - release 103) were utilized for all data analyses of our study. Default settings were used unless otherwise stated, and a brief overview of the data analyses is shown in Figure S1. Genome indexes were built using the required reference genomes (and annotations where required) with the tools described below. Quality of all datasets was evaluated, and the statistics thereof is shown in Tables S1 and S2.

#### Whole genome sequence analysis

Whole genome sequences were trimmed using Sickle v1.33^58^ in paired-end mode, where a sliding window approach was used for trimming adapters. Alignment of the trimmed reads together with removal of duplicates was completed using bwa mem (v0.7.15)^59^ together with Samblaster (v0.1.26).^60^ The aligned reads were further processed using samtools (v1.9)^61^ to fill in mate coordinates, as well as add requirements from mate related flags. Mapping quality was evaluated using Qualimap (v2.2.1)^62^ to ensure correct and accurate mapping.

Read-depth, genome structure, and possible large structural variants were evaluated using the tinycov package (v0.3.0)^91^ and SNV calling was done using FreeBayes (v1.3.1).^63^ Read support (ratio) was evaluated for heterozygous variants within the VCF file using a custom unix script^86^ and the results were plotted as histograms. The read-depth and SNV’s were then plotted using Circos (v-0.69-9)^64^ for the visualization at a whole genome level, as well as for specific regions of interest.

Structural variant analysis of both cell lines was completed using Manta (v1.4.0).^65^ For the pig cell line these variants were filtered in the following way: structural variants from healthy pig tissue samples with similar high read depth (in house samples: two muscle and one liver) were identified using Manta and overlapping structural variants between cell-lines and tissues were filtered out from the cell line structural variants. This strategy was used to exclude naturally occurring structural variants not unique to the cell line. For chicken Manta analysis this strategy was not possible due to lack of WGS chicken data with sufficient high coverage. Large SVs (deletions and duplications) were investigated using CNVnator (v0.3.3),^66^ and results verified using a genome browser.^92^ Variant Effect Predictor^67^ was used to determine the consequences of all copy number and structural variants on the genomes.

#### RNA-sequencing analysis

Stranded RNA-seq datasets for both cell lines (pig IPECJ2 and chicken SL-29) were trimmed for adapters, quality and minimum length using TrimGalore v0.6.4 a wrapper for Cutadapt v1.18^93^ and the sequence data quality was evaluated using FastQC v0.11.9.^68^ The trimmed reads were used for alignment and gene quantification using RSEM,^69^ with STAR v2.7.3a as aligner.^70^ Further analyses were completed using custom shell scripts for basic statistics and average gene expression level per chromosome was calculated and plotted using a custom python script with the Seaborn package.^86^ Various minimum transcript per kilobase million (TPM) thresholds were implemented for different analyses to reduce noise of uninformative genes that are very low expressed.

Additional raw RNA-sequencing data was downloaded from ENA from the PRJNA610529 project: two pig jejunum organoid samples grown for different time periods (3 weeks (ENA:SAMN14300031) and 12 weeks (ENA:SAMN14300021), a 5 week old pig jejunum tissue sample (ENA:SAMN14300018), cell lines IPECJ87, an IPECJ2 cell line grown for 87 passages (ENA:SAMN14300016), and IPECJ91, an IPECJ2 cell line grown for 91 passages (ENA:SAMN14299997). We trimmed, aligned, and completed gene quantification of this data following the same procedure as the above procedure used for the IPECJ2 cell line used in this study. These samples were used to compare the average gene expression level per chromosome in jejunum tissue, organoid and IPECJ2 cell line.

#### ChIP-sequencing analysis

Raw reads were trimmed with TrimGalore v0.6.4 a wrapper for Cutadapt v 1.18^93^ for adapter sequences, length, and quality. Reads for the different marks were aligned using bowtie2 (v2.3.2).^71^ Secondly, reads with red label (very low) on “per tile sequence quality” metric of FastQC were scanned with FilterbyTile (v.38.20) from BBMap package.^72^ FilterbyTile increases the quality of Illumina reads, which are dependent on location in a flow cell. Moreover, the reads of the second experiment were truncated to match the read length of the first experiment (36bp) allowing better comparison of the two. Samtools was used on the aligned reads for conversion of the alignment into BAM format, sorting, removing PCR duplicates, and keeping only paired-end reads, as well as uniquely aligned reads.

Peak calling for the respective marks, was completed using MACS2 (v2.7.1),^74^^,^^73^ with the input (IgG and DNA respectively) used as the negative control. Visualization of the marks around the transcription start site (TSS) of expressed genes (TPM>1) was achieved using plot enrichment from deeptools (v3.1.3).^75^ ChromHMM (v1.22)^76^^,^^77^ was used for the identification of different chromosome states based on interactions between marks, and the interaction around the TSS. Motif-based sequence analysis with MEME suite specifically MEME-ChIP was used, which is suitable for ChIP-seq data (v5.2.0),^79^^,^^78^ for the CTCF mark to determine consensus motifs at the CTCF peak regions. A 500 bp region around the mid-position of CTCF called peaks is used for identification of motifs with MEME-ChIP (-norand, -ccut 0, -meme-nmotifs 30, -meme-minw 8 -meme-maxw 13) and MEME (-nmotifs 10 -minw 8 -maxw 12). Homer software (v4.1.0)^80^ was used for gene, promoter, and transcription factor binding site (TFBS) discovery (-size 300 -len 8,10,13 -mset vertebrates), as well as annotating peak regions for the histone and CTCF marks. Regions showing gene silencing, promoters and enhancers were visualized using DROMPA.^81^ Lastly, enhancers were identified as H3K27ac peaks which are not within 1000 bp of H3K4me3 peaks.^94^ Read coverage and signal value of peaks, for respective histone marks H3K4me3, H3K4me1, H3K27ac of each experiment are compared using bedtools (v2.30.0)^82^ and plot correlation.

#### ATAC-sequencing analysis

Trimming and alignment of the ATAC-seq reads were completed as described above for ChIP-seq reads. PCR duplicates were removed using picard (v2.23.9), and only unique, paired-end reads were kept for further analysis.^83^ Further filtering included removing the mitochondrial (MT) data, as a method of reducing bias in the results. Reads were shifted +4 bp and −5 bp for positive and negative strands respectively using an in-house unix script^86^ and this was done to account for the 9 bp duplication that occurs due to DNA repair of Tn5 transposase nick.^95^ This shift ensures accurate regions of the chromatin state for TF-footprint and motif related analysis. MACS2 (v2.7.1)^,^^73^^,^^74^ was used for peak calling using default parameters. Peak annotation (homer v4.1.0) and motif analysis^78^ for the peak regions were obtained, and identified motifs were scanned for known motifs such as TFBS and TATA-box using the Homer tool.^80^

#### Methylation analysis

RRBS and WGBS raw reads were trimmed as described above for ChIP-seq reads, with an additional --rrbs parameter for RRBS data. Genome index was built using BSseeker2 v 2.1.8,^96^ with bowtie2 as aligner, for RRBS and WGBS genome, with additional parameters for RRBS: -r -l 10 -u 280. Thereafter the reads were aligned using BSseeker2 (v 2.1.8), with additional parameters for RRBS: --rrbs, -c MspI, -L 10 -U 280 -m 4 and for WGBS: -I 0 -X 1000 -m 4. BSSeeker2 was used for the alignment as this tool is tailored for RRBS as it ‘builds’ a custom reference based on the restriction enzymes cutting sites. It is also more suitable to align gapped-reads than other tools commonly used for methylation analysis.^96^ We decided to keep methylation analysis standard across assays and thus implemented BSSeeker2 for WGBS data as well. The aligned reads of the biological replicates of the pig cell line were merged for further analysis, after a Pearson correlation^97^ showed a high correlation of 0.96 between the two RRBS technical replicate samples (Figure S2). CGmaptools (v0.1.2)^84^ was implemented for the methylation calling from the aligned reads. Further statistical analysis of the methylation data was completed using CGmaptools (v0.1.2) and MethylKit (v1.16.1).^85^ Correlations and clustering between the biological replicates were analyzed using MethylKit.

Finally, the number of sites identified by RRBS data and not by WGBS data was investigated using the following approach. Firstly, the methylation calls were filtered for only CG sites and for a coverage of more than 10. Thereafter bedtools (v2.30.0)^82^ was used to identify the sites unique to RRBS data, and for merging these regions. Functional importance of these regions was investigated by overlaying regions with enhancer and promoter regions detected from the ChIP-seq analysis, visual examination of the sites using Jbrowse,^92^ and motif discovery using homer.

#### Integrative analysis

An integrative approach was used to investigate the relationship between WGS, expression data (RNA-seq), methylation status and ChIP-seq marks. Output files from the homer annotate called peaks for the respective marks, gene expression values from RSEM, and methylation calls from CGmaptools were used. For this investigation, correlations, scatterplots, and boxplots were created from these files using an in-house python script.^86^

### Quantification and statistical analysis

Pearson correlations between the read coverage and signal value of peaks for histone marks H3K4me1, H3K4me3 and H3K27ac of each experiment was done using bedtools (v2.30.0),^82^ statistical function (scipy.stats) and seaborn package for visualisation in python. A pearson correlation was performed between two technical replicates of the pig IPECJ2 cell line, we used the tool MethylKit (v1.16.1)^85^ for this quantification. A high correlation of 0.96 (Figure S2) confirmed that the two samples could be merged for further downstream analysis. Integrative analysis used heatmaps, Pearson correlations, scatterplots and boxplots to investigate the relationships between gene expression, various histone modifications indicating promoters, enhancers and gene silencing (H3K4me1, H3K4me3, H3K27ac, H3K27me3), TF (CTCF) and open chromatin (ATAC-seq). This was done using an in-house python script which is available.^86^

## Supplemental information

Document S1. Figures S1–S21 and Tables S1–S4

Data S1. Complete homer output for identified motifs in Pig IPECJ-2, related to Table 2 Homer motif analysis results for histone modifications H3K4me1, H3K4me3, H3K27ac, and enhancer elements of pig IPECJ2 cell line. P-values >1e-10 are possible false positives. Within each folder (e.g. peak_files_CTCF) are the html files showing the identified motifs when using homer (e.g. homerResults.html).

Data S2. Complete homer output for identified motifs in Chicken SL-29, related to Tables 5 and 6 Homer motif analysis results for histone modifications H3K4me1, H3K4me3, H3K27ac, enhancers, and ATAC-seq of chicken SL-29 cell line. Parameters for possible false positives is as mentioned earlier for S5.

Figure360. An Author Presentation of Figure 1
