# Supplementary material for: Detailed molecular and epigenetic characterization of the pig IPEC-J2 and chicken SL-29 cell lines
Source: iScience. 2023 Feb 20;26(3):106252. doi: 10.1016/j.isci.2023.106252 (PMC10018572; doi:10.1016/j.isci.2023.106252)
Supplement: Document S1. Figures S1–S21 and Tables S1–S4 [file mmc1.pdf]

iScience, Volume *26*

Supplemental information

Detailed molecular and epigenetic characterization of the pig IPEC-J2 and chicken SL-29 cell lines

Jani de Vos, Richard P.M.A. Crooijmans, Martijn F.L. Derks, Susan L. Kloet, Bert Dibbits, Martien A.M. Groenen, and Ole Madsen

Supplementary material

Supplementary tables

Table S1 Summary statistics of the pig IPECJ2 data, related to STAR methods. Table of reads for each of the respective data types and analysis in pig IPECJ2-cell line, with high coverage observed for all data types

<table>
<tr><th colspan="2">Data type</th><th>Raw reads</th><th>Reads after qc</th><th colspan="2">Coverage (x)</th></tr>
<tr><td colspan="2">WGS</td><td>754 708 514</td><td>732 556 460</td><td colspan="2">32</td></tr>
<tr><td colspan="2">RNA-seq</td><td>82 583 468</td><td>81 053 264</td><td colspan="2">10</td></tr>
<tr><td rowspan="6">ChIP-seq</td><td>H3K4me1</td><td>27 080 156</td><td>27 080 156</td><td rowspan="6" colspan="2">NA</td></tr>
<tr><td>H3K4me3</td><td>18 423 318</td><td>18 423 318</td></tr>
<tr><td>H3K27me3</td><td>26 873 342</td><td>26 873 342</td></tr>
<tr><td>H3K27ac</td><td>24 715 848</td><td>25 285 609</td></tr>
<tr><td>CTCF</td><td>34 767 672</td><td>34 767 672</td></tr>
<tr><td>Input</td><td>18 509 577</td><td>18 509 577</td></tr>
<tr><td rowspan="2">RRBS</td><td>Replicate 1 (268)</td><td>20 331 892</td><td>20 046 713</td><td>13</td><td rowspan="2">Merged:
27</td></tr>
<tr><td>Replicate 2 (269)</td><td>23 193 397</td><td>22 873 497</td><td>14.5</td></tr>
<tr><td colspan="2">WGBS</td><td>448 871 868
897 743 736</td><td>1 135 147 598
567 573 799</td><td colspan="2">56</td></tr>
</table>

Table S2 Summary statistics of the chicken SL-29 data, Related to STAR methods. Table of reads for each of the respective data types and analysis in the chicken SL-29 cell line, with ATAC-seq having a lower coverage (<10) in comparison to other data types.

<table>
<tr><th colspan="2">Data type</th><th>Raw reads</th><th>Reads after qc</th><th>Coverage</th></tr>
<tr><td colspan="2">WGS</td><td>241 479 778</td><td>234 225 956</td><td>29</td></tr>
<tr><td colspan="2">RNA-seq</td><td>100 952 798</td><td>99 764 594</td><td>28</td></tr>
<tr><td rowspan="6">ChIP-seq</td><td>H3K4me1</td><td>39 768 244</td><td>36 204 354</td><td rowspan="6">NA</td></tr>
<tr><td>H3K4me3</td><td>54 441 620</td><td>51 732 192</td></tr>
<tr><td>H3K27me3</td><td>57 760 552</td><td>53 134 788</td></tr>
<tr><td>H3K27ac</td><td>43 878 912</td><td>42 046 042</td></tr>
<tr><td>CTCF</td><td>92 857 398</td><td>86 075 418</td></tr>
<tr><td>Input</td><td>44 311 032</td><td>41 957 422</td></tr>
<tr><td colspan="2">RRBS</td><td>20 177 168</td><td>19 896 944</td><td>44</td></tr>
<tr><td colspan="2">WGBS</td><td>244 657 599</td><td>488 310 064</td><td>55</td></tr>
<tr><td colspan="2">ATAC-seq</td><td>193 807 842</td><td>146 216 140</td><td>9</td></tr>
</table>

Table S3 Expression values of onco-genes in jejenum tissue, organoid and cell lines, related to figure 3. TPM expression values of the 10 highly expressed onco-genes are shown, in jejenum tissue (5weeks old), jejenum organoids (3 and 12 weeks) and IPECJ cell line grown for a longer time in comparison to IPECJ2 cell line investigated in this study.

Gene expression (TPM)						
**Onco-genes**	**IPECJ2**	**Tissue**	**Organoid 12w**	**Organoid 3w**	**IPECJ91**	**IPECJ87**
CTNNB1	445.99	245.98	284.51	235.57	673.06	687.05
HMGA1	405.51	150.04	711.84	677.82	242.11	275.64
CCND1	300.48	24.69	40.62	32.53	252.08	208.39
MET	281.4	3.69	25.39	28.25	133.23	135.68
AKT1	232.17	172.04	185.71	144.82	132.62	125.85
EWSR1	211.86	203.87	269.14	222.11	288.82	271.69
FUS	198.97	143.22	260.12	230.62	166.15	131
DEK	182.34	86.37	213.85	205.02	162.92	156.25
PTPN11	174.74	34.78	75.73	79.59	199.25	194.06
CCDN2	153.74	10.73	39.48	24.59	51.51	43.28

Table S4 Motif enrichment of unique RRBS sites not covered by WGBS in enhancer regions, related to figure 11. Top three enriched known binding motifs identified from consensus peaks are shown in this table.

Motif	Transcription factor	% of target regions	P-value
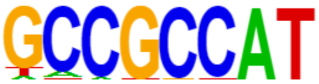	YY2	28.06%	1e-55
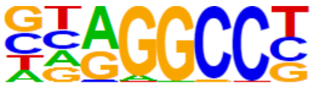	ZNF711(Zf)	65.52%	1e-47
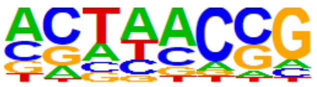	BMYB(HTH)	54.92%	1e-42

Supplementary figures

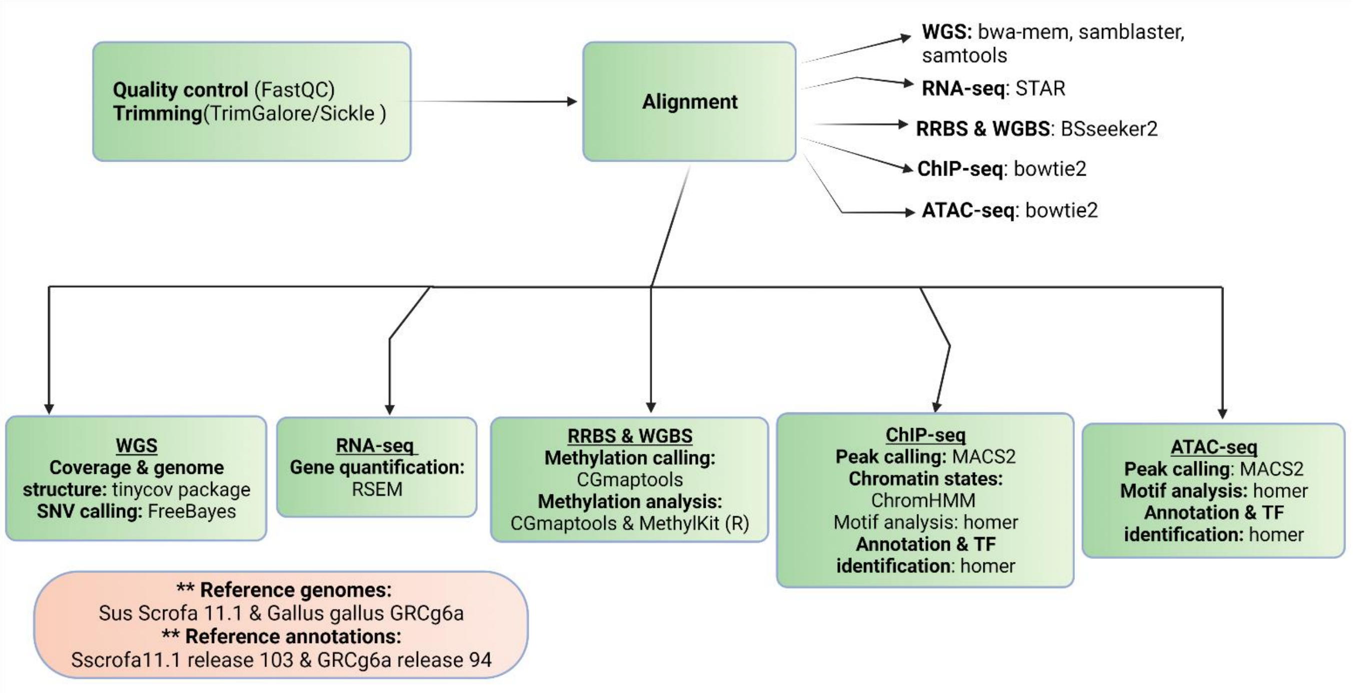

Figure S1 Schematic of methodology followed for all data analysis in brief, related to STAR methods. This shows reference genomes used for all analysis and indexing, tools implemented for QC, trimming and alignment as well as for downstream analysis.

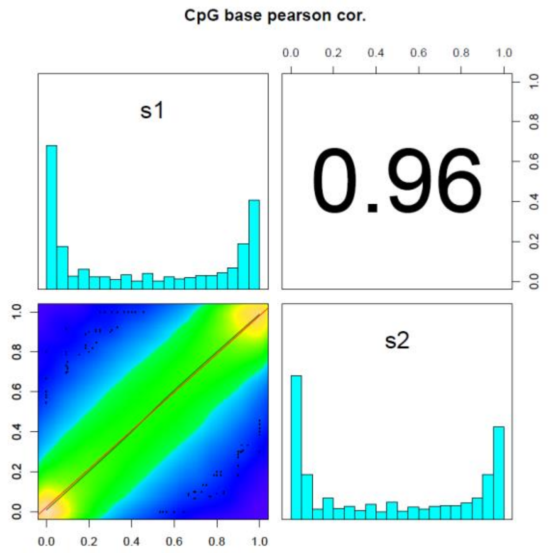

Figure S2 Correlation between two RRBS technical replicates, related to STAR methods. This shows a high correlation as expected, which confirms merging of the replicates for further downstream analysis.

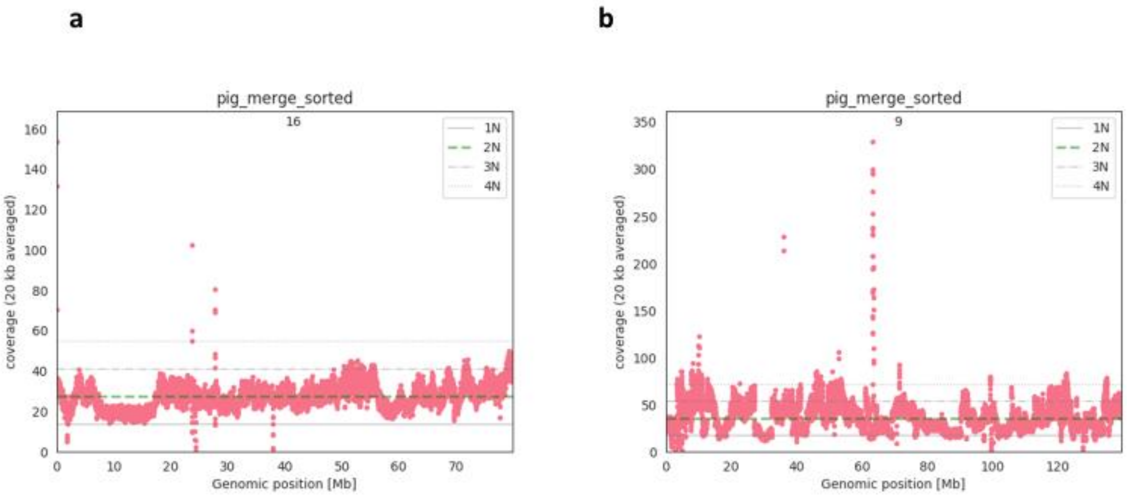

Figure S3 Coverage plots of individual chromosomes, related to figure 2. These plots show chromosomes a. 16, from which a deletion can be observed from ~9Mb-19Mb and b. 9, which shows dispersed read coverage over the chromosome, with very high coverage in some positions.

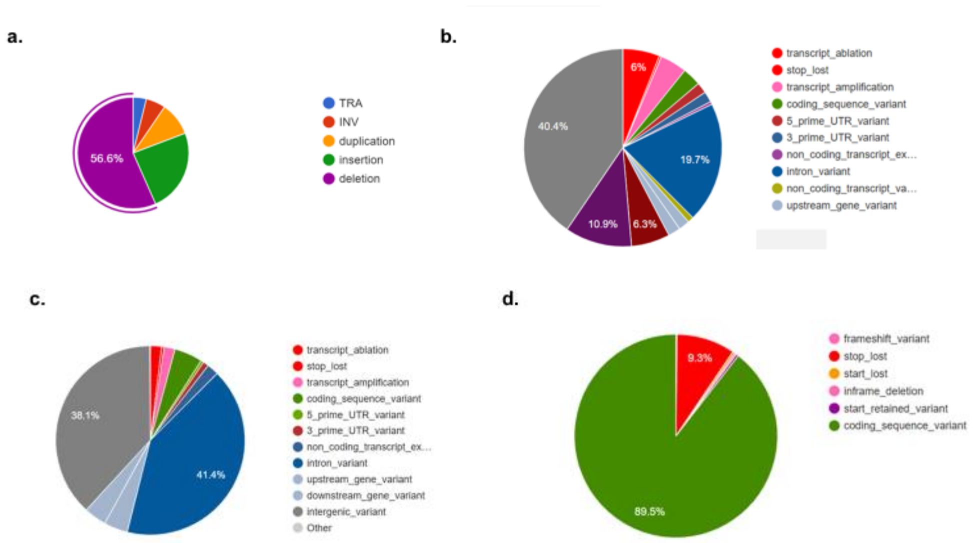

Figure S4 Variant effect prediction from Manta output, related to figure 2. These effects are estimated by VEP tool from the SV calls and shows a. number of each SV; b. consequences occurring from the identified SV's; c. most severe consequences identified; d. coding consequences that occur.

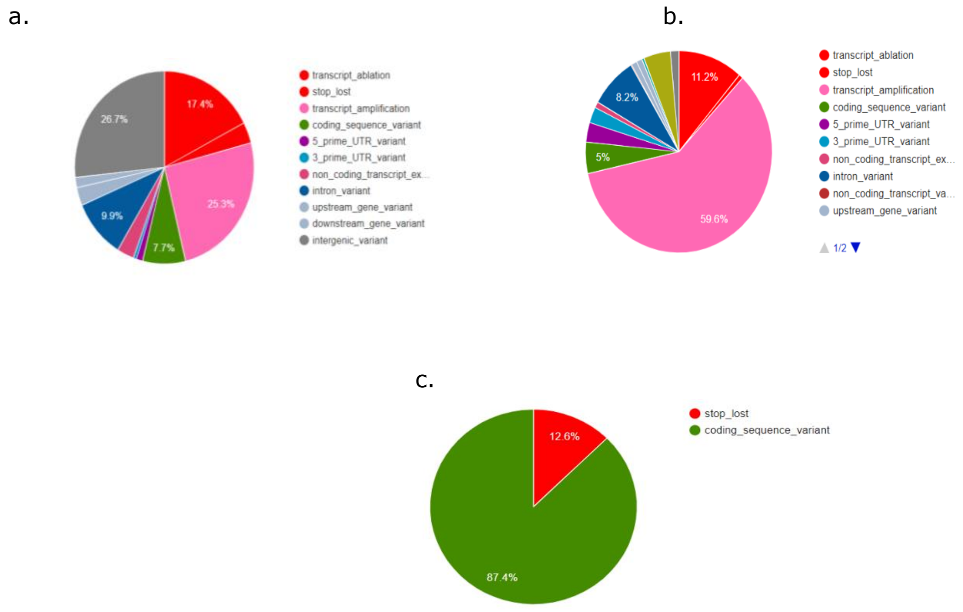

Figure S5 Variant effect prediction (VEP tool) from the SV calls from CNVnator output, related to figure 2. This shows a. most severe consequences identified; b. consequences occurring from the identified SV's; c. coding consequences that occur.

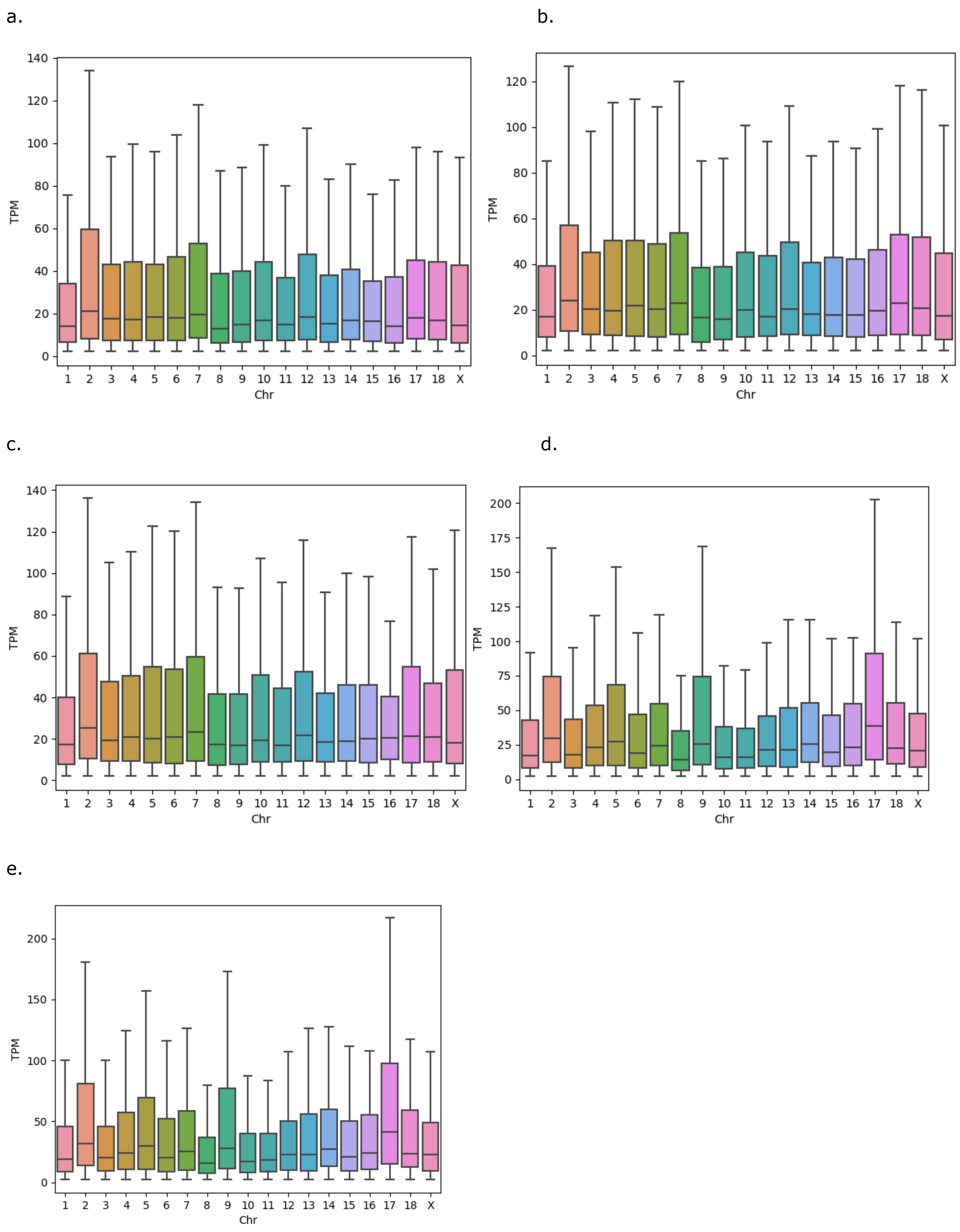

Figure S6 Expression values of genes per chromosome, related to figure 3. Genes with a TPM > 2 has been plotted here which removes genes with very low/ no expression. a. 5 Week old jejenum tissue sample; b. Organoid sample grown for 3 weeks; c. Organoid sample grown for 12 weeks, d. IPECJ87 Cell line grown for 87 passages, e. IPECJ91 Cell line grown for 91 passages.

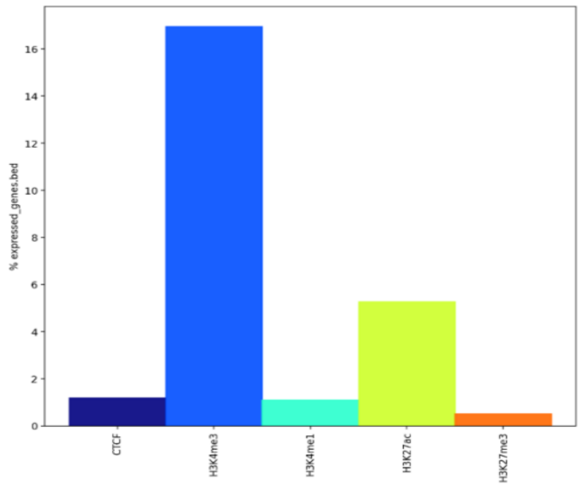

Figure S7 ChIP-seq marks enriched around TSS of expressed genes (+/- 1000bp), related to table 1. H3K4me3 shows an enrichment around 17.5% of the TSS of expressed genes.

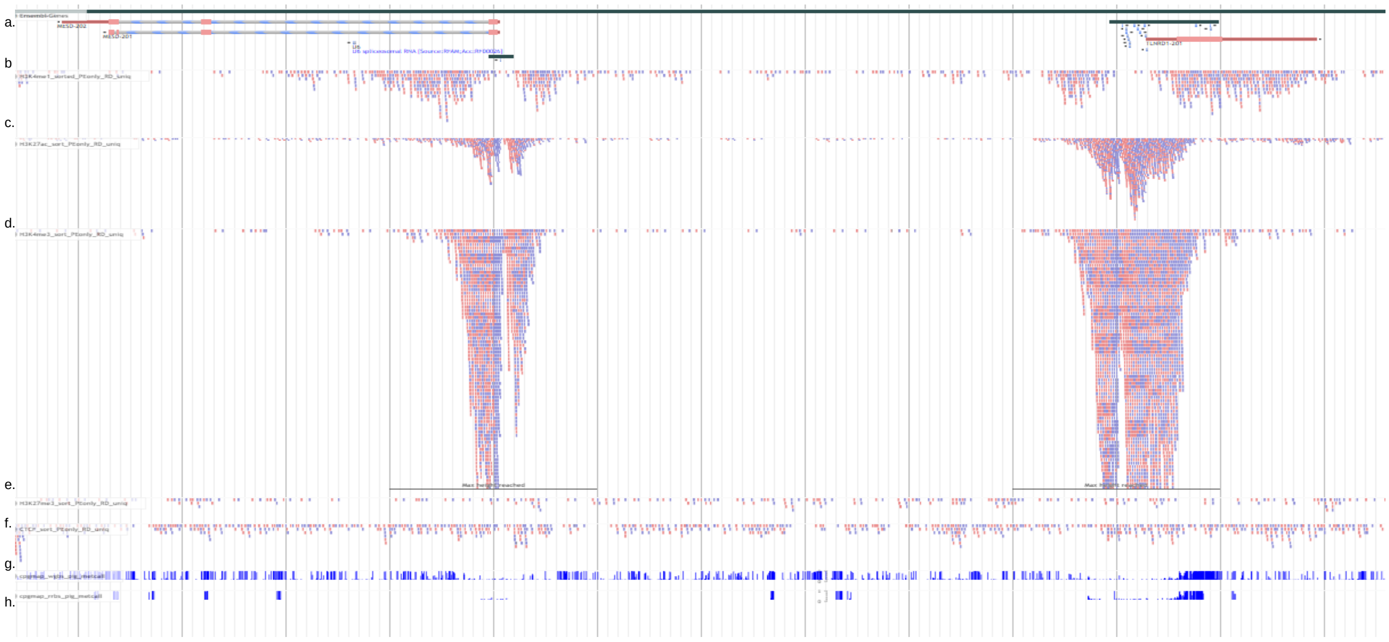

Figure S8 Genome browser view of various histone modifications, and methylation in pig IPECJ-2, related to figure 4. The aligned reads of individual histone modifications, CTCF, methylated sites (RRBS and WGBS) in the pig IPECJ2 cell line for MESD genes (involved in mesodermal development) on chromosome 7 are shown. (a. genes, b. H3K4me1, c. H3K27ac, d. H3K4me3, e. H3K27me3, f. CTCF, g WGBS, h. RRBS).

a. b. c. d.

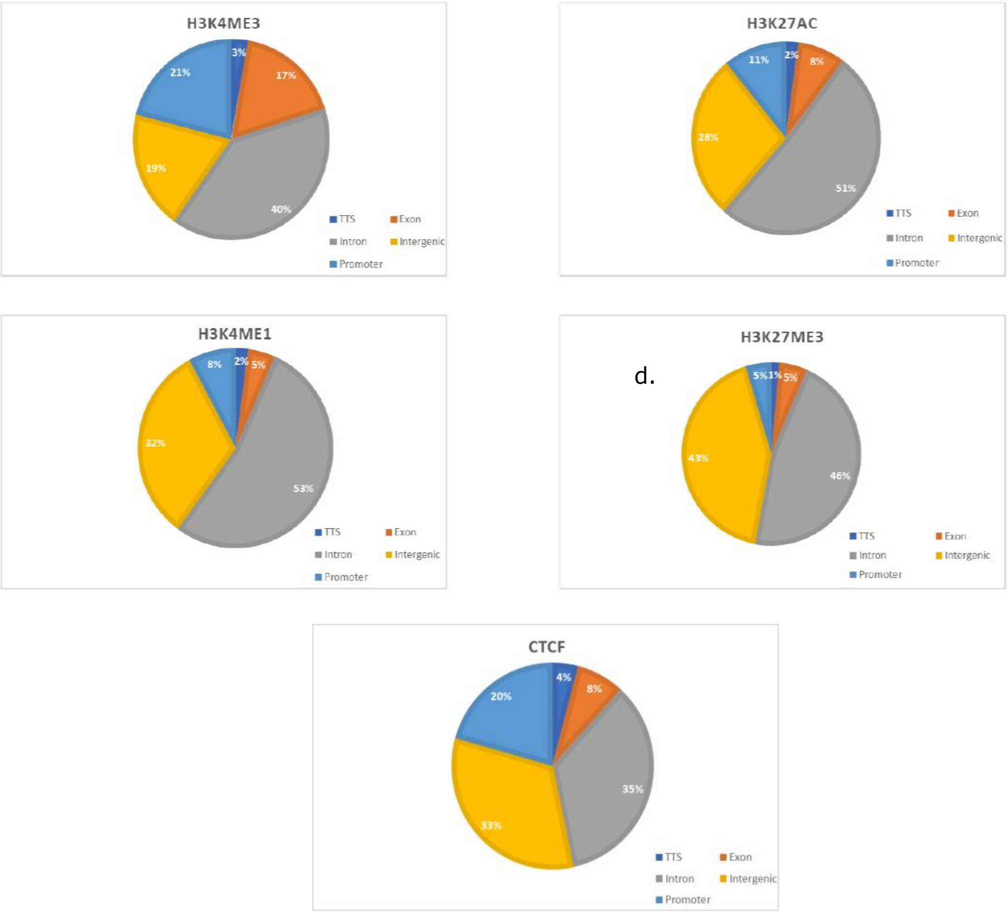

Figure S9 Distribution of histone modification peaks in different genomic regions, related to figure 4. Five genomic regions including promoter, TTS, intron, exon, and intergenic were classified with homer for a. H3K4me3, b. H3K27ac, c. H3K4me1, d. H3K27me3 and e. CTCF.

a.

b.

c.

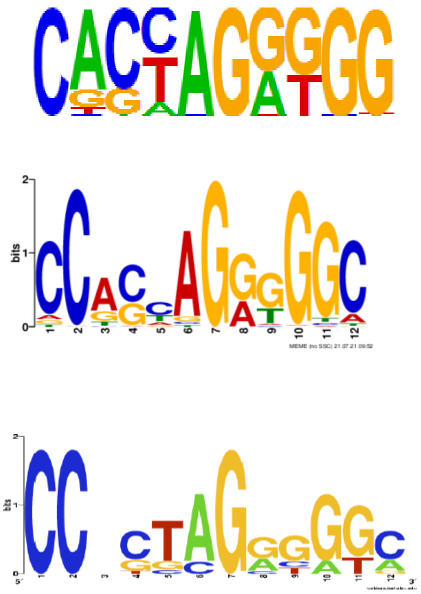

Figure S10 Consensus sequences of pig CTCF, related to table 2. These were generated using a. homer motif analysis. b. memeSuite motif analysis. and c. from the human CTCF consensus sequence.

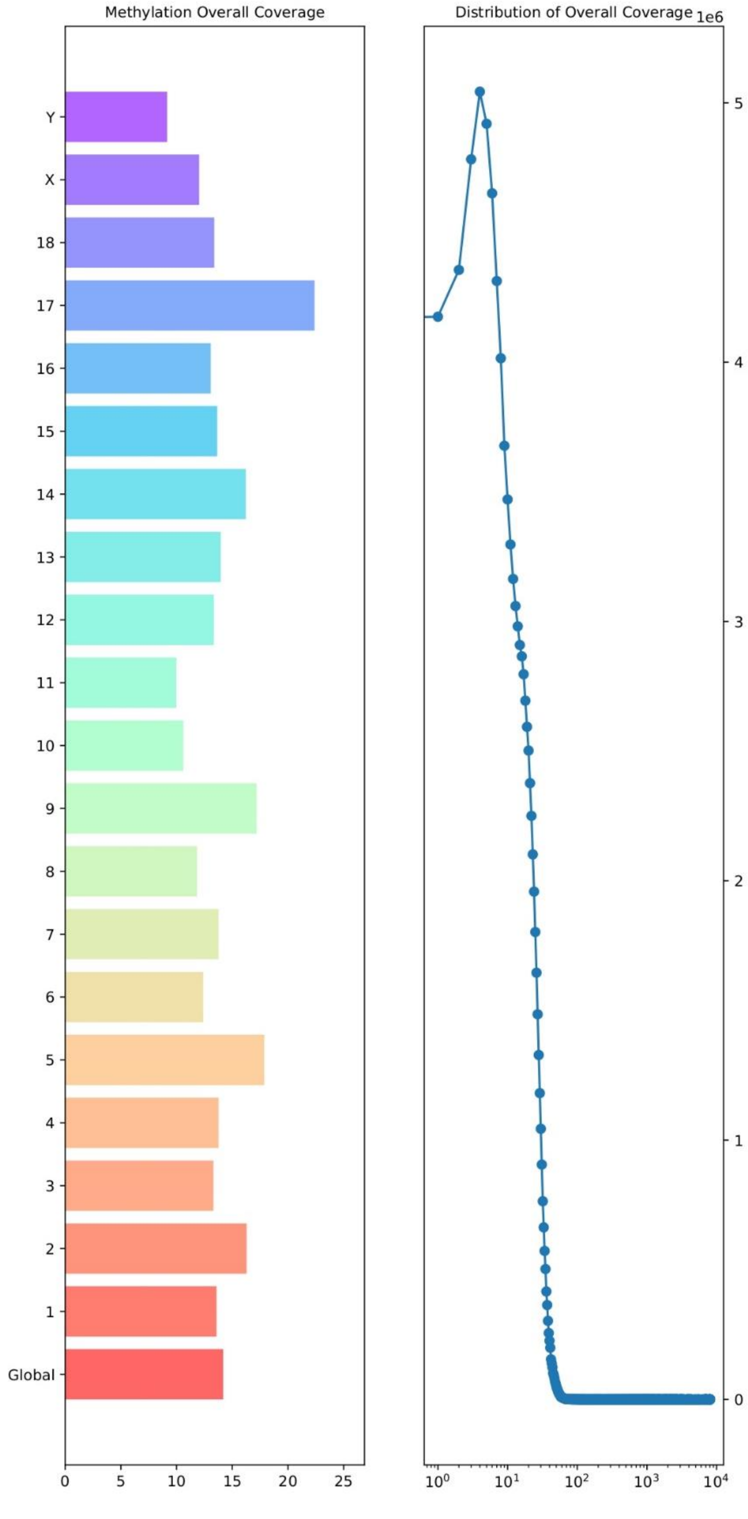

Figure S11 Coverage statistics of the pig IPECJ-2 methylome, related to figure 1 and STAR methods. Methylation effective coverage of pig WGBS data displayed per chromosome as shown in the left panel, is calculated as the average read coverage only for cytosines. Distribution of the overall coverage calculated for each chromosome (right), which is calculated as the average read coverage on all nucleotides on both strands.

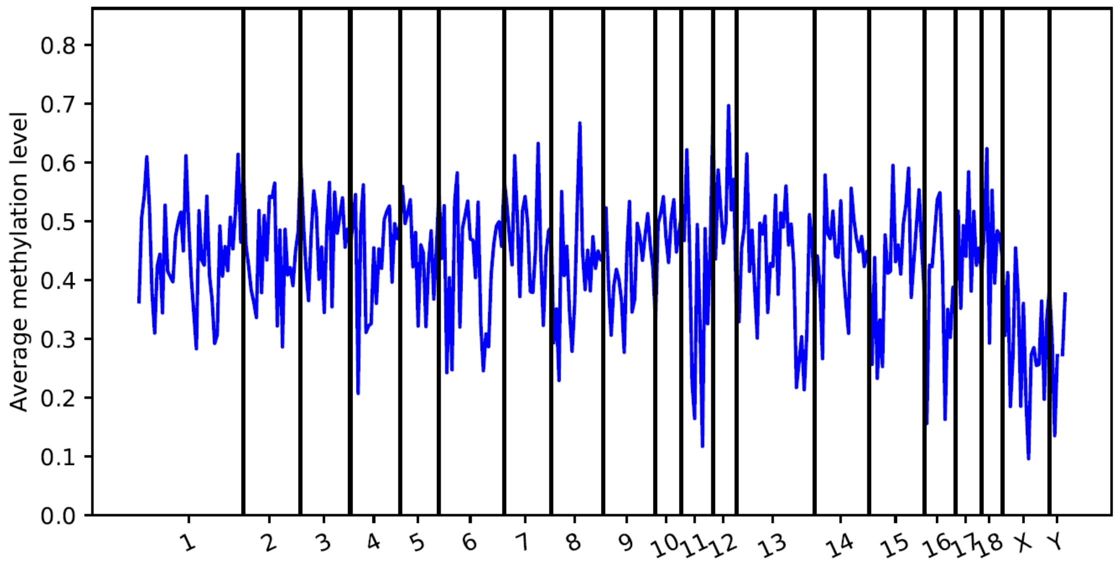

Figure S12 Average methylation levels across the genome, related to table 3. This is shown in equal-length bins (5000000bp/bin) for pig WGBS data.

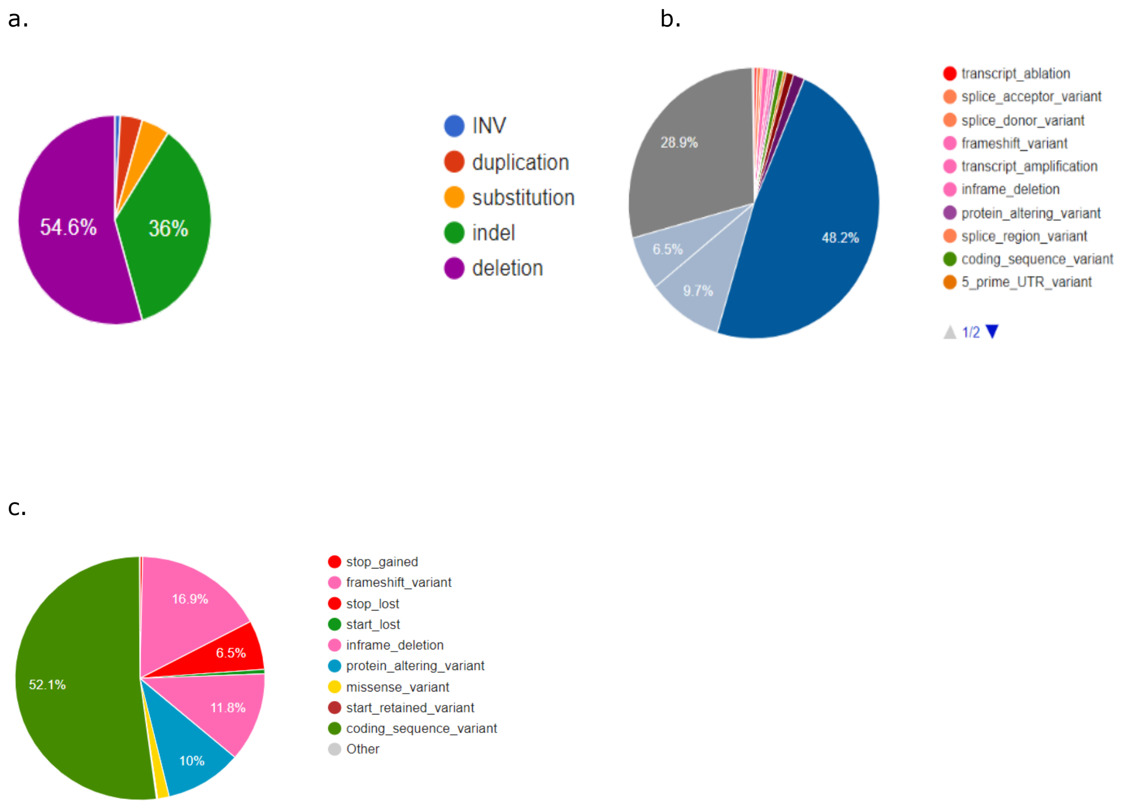

Figure S13 Variant effect prediction from SV calls, related to figure 8. These effects are estimated by VEP tool using the outputs from Manta with a. Distribution of large structural variants over the whole genome in chicken SL-29 cell line, detected by Manta software. b showing most severe variant effects of the structural variants detected by Manta and c. coding consequences of the variants. Variant effects as shown by VEP tool.

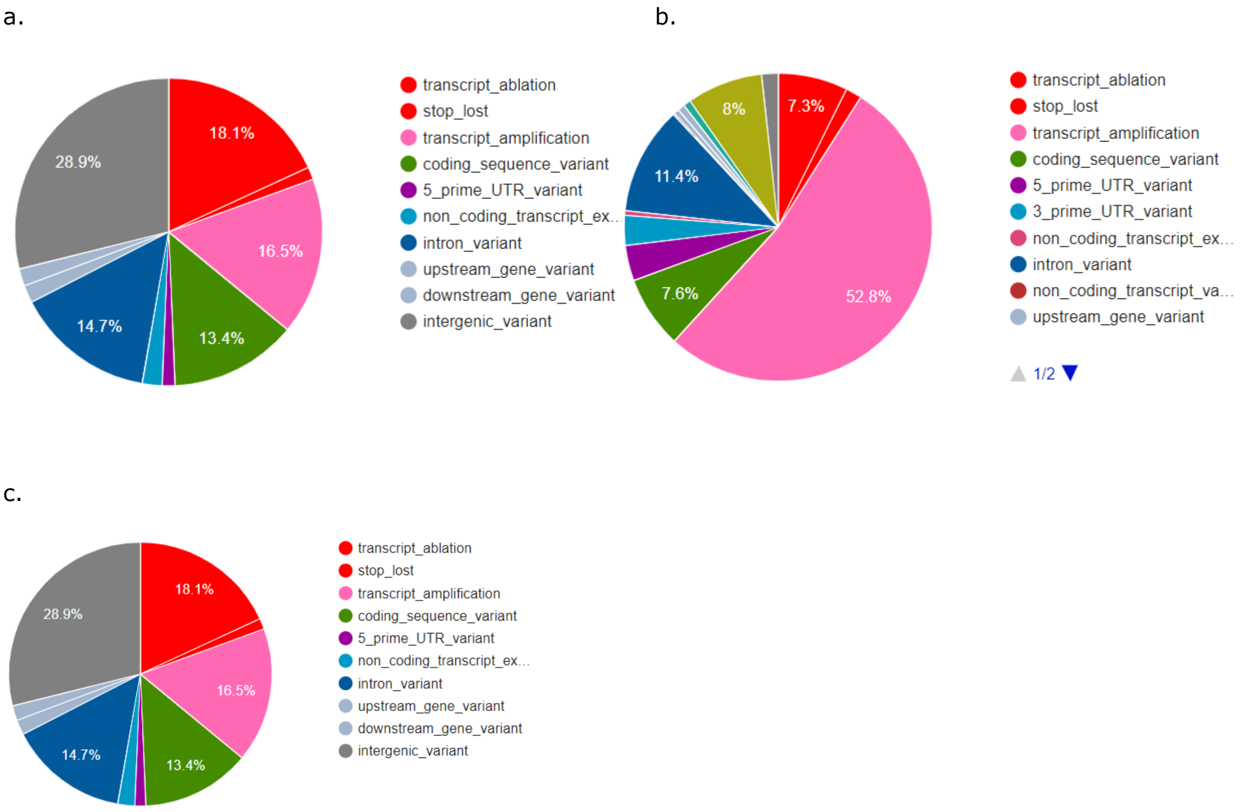

Figure S14 Variant effect prediction from the SV calls (CNVnator output), related to figure 8. Effect predictions showing a. most severe consequences identified; b. consequences occurring from the identified SV's; c. coding consequences that occur.

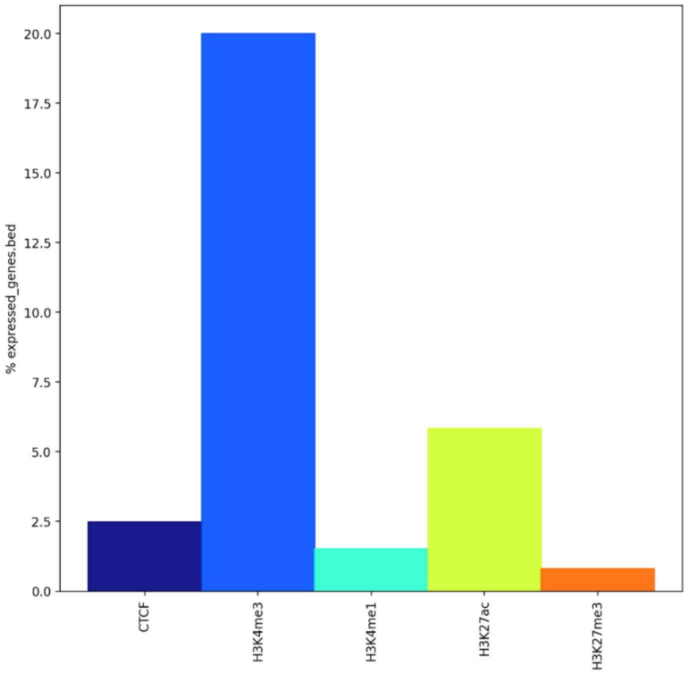

Figure S15 Enrichment of ChIP-seq marks around 1000 bp of expressed genes TSS, related to figure 10. Histone mark H3K4me3 is enriched around 20% of TSS of enriched genes.

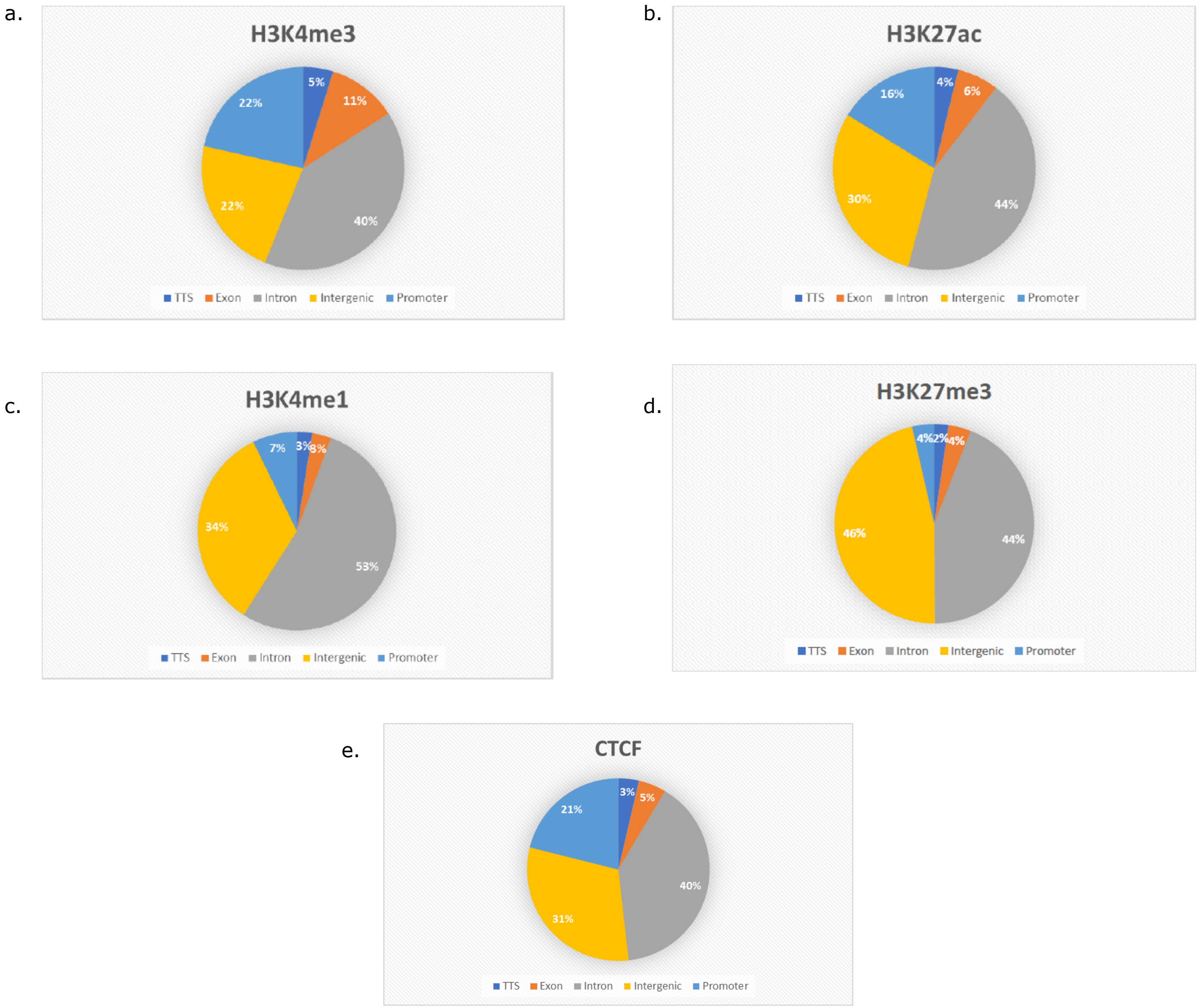

Figure S16 Distribution of histone modification peaks in different genomic regions, related to figure 10. Five genomic regions including promoter, TTS, intron, exon, and intergenic were classified with homer for a. H3K4me3, b. H3K27ac, c. H3K4me1, d. H3K27me3 and e. CTCF.

a.

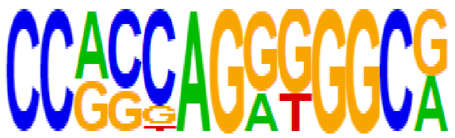

b.

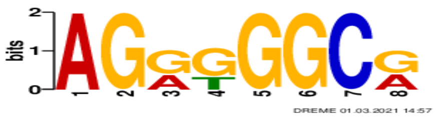

c.

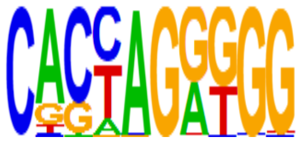

Figure S17 Consensus sequences of chicken CTCF, related to figure 10. Consensus sequences are obtained from a. homer motif analysis. b. MEME-suite c. from the human CTCF consensus sequence.

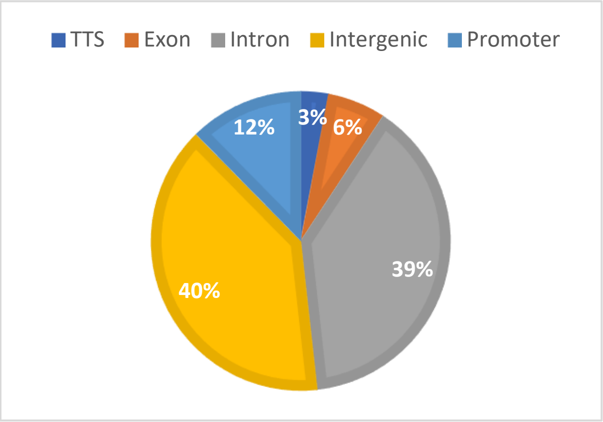

Figure S18 Distribution of ATAC-seq peaks in different genomic regions, related to table 6. Five genomic regions including promoter, TTS, intron, exon, and intergenic were classified with homer.

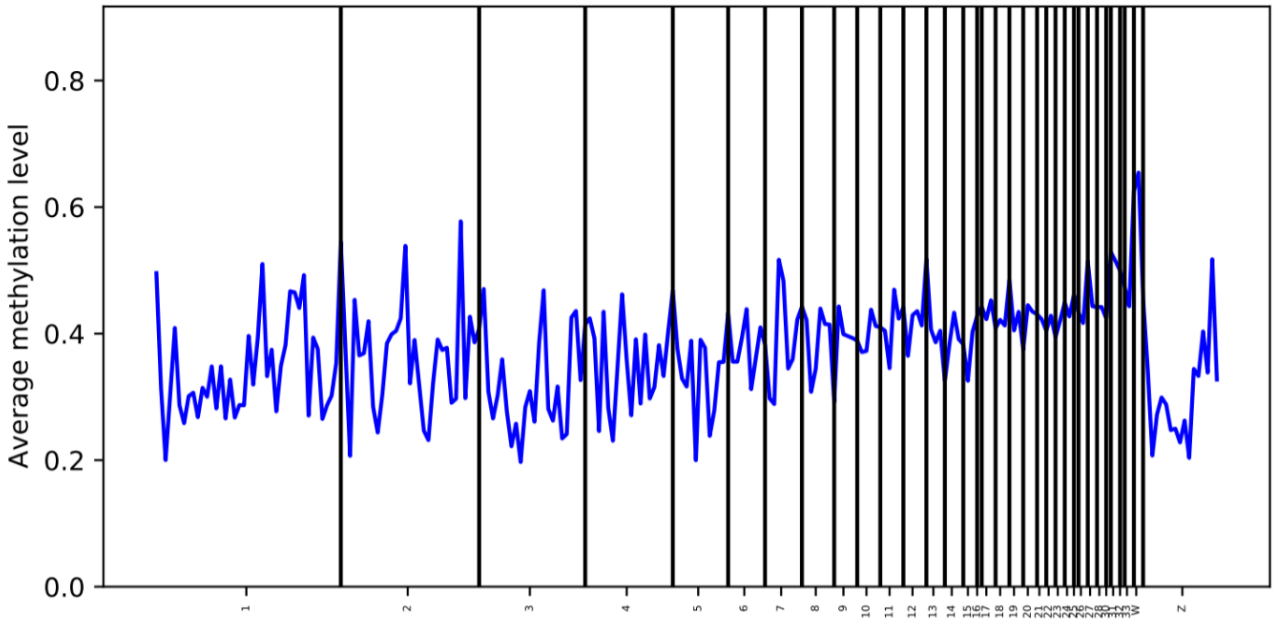

Figure S19 Average methylation levels in equal-length bins for chicken WGBS, across the genome, related to table 7.

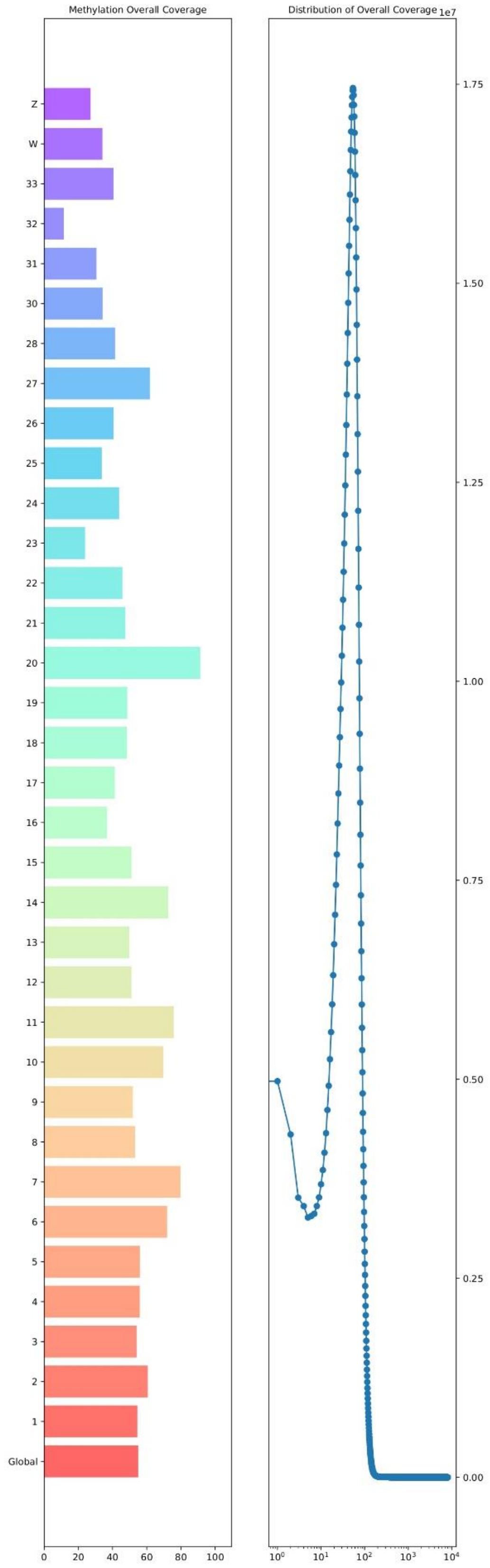

Figure S20 Methylation coverage per chromosome, related to figure 7 and STAR Methods. Methylation effective coverage of chicken WGBS displayed per chromosome (left) and distribution of the overall coverage calculated for each chromosome (right).

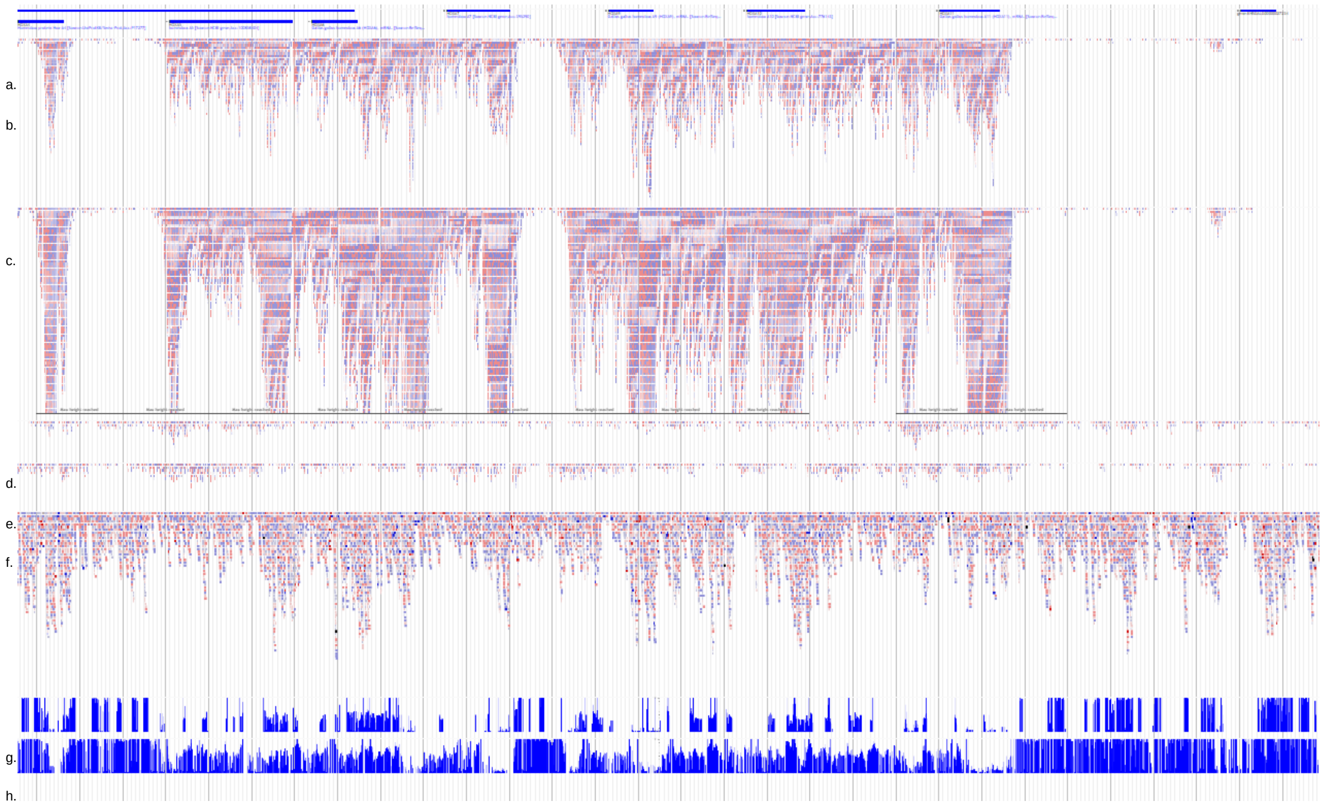

Figure S21 Visualisation of individual histone modifications, methylation and ATAC-seq in chicken SL-29, related to figure 12 and figure 13. This is shown for HOX genes on chromosome 2. (a. genes, b. H3K4me3, c. H3K27ac, d. H3K4me1, e. H3K27me3, f. ATAC-seq, g. RRBS, h. WGBS).
