# Supplementary material for: Detailed molecular and epigenetic characterization of the pig IPEC-J2 and chicken SL-29 cell lines
Source: iScience. 2023 Feb 20;26(3):106252. doi: 10.1016/j.isci.2023.106252 (PMC10018572; doi:10.1016/j.isci.2023.106252)
Supplement: Data S1. Complete homer output for identified motifs in Pig IPECJ-2, related to Table 2 — Homer motif analysis results for histone modifications H3K4me1, H3K4me3, H3K27ac, and enhancer elements of pig IPECJ2 cell line. P-values >1e-10 are possible false positives. Within each folder (e.g. peak_files_CTCF) are the html files showing the identified motifs when using homer (e.g. homerResults.html). [file mmc2.zip › S5/Pig_IPECJ_2/peak_fileS_CTCF/homerResults.html]

peak\_files/ - Homer de novo Motif Results


# Homer *de novo* Motif Results (peak\_files/)

Known Motif Enrichment Results  
Gene Ontology Enrichment Results  
If Homer is having trouble matching a motif to a known motif, try copy/pasting the matrix file into
STAMP  
More information on motif finding results: HOMER
| Description of Results
| Tips
  
Total target sequences = 4682  
Total background sequences = 42295  
\* - possible false positive  

|  |  |  |  |  |  |  |  |  |
| --- | --- | --- | --- | --- | --- | --- | --- | --- |
| Rank | Motif | P-value | log P-pvalue | % of Targets | % of Background | STD(Bg STD) | Best Match/Details | Motif File |
| 1 | G T A C A G T C C T G A T A G C G A T C C T G A A C T G C T A G C A T G A C T G A C T G G A T C T C A G | 1e-3157 | -7.270e+03 | 58.93% | 2.32% | 45.7bp (95.4bp) | BORIS(Zf)/K562-CTCFL-ChIP-Seq(GSE32465)/Homer(0.954) More Information | Similar Motifs Found | motif file (matrix) |
| 2 | C T A G A C T G A G T C T A C G A T G C G A C T T G C A A G C T | 1e-249 | -5.748e+02 | 56.49% | 32.44% | 74.2bp (103.5bp) | PB0112.1\_E2F2\_2/Jaspar(0.726) More Information | Similar Motifs Found | motif file (matrix) |
| 3 | A C T G C T G A C A G T A T G C C T A G A T G C C T G A T A C G G A C T C T G A | 1e-244 | -5.634e+02 | 15.40% | 3.43% | 67.4bp (88.9bp) | HIC1(Zf)/Treg-ZBTB29-ChIP-Seq(GSE99889)/Homer(0.607) More Information | Similar Motifs Found | motif file (matrix) |
| 4 | G C T A A T C G C T A G A C G T C A T G A C T G A G T C C T A G A T G C G C A T T A C G A G C T A G C T | 1e-223 | -5.144e+02 | 6.64% | 0.53% | 67.5bp (96.4bp) | PB0151.1\_Myf6\_2/Jaspar(0.694) More Information | Similar Motifs Found | motif file (matrix) |
| 5 | G A T C C T G A T A G C G C A T A C T G T A G C G T C A A C G T C A G T A T C G G C T A T C G A C A G T | 1e-73 | -1.694e+02 | 0.75% | 0.00% | 84.3bp (48.6bp) | PB0091.1\_Zbtb3\_1/Jaspar(0.676) More Information | Similar Motifs Found | motif file (matrix) |
| 6 | C G T A C T A G A C G T A C G T A C T G A G T C C G A T A C T G A C G T A C G T C G T A C T A G C T G A | 1e-58 | -1.353e+02 | 0.62% | 0.00% | 61.3bp (75.3bp) | Rhox11/MA0629.1/Jaspar(0.606) More Information | Similar Motifs Found | motif file (matrix) |
| 7 | C G T A A C G T G A T C C T A G A C T G C A T G C G T A A G C T A T C G A T G C C G T A G C A T A C G T | 1e-54 | -1.256e+02 | 0.77% | 0.01% | 78.7bp (97.8bp) | PB0077.1\_Spdef\_1/Jaspar(0.662) More Information | Similar Motifs Found | motif file (matrix) |
| 8 | C A T G G T A C C T G A T A C G G C A T C A G T A T G C G A T C A T C G T A G C | 1e-52 | -1.210e+02 | 20.40% | 12.43% | 78.0bp (97.0bp) | ETV6/MA0645.1/Jaspar(0.778) More Information | Similar Motifs Found | motif file (matrix) |
| 9 | A C G T A G C T A C G T A C G T A C G T A C G T A C G T A G C T C G A T A C G T A C G T C G T A G C T A | 1e-43 | -9.941e+01 | 15.59% | 9.22% | 54.4bp (122.6bp) | PB0182.1\_Srf\_2/Jaspar(0.907) More Information | Similar Motifs Found | motif file (matrix) |
| 10 | A G T C C A T G C A G T C G A T G T C A A C T G T G A C C T A G | 1e-43 | -9.914e+01 | 26.42% | 18.22% | 84.6bp (96.2bp) | OSR1/MA1542.1/Jaspar(0.676) More Information | Similar Motifs Found | motif file (matrix) |
| 11 | T C A G A G C T A T C G T A C G G T A C G T A C G T C A T C G A | 1e-42 | -9.872e+01 | 48.61% | 38.66% | 80.8bp (91.0bp) | Bcl11a(Zf)/HSPC-BCL11A-ChIP-Seq(GSE104676)/Homer(0.751) More Information | Similar Motifs Found | motif file (matrix) |
| 12 | A C T G T C G A C G A T A T C G A G T C C G T A A C G T C A G T A C T G A C T G T C A G A G T C A G T C | 1e-35 | -8.162e+01 | 0.51% | 0.01% | 86.7bp (65.0bp) | DMRT3/MA0610.1/Jaspar(0.582) More Information | Similar Motifs Found | motif file (matrix) |
| 13 | A C T G C T A G G A C T C G T A T A G C G C A T T G A C G C A T | 1e-33 | -7.773e+01 | 42.78% | 34.18% | 85.8bp (89.9bp) | Nkx2.5(Homeobox)/HL1-Nkx2.5.biotin-ChIP-Seq(GSE21529)/Homer(0.595) More Information | Similar Motifs Found | motif file (matrix) |
| 14 | T G A C G T C A C G A T C A G T A T G C A T G C T C A G T A G C | 1e-31 | -7.320e+01 | 32.76% | 25.05% | 87.5bp (93.8bp) | TEAD4(TEA)/Tropoblast-Tead4-ChIP-Seq(GSE37350)/Homer(0.781) More Information | Similar Motifs Found | motif file (matrix) |
| 15 | C G T A G T C A C T G A G T C A G T C A C T G A G A C T C T A G A T C G T G C A G C T A A G T C T G A C | 1e-28 | -6.474e+01 | 0.43% | 0.01% | 79.8bp (105.6bp) | NFATC4/MA1525.1/Jaspar(0.675) More Information | Similar Motifs Found | motif file (matrix) |
| 16 | T G A C A T C G A C T G G C A T C A G T C A G T A G T C A G T C | 1e-26 | -5.997e+01 | 32.76% | 25.77% | 86.8bp (96.3bp) | PRDM4/MA1647.1/Jaspar(0.738) More Information | Similar Motifs Found | motif file (matrix) |
| 17 | A T C G T G A C C G T A A C T G T C G A T C G A G C A T A C G T A G T C G T A C | 1e-24 | -5.652e+01 | 16.51% | 11.43% | 83.5bp (88.6bp) | TEAD4(TEA)/Tropoblast-Tead4-ChIP-Seq(GSE37350)/Homer(0.664) More Information | Similar Motifs Found | motif file (matrix) |
| 18 | A T G C A G T C A G T C A C G T C G T A C T A G C T A G A C G T | 1e-24 | -5.544e+01 | 20.67% | 15.08% | 89.4bp (90.4bp) | ZNF711(Zf)/SHSY5Y-ZNF711-ChIP-Seq(GSE20673)/Homer(0.633) More Information | Similar Motifs Found | motif file (matrix) |
| 19 | A C T G G T C A A C T G T C G A A C T G G T C A A C T G G T C A | 1e-22 | -5.132e+01 | 27.74% | 21.65% | 82.2bp (99.5bp) | PRDM1/MA0508.3/Jaspar(0.746) More Information | Similar Motifs Found | motif file (matrix) |
| 20 | G T C A C G T A C A G T T A G C G T C A G T A C C T A G A G T C | 1e-20 | -4.809e+01 | 6.04% | 3.29% | 84.3bp (91.6bp) | Ahr::Arnt/MA0006.1/Jaspar(0.765) More Information | Similar Motifs Found | motif file (matrix) |
| 21 | A C T G A G T C A C G T A C G T A C T G A C T G A G T C A G C T A C T G A C T G C G A T A C T G A C T G | 1e-20 | -4.653e+01 | 0.30% | 0.00% | 83.1bp (18.5bp) | Zfp809(Zf)/ES-Zfp809-ChIP-Seq(GSE70799)/Homer(0.643) More Information | Similar Motifs Found | motif file (matrix) |
| 22 | T A C G A G T C C T A G G T A C C G A T C A G T T C A G T A G C A C T G G A C T | 1e-19 | -4.536e+01 | 5.32% | 2.82% | 80.0bp (107.5bp) | NRF1/MA0506.1/Jaspar(0.722) More Information | Similar Motifs Found | motif file (matrix) |
| 23 | C G A T A C G T A G T C G T C A A G T C C G A T A C T G A C T G A T C G A G T C A G C T A G C T C T G A | 1e-18 | -4.183e+01 | 0.23% | 0.00% | 76.0bp (29.6bp) | PB0195.1\_Zbtb3\_2/Jaspar(0.571) More Information | Similar Motifs Found | motif file (matrix) |
| 24 | G T A C A T G C T G C A A T C G T C G A A C T G A T C G T A G C T G A C A T C G T A G C A T G C G A T C | 1e-16 | -3.843e+01 | 0.66% | 0.09% | 76.2bp (94.8bp) | PB0052.1\_Plagl1\_1/Jaspar(0.743) More Information | Similar Motifs Found | motif file (matrix) |
| 25 | C G T A C G A T C G T A A C T G C T A G C G T A A C T G C G T A C G T A A G C T C T A G G T A C A C G T | 1e-16 | -3.723e+01 | 0.21% | 0.00% | 59.6bp (0.0bp) | Sox6/MA0515.1/Jaspar(0.564) More Information | Similar Motifs Found | motif file (matrix) |
| 26 | A G T C C T A G A T C G T G C A C G T A T C A G A G C T C G A T C T G A C A T G | 1e-15 | -3.473e+01 | 5.43% | 3.18% | 86.8bp (91.4bp) | ETV5/MA0765.2/Jaspar(0.809) More Information | Similar Motifs Found | motif file (matrix) |
| 27 | A G C T A C G T A G C T A G T C A C T G C G T A C G T A C G T A A G T C A C T G | 1e-14 | -3.454e+01 | 0.36% | 0.02% | 82.2bp (70.3bp) | PB0034.1\_Irf4\_1/Jaspar(0.741) More Information | Similar Motifs Found | motif file (matrix) |
| 28 | C A T G A T C G T C G A G C A T C A G T C T G A A G T C C T G A | 1e-14 | -3.281e+01 | 6.62% | 4.17% | 86.2bp (88.3bp) | PITX2/MA1547.1/Jaspar(0.787) More Information | Similar Motifs Found | motif file (matrix) |
| 29 \* | C T A G A C G T A C G T A G T C A C G T C T G A A C G T A C G T A G T C A C T G | 1e-2 | -5.927e+00 | 0.38% | 0.18% | 90.8bp (85.2bp) | PB0194.1\_Zbtb12\_2/Jaspar(0.672) More Information | Similar Motifs Found | motif file (matrix) |
