# Supplementary material for: Detailed molecular and epigenetic characterization of the pig IPEC-J2 and chicken SL-29 cell lines
Source: iScience. 2023 Feb 20;26(3):106252. doi: 10.1016/j.isci.2023.106252 (PMC10018572; doi:10.1016/j.isci.2023.106252)
Supplement: Data S1. Complete homer output for identified motifs in Pig IPECJ-2, related to Table 2 — Homer motif analysis results for histone modifications H3K4me1, H3K4me3, H3K27ac, and enhancer elements of pig IPECJ2 cell line. P-values >1e-10 are possible false positives. Within each folder (e.g. peak_files_CTCF) are the html files showing the identified motifs when using homer (e.g. homerResults.html). [file mmc2.zip › S5/Pig_IPECJ_2/peak_fileS_CTCF/homerResults/motif1.similar.html]

motif1

## Information for motif1

G
T
A
C
A
G
T
C
C
T
G
A
T
A
G
C
G
A
T
C
C
T
G
A
A
C
T
G
C
T
A
G
C
A
T
G
A
C
T
G
A
C
T
G
G
A
T
C
T
C
A
G
  
Reverse Opposite:  

A
G
T
C
C
T
A
G
G
T
A
C
A
G
T
C
G
T
A
C
A
G
T
C
A
G
T
C
G
A
C
T
C
T
A
G
A
T
C
G
G
A
C
T
A
C
T
G
C
A
T
G
  

|  |  |
| --- | --- |
| p-value: | 1e-3157 |
| log p-value: | -7.270e+03 |
| Information Content per bp: | 1.721 |
| Number of Target Sequences with motif | 2759.0 |
| Percentage of Target Sequences with motif | 58.93% |
| Number of Background Sequences with motif | 979.5 |
| Percentage of Background Sequences with motif | 2.32% |
| Average Position of motif in Targets | 148.1 +/- 45.7bp |
| Average Position of motif in Background | 157.4 +/- 95.4bp |
| Strand Bias (log2 ratio + to - strand density) | -0.0 |
| Multiplicity (# of sites on avg that occur together) | 1.12 |
| Motif File: | file (matrix) reverse opposite |

### Similar de novo motifs found

|  |  |  |  |  |  |  |  |
| --- | --- | --- | --- | --- | --- | --- | --- |
| Rank | Match Score | Redundant Motif | P-value | log P-value | % of Targets | % of Background | Motif file |
| 1 | 0.892 | T G A C T A G C G T A C G A T C G T A C A C G T C T A G A T C G G A C T A C T G | 1e-2001 | -4608.629323 | 52.71% | 4.34% | motif file (matrix) |
| 2 | 0.889 | A T C G T G A C G A T C T G A C A G T C G A T C G C A T C T G A A T C G G A C T C A T G A T C G G A T C | 1e-1882 | -4333.830994 | 61.70% | 8.00% | motif file (matrix) |
| 3 | 0.860 | A T G C C T G A T A G C G A T C C G T A C T A G C T A G A C T G T C A G T A C G G T A C T C A G T A G C | 1e-1840 | -4238.018971 | 51.99% | 4.86% | motif file (matrix) |
| 4 | 0.844 | A G T C G T A C C T G A A T G C G A C T C T G A A C T G C T A G C A T G A C T G | 1e-1425 | -3281.612097 | 64.25% | 13.19% | motif file (matrix) |
| 5 | 0.693 | G C T A T C A G C T A G A C T G C T A G C A T G G T A C C T A G G A T C G C A T | 1e-941 | -2167.307393 | 34.96% | 4.57% | motif file (matrix) |
| 6 | 0.784 | A C T G A G T C A G T C G T A C A G T C A G T C A C G T C T A G | 1e-882 | -2032.012301 | 52.65% | 13.44% | motif file (matrix) |
| 7 | 0.644 | A T C G A G T C C T A G A T G C A G T C T G A C A G C T G A T C A C G T C G T A | 1e-541 | -1245.862529 | 31.46% | 6.96% | motif file (matrix) |
| 8 | 0.718 | G A T C A T G C A G C T C G T A A T C G G C A T T A C G T A C G | 1e-494 | -1137.993299 | 37.74% | 11.02% | motif file (matrix) |
| 9 | 0.626 | A G T C A G T C C T A G A T G C G T A C C G T A C T A G C T A G | 1e-336 | -774.582804 | 65.38% | 37.03% | motif file (matrix) |
| 10 | 0.614 | G A C T C T G A T A C G C T G A C T A G T A C G A T C G G T A C C A T G T A G C A G C T A T G C A G C T | 1e-292 | -673.393791 | 7.90% | 0.53% | motif file (matrix) |
| 11 | 0.652 | G T A C A T G C G A C T A G T C A G C T C T A G A C T G A G T C A C T G C T A G A G C T A T G C C G T A | 1e-202 | -466.550611 | 4.72% | 0.23% | motif file (matrix) |
| 12 | 0.659 | A T G C G A T C G T C A A T C G T C G A T A C G C T A G T A C G A G T C A C T G A G T C A G T C C T G A | 1e-167 | -385.914667 | 4.23% | 0.24% | motif file (matrix) |
| 13 | 0.690 | A G T C C G T A G T A C C G T A A T G C A C T G C T A G A C G T A C T G C T A G | 1e-40 | -92.858368 | 4.51% | 1.54% | motif file (matrix) |
