# Supplementary material for: Detailed molecular and epigenetic characterization of the pig IPEC-J2 and chicken SL-29 cell lines
Source: iScience. 2023 Feb 20;26(3):106252. doi: 10.1016/j.isci.2023.106252 (PMC10018572; doi:10.1016/j.isci.2023.106252)
Supplement: Data S1. Complete homer output for identified motifs in Pig IPECJ-2, related to Table 2 — Homer motif analysis results for histone modifications H3K4me1, H3K4me3, H3K27ac, and enhancer elements of pig IPECJ2 cell line. P-values >1e-10 are possible false positives. Within each folder (e.g. peak_files_CTCF) are the html files showing the identified motifs when using homer (e.g. homerResults.html). [file mmc2.zip › S5/Pig_IPECJ_2/peak_fileS_CTCF/homerResults/motif2.similar.html]

motif2

## Information for motif2

C
T
A
G
A
C
T
G
A
G
T
C
T
A
C
G
A
T
G
C
G
A
C
T
T
G
C
A
A
G
C
T
  
Reverse Opposite:  

T
C
G
A
A
C
G
T
C
T
G
A
T
A
C
G
A
T
G
C
A
C
T
G
T
G
A
C
A
G
T
C
  

|  |  |
| --- | --- |
| p-value: | 1e-249 |
| log p-value: | -5.748e+02 |
| Information Content per bp: | 1.597 |
| Number of Target Sequences with motif | 2645.0 |
| Percentage of Target Sequences with motif | 56.49% |
| Number of Background Sequences with motif | 13725.6 |
| Percentage of Background Sequences with motif | 32.44% |
| Average Position of motif in Targets | 149.3 +/- 74.2bp |
| Average Position of motif in Background | 149.3 +/- 103.5bp |
| Strand Bias (log2 ratio + to - strand density) | 0.0 |
| Multiplicity (# of sites on avg that occur together) | 1.61 |
| Motif File: | file (matrix) reverse opposite |

### Similar de novo motifs found

|  |  |  |  |  |  |  |  |
| --- | --- | --- | --- | --- | --- | --- | --- |
| Rank | Match Score | Redundant Motif | P-value | log P-value | % of Targets | % of Background | Motif file |
| 1 | 0.617 | C T G A G T C A C G T A C T G A A T G C G T A C C T A G T A G C T A G C A G T C | 1e-58 | -135.043550 | 41.29% | 30.10% | motif file (matrix) |
| 2 | 0.705 | G C T A C T G A G C A T C G A T A T G C A C T G A G T C G A T C | 1e-46 | -106.104056 | 61.83% | 51.42% | motif file (matrix) |
| 3 | 0.631 | A T C G A C T G G A T C C T A G T C A G C T G A C T G A G T A C | 1e-44 | -101.861248 | 54.85% | 44.61% | motif file (matrix) |
| 4 | 0.614 | T C G A G C T A C G A T T C A G G T A C T A C G C A G T A G T C | 1e-18 | -42.408551 | 16.04% | 11.67% | motif file (matrix) |
| 5 | 0.657 | T A G C A G C T G C T A C T G A C A T G A T G C A T G C A G T C A G C T C G T A | 1e-15 | -35.274054 | 5.17% | 2.97% | motif file (matrix) |
| 6 | 0.601 | C T G A C G A T C A T G C A T G G A C T C T G A C T A G A G T C A C T G G C T A C G A T G T C A T G C A | 1e-5 | -12.144097 | 0.51% | 0.18% | motif file (matrix) |
