# Supplementary material for: Detailed molecular and epigenetic characterization of the pig IPEC-J2 and chicken SL-29 cell lines
Source: iScience. 2023 Feb 20;26(3):106252. doi: 10.1016/j.isci.2023.106252 (PMC10018572; doi:10.1016/j.isci.2023.106252)
Supplement: Data S1. Complete homer output for identified motifs in Pig IPECJ-2, related to Table 2 — Homer motif analysis results for histone modifications H3K4me1, H3K4me3, H3K27ac, and enhancer elements of pig IPECJ2 cell line. P-values >1e-10 are possible false positives. Within each folder (e.g. peak_files_CTCF) are the html files showing the identified motifs when using homer (e.g. homerResults.html). [file mmc2.zip › S5/Pig_IPECJ_2/peak_fileS_CTCF/homerResults/motif23.info.html]

Motif 23

## Information for 19-TTCACTGGBCTTA (Motif 23)

C
G
A
T
A
C
G
T
A
G
T
C
G
T
C
A
A
G
T
C
C
G
A
T
A
C
T
G
A
C
T
G
A
T
C
G
A
G
T
C
A
G
C
T
A
G
C
T
C
T
G
A
  
Reverse Opposite:  
