# Supplementary material for: Detailed molecular and epigenetic characterization of the pig IPEC-J2 and chicken SL-29 cell lines
Source: iScience. 2023 Feb 20;26(3):106252. doi: 10.1016/j.isci.2023.106252 (PMC10018572; doi:10.1016/j.isci.2023.106252)
Supplement: Data S1. Complete homer output for identified motifs in Pig IPECJ-2, related to Table 2 — Homer motif analysis results for histone modifications H3K4me1, H3K4me3, H3K27ac, and enhancer elements of pig IPECJ2 cell line. P-values >1e-10 are possible false positives. Within each folder (e.g. peak_files_CTCF) are the html files showing the identified motifs when using homer (e.g. homerResults.html). [file mmc2.zip › S5/Pig_IPECJ_2/peak_fileS_CTCF/homerResults/motif29.similar.html]

motif29

## Information for motif29

C
T
A
G
A
C
G
T
A
C
G
T
A
G
T
C
A
C
G
T
C
T
G
A
A
C
G
T
A
C
G
T
A
G
T
C
A
C
T
G
  
Reverse Opposite:  

G
T
A
C
A
C
T
G
C
G
T
A
C
G
T
A
A
G
C
T
C
G
T
A
A
C
T
G
C
G
T
A
C
G
T
A
A
G
T
C
  

|  |  |
| --- | --- |
| p-value: | 1e-2 |
| log p-value: | -5.927e+00 |
| Information Content per bp: | 1.926 |
| Number of Target Sequences with motif | 18.0 |
| Percentage of Target Sequences with motif | 0.38% |
| Number of Background Sequences with motif | 76.2 |
| Percentage of Background Sequences with motif | 0.18% |
| Average Position of motif in Targets | 143.4 +/- 90.8bp |
| Average Position of motif in Background | 151.4 +/- 85.2bp |
| Strand Bias (log2 ratio + to - strand density) | -0.3 |
| Multiplicity (# of sites on avg that occur together) | 4.17 |
| Motif File: | file (matrix) reverse opposite |

### Similar de novo motifs found

|  |  |  |  |  |  |  |  |
| --- | --- | --- | --- | --- | --- | --- | --- |
| Rank | Match Score | Redundant Motif | P-value | log P-value | % of Targets | % of Background | Motif file |
| 1 | 0.730 | A C G T C G A T A G T C A G C T C T G A A C G T C G A T A G T C G A C T C T G A C G A T A C G T A G T C | 1e-2 | -5.169312 | 0.04% | 0.00% | motif file (matrix) |
| 2 | 0.903 | A C T G C G T A C G T A A C G T C G T A A C T G C G T A C G T A | 1e0 | -0.352850 | 0.64% | 0.70% | motif file (matrix) |
