# Supplementary material for: Detailed molecular and epigenetic characterization of the pig IPEC-J2 and chicken SL-29 cell lines
Source: iScience. 2023 Feb 20;26(3):106252. doi: 10.1016/j.isci.2023.106252 (PMC10018572; doi:10.1016/j.isci.2023.106252)
Supplement: Data S1. Complete homer output for identified motifs in Pig IPECJ-2, related to Table 2 — Homer motif analysis results for histone modifications H3K4me1, H3K4me3, H3K27ac, and enhancer elements of pig IPECJ2 cell line. P-values >1e-10 are possible false positives. Within each folder (e.g. peak_files_CTCF) are the html files showing the identified motifs when using homer (e.g. homerResults.html). [file mmc2.zip › S5/Pig_IPECJ_2/peak_fileS_CTCF/homerResults/motif5.similar.html]

motif5

## Information for motif5

G
A
T
C
C
T
G
A
T
A
G
C
G
C
A
T
A
C
T
G
T
A
G
C
G
T
C
A
A
C
G
T
C
A
G
T
A
T
C
G
G
C
T
A
T
C
G
A
C
A
G
T
  
Reverse Opposite:  

G
T
C
A
A
G
C
T
C
G
A
T
T
A
G
C
G
T
C
A
G
T
C
A
C
A
G
T
A
T
C
G
G
T
A
C
C
G
T
A
A
T
C
G
G
A
C
T
C
T
A
G
  

|  |  |
| --- | --- |
| p-value: | 1e-73 |
| log p-value: | -1.694e+02 |
| Information Content per bp: | 1.665 |
| Number of Target Sequences with motif | 35.0 |
| Percentage of Target Sequences with motif | 0.75% |
| Number of Background Sequences with motif | 1.6 |
| Percentage of Background Sequences with motif | 0.00% |
| Average Position of motif in Targets | 149.9 +/- 84.3bp |
| Average Position of motif in Background | 124.1 +/- 48.6bp |
| Strand Bias (log2 ratio + to - strand density) | -0.5 |
| Multiplicity (# of sites on avg that occur together) | 4.77 |
| Motif File: | file (matrix) reverse opposite |

### Similar de novo motifs found

|  |  |  |  |  |  |  |  |
| --- | --- | --- | --- | --- | --- | --- | --- |
| Rank | Match Score | Redundant Motif | P-value | log P-value | % of Targets | % of Background | Motif file |
| 1 | 0.866 | C T A G T G A C C A G T A C T G G T A C G T C A A C G T C G T A T C A G T G C A G T C A C G A T G A T C | 1e-63 | -145.261581 | 0.75% | 0.01% | motif file (matrix) |
| 2 | 0.803 | T G A C A C G T A C T G A G T C C G T A A C G T A C T G A C T G C G T A T G C A A C G T T G A C A G T C | 1e-56 | -131.177070 | 0.75% | 0.01% | motif file (matrix) |
| 3 | 0.793 | C G T A A T G C A C G T C A T G G A T C C T G A C G A T A C G T A G T C G T C A G T C A C G A T A G T C | 1e-49 | -113.511689 | 0.75% | 0.01% | motif file (matrix) |
| 4 | 0.758 | C G T A G A T C C T A G G T A C C G A T A C T G A G T C G T C A A C G T A C T G A C T G C T G A T G C A | 1e-33 | -77.040549 | 1.17% | 0.13% | motif file (matrix) |
| 5 | 0.875 | A C G T A C T G G T A C C G T A A C G T A C G T A T G C C T G A C T G A A C G T | 1e-32 | -74.920241 | 0.77% | 0.04% | motif file (matrix) |
| 6 | 0.774 | A C G T A C T G A G T C C G T A A C G T A T G C A C T G C G T A G T C A A C G T | 1e-32 | -74.920241 | 0.77% | 0.04% | motif file (matrix) |
| 7 | 0.715 | C G T A C G T A A C G T A C T G A G T C C G T A A C T G A C G T A C T G A C T G | 1e-29 | -67.364229 | 0.79% | 0.05% | motif file (matrix) |
| 8 | 0.608 | C G T A G A C T A C T G A G T C C G A T A C T G G A T C T C G A G C A T C T A G | 1e-16 | -38.933451 | 0.75% | 0.12% | motif file (matrix) |
| 9 | 0.699 | A C G T A G T C A G T C C G T A A C G T A C T G A G T C C G T A | 1e-5 | -12.955838 | 1.26% | 0.65% | motif file (matrix) |
| 10 | 0.727 | G T A C C G T A A G C T A C G T A T G C T G C A C T G A G A C T | 1e-5 | -12.934611 | 1.11% | 0.54% | motif file (matrix) |
| 11 | 0.673 | A G T C C G T A C T A G A C G T A T C G A C T G C G T A A C G T | 1e-3 | -7.939350 | 1.67% | 1.11% | motif file (matrix) |
