# Supplementary material for: Detailed molecular and epigenetic characterization of the pig IPEC-J2 and chicken SL-29 cell lines
Source: iScience. 2023 Feb 20;26(3):106252. doi: 10.1016/j.isci.2023.106252 (PMC10018572; doi:10.1016/j.isci.2023.106252)
Supplement: Data S1. Complete homer output for identified motifs in Pig IPECJ-2, related to Table 2 — Homer motif analysis results for histone modifications H3K4me1, H3K4me3, H3K27ac, and enhancer elements of pig IPECJ2 cell line. P-values >1e-10 are possible false positives. Within each folder (e.g. peak_files_CTCF) are the html files showing the identified motifs when using homer (e.g. homerResults.html). [file mmc2.zip › S5/Pig_IPECJ_2/peak_fileS_CTCF/knownResults.html]

peak\_files - Homer Known Motif Enrichment Results


# Homer Known Motif Enrichment Results (peak\_files)

Homer *de novo* Motif Results  
Gene Ontology Enrichment Results  
Known Motif Enrichment Results (txt file)  
Total Target Sequences = 4682, Total Background Sequences = 42397

|  |  |  |  |  |  |  |  |  |  |  |  |
| --- | --- | --- | --- | --- | --- | --- | --- | --- | --- | --- | --- |
| Rank | Motif | Name | P-value | log P-pvalue | q-value (Benjamini) | # Target Sequences with Motif | % of Targets Sequences with Motif | # Background Sequences with Motif | % of Background Sequences with Motif | Motif File | SVG |
| 1 | T G C A A G C T C T G A A T C G G A C T C T A G G T A C G A T C G T C A A G T C G T A C G A C T C T A G A T C G G C A T C A T G C A T G G A T C G T A C C T G A | CTCF(Zf)/CD4+-CTCF-ChIP-Seq(Barski\_et\_al.)/Homer | 1e-3623 | -8.343e+03 | 0.0000 | 2946.0 | 62.92% | 886.1 | 2.09% | motif file (matrix) | svg |
| 2 | T A G C A G T C T G A C A G T C C T A G A T C G A G T C C A T G T G A C A G T C G T A C A G T C A G T C G C A T C T A G A T C G G C A T A C T G A T C G G A T C | BORIS(Zf)/K562-CTCFL-ChIP-Seq(GSE32465)/Homer | 1e-2963 | -6.824e+03 | 0.0000 | 3305.0 | 70.59% | 2333.3 | 5.51% | motif file (matrix) | svg |
| 3 | C G A T T A C G G A T C G C T A T C A G G A C T C G A T G T A C G A T C G T C A T C G A T G A C C G T A C T A G G A C T C T A G C T A G G T A C A G T C C G T A | CTCF-SatelliteElement(Zf?)/CD4+-CTCF-ChIP-Seq(Barski\_et\_al.)/Homer | 1e-114 | -2.634e+02 | 0.0000 | 127.0 | 2.71% | 58.6 | 0.14% | motif file (matrix) | svg |
| 4 | G A C T A G T C C G A T A C T G C T G A T G A C G T A C C G T A A T C G G C A T C T G A C T A G | Bcl11a(Zf)/HSPC-BCL11A-ChIP-Seq(GSE104676)/Homer | 1e-80 | -1.857e+02 | 0.0000 | 860.0 | 18.37% | 3940.6 | 9.31% | motif file (matrix) | svg |
| 5 | T C A G T G A C G T A C C G T A A C G T T G A C A C G T T C A G A G C T G A C T | NeuroD1(bHLH)/Islet-NeuroD1-ChIP-Seq(GSE30298)/Homer | 1e-49 | -1.144e+02 | 0.0000 | 880.0 | 18.80% | 4794.9 | 11.33% | motif file (matrix) | svg |
| 6 | C A T G A C T G A G C T A T G C C G T A A T G C G T A C G A C T T A C G C T G A A C T G A C T G G C A T A T G C C T G A | THRb(NR)/HepG2-THRb.Flag-ChIP-Seq(Encode)/Homer | 1e-47 | -1.094e+02 | 0.0000 | 763.0 | 16.30% | 4017.8 | 9.50% | motif file (matrix) | svg |
| 7 | T G A C G C T A T G A C C G T A T C A G G A T C C G T A C A T G C A T G C T A G C T A G C T A G | Unknown-ESC-element(?)/mES-Nanog-ChIP-Seq(GSE11724)/Homer | 1e-44 | -1.027e+02 | 0.0000 | 841.0 | 17.96% | 4656.8 | 11.01% | motif file (matrix) | svg |
| 8 | C A T G T A C G T A G C G A T C G A T C A T G C G T A C G A C T T C A G A T G C C G A T A T C G C A G T A C T G G T A C | Zic3(Zf)/mES-Zic3-ChIP-Seq(GSE37889)/Homer | 1e-40 | -9.425e+01 | 0.0000 | 1015.0 | 21.68% | 6069.3 | 14.34% | motif file (matrix) | svg |
| 9 | T C G A T G C A A C T G G T C A A G T C A G T C A G T C A G T C A G C T T G A C | LRF(Zf)/Erythroblasts-ZBTB7A-ChIP-Seq(GSE74977)/Homer | 1e-36 | -8.518e+01 | 0.0000 | 2026.0 | 43.27% | 14487.4 | 34.24% | motif file (matrix) | svg |
| 10 | T A G C G C A T A G T C G A T C A T G C G A C T C T A G A C T G A C T G C T G A A C T G C T A G A G T C T G A C C G A T | GLIS3(Zf)/Thyroid-Glis3.GFP-ChIP-Seq(GSE103297)/Homer | 1e-30 | -6.953e+01 | 0.0000 | 1899.0 | 40.56% | 13763.5 | 32.53% | motif file (matrix) | svg |
| 11 | A T G C T G C A A G T C C G T A A G T C A G T C A C G T A C T G A T G C G T C A | E2A(bHLH),near\_PU.1/Bcell-PU.1-ChIP-Seq(GSE21512)/Homer | 1e-22 | -5.250e+01 | 0.0000 | 1529.0 | 32.66% | 11052.0 | 26.12% | motif file (matrix) | svg |
| 12 | T G C A G T A C C G T A A T C G A C T G A C G T C T A G C G A T T C G A A G T C | ZEB1(Zf)/PDAC-ZEB1-ChIP-Seq(GSE64557)/Homer | 1e-16 | -3.859e+01 | 0.0000 | 1612.0 | 34.43% | 12162.8 | 28.75% | motif file (matrix) | svg |
| 13 | G A C T C T A G A T G C A G T C G T C A T A C G A T G C A T C G | HIC1(Zf)/Treg-ZBTB29-ChIP-Seq(GSE99889)/Homer | 1e-15 | -3.572e+01 | 0.0000 | 1965.0 | 41.97% | 15325.5 | 36.22% | motif file (matrix) | svg |
| 14 | C T G A C T A G A T C G A G C T A C T G G A C T A G T C C T G A | Tbx5(T-box)/HL1-Tbx5.biotin-ChIP-Seq(GSE21529)/Homer | 1e-12 | -2.983e+01 | 0.0000 | 2139.0 | 45.69% | 17086.8 | 40.38% | motif file (matrix) | svg |
| 15 | T G C A T C G A T A G C G T A C T C A G C T A G G T C A G C T A T C A G G A C T | ETS(ETS)/Promoter/Homer | 1e-12 | -2.872e+01 | 0.0000 | 326.0 | 6.96% | 1945.8 | 4.60% | motif file (matrix) | svg |
| 16 | A G C T C A T G G C A T G A T C T G C A C T A G G A T C A C G T | Tgif2(Homeobox)/mES-Tgif2-ChIP-Seq(GSE55404)/Homer | 1e-11 | -2.724e+01 | 0.0000 | 1900.0 | 40.58% | 15079.4 | 35.64% | motif file (matrix) | svg |
| 17 | G A T C C T G A A G T C C G A T C G A T G A T C A G T C A C T G A T C G A G C T | Elk4(ETS)/Hela-Elk4-ChIP-Seq(GSE31477)/Homer | 1e-10 | -2.529e+01 | 0.0000 | 588.0 | 12.56% | 4040.8 | 9.55% | motif file (matrix) | svg |
| 18 | G A T C T C G A A G T C C G A T C G A T A G T C A T G C A C T G A T C G G A C T | Elk1(ETS)/Hela-Elk1-ChIP-Seq(GSE31477)/Homer | 1e-10 | -2.526e+01 | 0.0000 | 578.0 | 12.35% | 3961.1 | 9.36% | motif file (matrix) | svg |
| 19 | C T G A T G C A T A G C T G A C T A C G T C A G C T G A G C T A T C A G G A C T | ELF1(ETS)/Jurkat-ELF1-ChIP-Seq(SRA014231)/Homer | 1e-10 | -2.515e+01 | 0.0000 | 525.0 | 11.21% | 3543.7 | 8.38% | motif file (matrix) | svg |
| 20 | T A G C T A G C G C A T C A T G A C T G G C T A C G T A A C G T A C T G G A T C | TEAD4(TEA)/Tropoblast-Tead4-ChIP-Seq(GSE37350)/Homer | 1e-10 | -2.495e+01 | 0.0000 | 535.0 | 11.43% | 3627.4 | 8.57% | motif file (matrix) | svg |
| 21 | G A C T T C G A C G A T T A C G C T A G T A C G A C T G A T G C G T A C G T A C | Zac1(Zf)/Neuro2A-Plagl1-ChIP-Seq(GSE75942)/Homer | 1e-10 | -2.388e+01 | 0.0000 | 2460.0 | 52.54% | 20219.1 | 47.79% | motif file (matrix) | svg |
| 22 | A G T C C T G A A G T C C G A T C A G T G A T C A T G C A C T G A T C G G A C T | Fli1(ETS)/CD8-FLI-ChIP-Seq(GSE20898)/Homer | 1e-10 | -2.305e+01 | 0.0000 | 959.0 | 20.48% | 7149.7 | 16.90% | motif file (matrix) | svg |
| 23 | T G A C A G T C C T G A T G A C C G T A A C G T A C G T A G T C A G T C C G T A | TEAD1(TEAD)/HepG2-TEAD1-ChIP-Seq(Encode)/Homer | 1e-9 | -2.295e+01 | 0.0000 | 601.0 | 12.84% | 4206.0 | 9.94% | motif file (matrix) | svg |
| 24 | A G C T C T A G T G A C C G T A A C G T C G A T A G T C A G T C C T G A C A T G | TEAD3(TEA)/HepG2-TEAD3-ChIP-Seq(Encode)/Homer | 1e-9 | -2.295e+01 | 0.0000 | 672.0 | 14.35% | 4781.9 | 11.30% | motif file (matrix) | svg |
| 25 | T A C G T A C G G T A C A T C G T A C G T A C G G T C A C T G A C G T A G A C T | E2F4(E2F)/K562-E2F4-ChIP-Seq(GSE31477)/Homer | 1e-9 | -2.248e+01 | 0.0000 | 585.0 | 12.49% | 4090.4 | 9.67% | motif file (matrix) | svg |
| 26 | C T G A T A G C T G A C T C A G C T A G G T C A C G T A T C A G A G C T T C A G | ETV4(ETS)/HepG2-ETV4-ChIP-Seq(ENCODE)/Homer | 1e-9 | -2.222e+01 | 0.0000 | 989.0 | 21.12% | 7431.0 | 17.56% | motif file (matrix) | svg |
| 27 | A T C G A G C T A C T G A G T C A C T G A G T C C G T A A C G T A C T G A G T C A C T G A G T C | NRF(NRF)/Promoter/Homer | 1e-9 | -2.113e+01 | 0.0000 | 225.0 | 4.81% | 1327.9 | 3.14% | motif file (matrix) | svg |
| 28 | T A C G C T A G A T G C G A T C G T A C A G T C C T A G A G T C A G T C A G T C G T A C A G T C | Sp1(Zf)/Promoter/Homer | 1e-8 | -1.988e+01 | 0.0000 | 544.0 | 11.62% | 3832.1 | 9.06% | motif file (matrix) | svg |
| 29 | C T A G A G T C T A C G T A C G T G A C C G T A A C T G T A G C G C A T C A T G A T G C A G C T | Ascl1(bHLH)/NeuralTubes-Ascl1-ChIP-Seq(GSE55840)/Homer | 1e-8 | -1.934e+01 | 0.0000 | 1589.0 | 33.94% | 12701.7 | 30.02% | motif file (matrix) | svg |
| 30 | T G A C G T C A A G T C G C T A C T A G A G T C C T G A C A T G A C T G C T A G T A C G T C A G | Zic2(Zf)/ESC-Zic2-ChIP-Seq(SRP197560)/Homer | 1e-8 | -1.927e+01 | 0.0000 | 683.0 | 14.59% | 4984.9 | 11.78% | motif file (matrix) | svg |
| 31 | T C G A A G T C C G T A A T C G T A G C A C G T A C T G A G C T A C G T A G T C | Ptf1a(bHLH)/Panc1-Ptf1a-ChIP-Seq(GSE47459)/Homer | 1e-8 | -1.888e+01 | 0.0000 | 2244.0 | 47.93% | 18524.0 | 43.78% | motif file (matrix) | svg |
| 32 | A C T G C A T G T C G A A C G T A C T G C G T A A T C G A C G T G T A C C G T A G A C T A G T C | Fos(bZIP)/TSC-Fos-ChIP-Seq(GSE110950)/Homer | 1e-8 | -1.852e+01 | 0.0000 | 304.0 | 6.49% | 1969.7 | 4.66% | motif file (matrix) | svg |
| 33 | T C G A T A G C G T C A A C T G A C T G C G T A C G T A C T A G A G C T T C A G | ERG(ETS)/VCaP-ERG-ChIP-Seq(GSE14097)/Homer | 1e-7 | -1.739e+01 | 0.0000 | 1164.0 | 24.86% | 9110.7 | 21.53% | motif file (matrix) | svg |
| 34 | T C G A T A G C T G C A A C T G A C T G C G T A C G T A C T A G G A C T T A C G | ETS1(ETS)/Jurkat-ETS1-ChIP-Seq(GSE17954)/Homer | 1e-7 | -1.627e+01 | 0.0000 | 793.0 | 16.94% | 6006.1 | 14.20% | motif file (matrix) | svg |
| 35 | A T G C G A C T A G C T C T A G C G T A C T A G C G A T C T A G A T C G G A T C | Nkx2.2(Homeobox)/NPC-Nkx2.2-ChIP-Seq(GSE61673)/Homer | 1e-6 | -1.602e+01 | 0.0000 | 1163.0 | 24.84% | 9164.5 | 21.66% | motif file (matrix) | svg |
| 36 | C T G A T C A G C A G T C T A G A C T G C T A G G A T C A T C G A C T G C T G A T C A G G A T C | Sp5(Zf)/mES-Sp5.Flag-ChIP-Seq(GSE72989)/Homer | 1e-6 | -1.594e+01 | 0.0000 | 1288.0 | 27.51% | 10247.7 | 24.22% | motif file (matrix) | svg |
| 37 | C T A G T C G A A C G T A C T G C G T A T A G C C G A T G T A C C G T A A G C T G A T C G T A C | Jun-AP1(bZIP)/K562-cJun-ChIP-Seq(GSE31477)/Homer | 1e-6 | -1.591e+01 | 0.0000 | 143.0 | 3.05% | 814.9 | 1.93% | motif file (matrix) | svg |
| 38 | T C G A T C G A T A G C G T A C T C A G T A C G C G T A C G T A T C A G A G C T | GABPA(ETS)/Jurkat-GABPa-ChIP-Seq(GSE17954)/Homer | 1e-6 | -1.583e+01 | 0.0000 | 719.0 | 15.36% | 5403.6 | 12.77% | motif file (matrix) | svg |
| 39 | C T A G T C G A G C A T C A T G G C T A T A G C C G A T G T A C C T G A A G C T | JunB(bZIP)/DendriticCells-Junb-ChIP-Seq(GSE36099)/Homer | 1e-6 | -1.551e+01 | 0.0000 | 293.0 | 6.26% | 1951.1 | 4.61% | motif file (matrix) | svg |
| 40 | A T G C G A T C C G A T C T A G A C T G G C T A C G T A A G C T A C T G A G C T | TEAD2(TEA)/Py2T-Tead2-ChIP-Seq(GSE55709)/Homer | 1e-6 | -1.487e+01 | 0.0000 | 314.0 | 6.71% | 2131.5 | 5.04% | motif file (matrix) | svg |
| 41 | G C T A A G T C T A C G T G C A A T C G T C A G G C T A T C G A T C A G A G C T | ELF5(ETS)/T47D-ELF5-ChIP-Seq(GSE30407)/Homer | 1e-6 | -1.468e+01 | 0.0000 | 514.0 | 10.98% | 3747.9 | 8.86% | motif file (matrix) | svg |
| 42 | T G A C G T A C C G T A A C T G T G A C C G A T A C T G A T C G A G C T T A C G T C G A T A G C G T A C C G T A A T C G T G A C G C A T A C T G A C T G A T G C | Twist(bHLH)/HMLE-TWIST1-ChIP-Seq(Chang\_et\_al)/Homer | 1e-6 | -1.466e+01 | 0.0000 | 134.0 | 2.86% | 768.7 | 1.82% | motif file (matrix) | svg |
| 43 | A G T C C T A G C T A G A G T C G A T C G T A C A G T C C T A G A G T C A G T C A G T C G T A C | Sp2(Zf)/HEK293-Sp2.eGFP-ChIP-Seq(Encode)/Homer | 1e-6 | -1.439e+01 | 0.0000 | 1746.0 | 37.29% | 14338.4 | 33.89% | motif file (matrix) | svg |
| 44 | G A C T C T A G C T A G A G T C T G C A A C T G A C G T A C G T C T A G T C A G | AMYB(HTH)/Testes-AMYB-ChIP-Seq(GSE44588)/Homer | 1e-6 | -1.416e+01 | 0.0000 | 916.0 | 19.56% | 7135.3 | 16.86% | motif file (matrix) | svg |
| 45 | G A T C G T A C C G A T A C T G A C T G C G T A C G T A A C G T A C T G G A T C | TEAD(TEA)/Fibroblast-PU.1-ChIP-Seq(Unpublished)/Homer | 1e-6 | -1.407e+01 | 0.0000 | 380.0 | 8.12% | 2678.1 | 6.33% | motif file (matrix) | svg |
| 46 | T G A C A T G C C G T A A T C G A T G C C A G T C A T G A C T G A G T C G T A C | HEB(bHLH)/mES-Heb-ChIP-Seq(GSE53233)/Homer | 1e-6 | -1.400e+01 | 0.0000 | 1839.0 | 39.28% | 15184.9 | 35.89% | motif file (matrix) | svg |
| 47 | A T G C A G C T T C A G T G A C T C A G A T G C T G C A A C G T A T C G G A T C A C T G A G T C | NRF1(NRF)/MCF7-NRF1-ChIP-Seq(Unpublished)/Homer | 1e-5 | -1.353e+01 | 0.0000 | 231.0 | 4.93% | 1515.3 | 3.58% | motif file (matrix) | svg |
| 48 | C T A G C T A G A G T C T C A G A C T G A C G T A C G T C T G A | MYB(HTH)/ERMYB-Myb-ChIPSeq(GSE22095)/Homer | 1e-5 | -1.350e+01 | 0.0000 | 1076.0 | 22.98% | 8537.6 | 20.18% | motif file (matrix) | svg |
| 49 | C T A G T C G A A C G T A C T G C G T A A T G C A C G T G T A C C G T A A G C T G A T C G T A C | Atf3(bZIP)/GBM-ATF3-ChIP-Seq(GSE33912)/Homer | 1e-5 | -1.323e+01 | 0.0000 | 344.0 | 7.35% | 2413.6 | 5.70% | motif file (matrix) | svg |
| 50 | T C G A T C A G T C G A A C T G C A T G A C G T A G T C C T G A | COUP-TFII(NR)/Artia-Nr2f2-ChIP-Seq(GSE46497)/Homer | 1e-5 | -1.322e+01 | 0.0000 | 1379.0 | 29.45% | 11180.9 | 26.43% | motif file (matrix) | svg |
| 51 | A C T G A C G T C A T G A T C G A T C G T G A C A C T G A T C G A T C G T G C A C T G A C G T A | E2F3(E2F)/MEF-E2F3-ChIP-Seq(GSE71376)/Homer | 1e-5 | -1.315e+01 | 0.0000 | 803.0 | 17.15% | 6220.8 | 14.70% | motif file (matrix) | svg |
| 52 | A T G C A G T C C T G A A G T C C G A T A C G T A G T C A G T C A C G T A T C G G A C T A C G T | Etv2(ETS)/ES-ER71-ChIP-Seq(GSE59402)/Homer | 1e-5 | -1.300e+01 | 0.0000 | 667.0 | 14.25% | 5079.1 | 12.00% | motif file (matrix) | svg |
| 53 | C T A G C A T G A C G T A G T C G C T A A G C T A G T C A G C T T C A G C T G A A C T G C A T G G C A T A T G C C G T A | THRa(NR)/C17.2-THRa-ChIP-Seq(GSE38347)/Homer | 1e-5 | -1.289e+01 | 0.0000 | 345.0 | 7.37% | 2431.6 | 5.75% | motif file (matrix) | svg |
| 54 | T A C G C T A G T A C G A G T C C G T A A G T C A G T C A C G T A C T G A G T C G A T C T A G C | Slug(Zf)/Mesoderm-Snai2-ChIP-Seq(GSE61475)/Homer | 1e-5 | -1.277e+01 | 0.0000 | 726.0 | 15.51% | 5585.4 | 13.20% | motif file (matrix) | svg |
| 55 | T C A G C T A G C T A G A C T G A C T G G A T C A C T G A C T G C T A G C T A G A G T C G A T C | KLF1(Zf)/HUDEP2-KLF1-CutnRun(GSE136251)/Homer | 1e-5 | -1.266e+01 | 0.0000 | 1147.0 | 24.50% | 9194.0 | 21.73% | motif file (matrix) | svg |
| 56 | T C G A C T G A T A G C T G A C T C A G T C A G C G T A C G T A T C A G A G C T | ETV1(ETS)/GIST48-ETV1-ChIP-Seq(GSE22441)/Homer | 1e-5 | -1.259e+01 | 0.0000 | 1072.0 | 22.90% | 8549.6 | 20.21% | motif file (matrix) | svg |
| 57 | T C G A A C G T C A T G G C T A T A G C C G A T G T A C G C T A A C G T A T G C | AP-1(bZIP)/ThioMac-PU.1-ChIP-Seq(GSE21512)/Homer | 1e-5 | -1.223e+01 | 0.0000 | 385.0 | 8.22% | 2774.3 | 6.56% | motif file (matrix) | svg |
| 58 | C T A G T A C G A G T C C G T A A G T C A C G T A G T C T C G A C G T A T A C G | Nkx2.1(Homeobox)/LungAC-Nkx2.1-ChIP-Seq(GSE43252)/Homer | 1e-5 | -1.221e+01 | 0.0000 | 1594.0 | 34.05% | 13126.7 | 31.03% | motif file (matrix) | svg |
| 59 | T G C A T A G C G A C T T G C A T G A C T G C A C G T A A G C T A G C T A G T C A G T C G T A C | GFY(?)/Promoter/Homer | 1e-5 | -1.209e+01 | 0.0000 | 54.0 | 1.15% | 250.3 | 0.59% | motif file (matrix) | svg |
| 60 | C A T G C T A G T C G A A C G T A C T G C G T A T A G C C G A T T G A C C G T A A G C T G A T C | Fra2(bZIP)/Striatum-Fra2-ChIP-Seq(GSE43429)/Homer | 1e-5 | -1.203e+01 | 0.0000 | 256.0 | 5.47% | 1746.5 | 4.13% | motif file (matrix) | svg |
| 61 | T C A G T A C G T A G C A C G T A C T G C G A T A G T C C G T A T A C G A G T C | Meis1(Homeobox)/MastCells-Meis1-ChIP-Seq(GSE48085)/Homer | 1e-5 | -1.189e+01 | 0.0000 | 959.0 | 20.48% | 7612.8 | 17.99% | motif file (matrix) | svg |
| 62 | C G T A T A G C T A G C T G C A A C T G C T A G C G T A C G T A T C A G G A C T | EHF(ETS)/LoVo-EHF-ChIP-Seq(GSE49402)/Homer | 1e-5 | -1.173e+01 | 0.0001 | 877.0 | 18.73% | 6917.1 | 16.35% | motif file (matrix) | svg |
| 63 | C A G T T G C A A C G T A C T G C G T A A T C G C G A T T G A C C G T A A C G T | BATF(bZIP)/Th17-BATF-ChIP-Seq(GSE39756)/Homer | 1e-4 | -1.139e+01 | 0.0001 | 319.0 | 6.81% | 2266.8 | 5.36% | motif file (matrix) | svg |
| 64 | C T G A A G T C C G A T A G C T A T G C G T A C A C G T A T C G C A G T G C A T | Elf4(ETS)/BMDM-Elf4-ChIP-Seq(GSE88699)/Homer | 1e-4 | -1.126e+01 | 0.0001 | 753.0 | 16.08% | 5882.8 | 13.90% | motif file (matrix) | svg |
| 65 | A G C T G A C T C T A G C G T A C A T G C G A T C T A G A T C G G A C T C A G T | Bapx1(Homeobox)/VertebralCol-Bapx1-ChIP-Seq(GSE36672)/Homer | 1e-4 | -1.122e+01 | 0.0001 | 1128.0 | 24.09% | 9108.8 | 21.53% | motif file (matrix) | svg |
| 66 | C T A G T C G A C G A T A C T G C G T A T A C G A G C T T G A C G C T A A C G T G A T C T A G C | Fosl2(bZIP)/3T3L1-Fosl2-ChIP-Seq(GSE56872)/Homer | 1e-4 | -1.104e+01 | 0.0001 | 183.0 | 3.91% | 1200.9 | 2.84% | motif file (matrix) | svg |
| 67 | A G T C G C A T C G T A C G T A G T A C A C G T A C T G G A T C G A T C T C G A | BMYB(HTH)/Hela-BMYB-ChIP-Seq(GSE27030)/Homer | 1e-4 | -1.077e+01 | 0.0001 | 877.0 | 18.73% | 6966.6 | 16.47% | motif file (matrix) | svg |
| 68 | A C T G C T A G T C G A C G A T C A T G G C T A A T C G C G A T G T A C G C T A A G C T G T A C | Fra1(bZIP)/BT549-Fra1-ChIP-Seq(GSE46166)/Homer | 1e-4 | -1.060e+01 | 0.0002 | 274.0 | 5.85% | 1928.8 | 4.56% | motif file (matrix) | svg |
| 69 | T C G A A G T C C G T A A T C G A T G C C G A T A C T G A G T C A G C T A C T G | Tcf12(bHLH)/GM12878-Tcf12-ChIP-Seq(GSE32465)/Homer | 1e-4 | -1.037e+01 | 0.0002 | 1015.0 | 21.68% | 8177.2 | 19.33% | motif file (matrix) | svg |
| 70 | C G T A T A C G T C G A A C T G A C T G C G T A C G T A T A C G A G C T T A C G | PU.1(ETS)/ThioMac-PU.1-ChIP-Seq(GSE21512)/Homer | 1e-4 | -9.828e+00 | 0.0003 | 309.0 | 6.60% | 2234.2 | 5.28% | motif file (matrix) | svg |
| 71 | T A G C C G T A C T G A T A C G C G T A A C G T A C T G A C T G A G T C T A C G C T A G G T A C | YY1(Zf)/Promoter/Homer | 1e-4 | -9.609e+00 | 0.0004 | 65.0 | 1.39% | 350.2 | 0.83% | motif file (matrix) | svg |
| 72 | A G C T T C G A G T A C T C G A A T G C A T G C G C A T A T C G A G T C A G C T | Snail1(Zf)/LS174T-SNAIL1.HA-ChIP-Seq(GSE127183)/Homer | 1e-4 | -9.606e+00 | 0.0004 | 946.0 | 20.21% | 7624.0 | 18.02% | motif file (matrix) | svg |
| 73 | C G T A T G A C T A G C T G C A A C T G A C T G C G T A C G T A T C A G G A C T | ELF3(ETS)/PDAC-ELF3-ChIP-Seq(GSE64557)/Homer | 1e-3 | -8.850e+00 | 0.0009 | 483.0 | 10.32% | 3711.9 | 8.77% | motif file (matrix) | svg |
| 74 | C G T A C T A G T C A G T C A G A G T C A T G C A G T C G C A T A G C T A C G T A T C G C G A T | Sox9(HMG)/Limb-SOX9-ChIP-Seq(GSE73225)/Homer | 1e-3 | -8.712e+00 | 0.0010 | 521.0 | 11.13% | 4037.1 | 9.54% | motif file (matrix) | svg |
| 75 | C G A T T A C G T G A C G A C T C A T G C G T A T A C G A C G T G T A C C T G A | Bach2(bZIP)/OCILy7-Bach2-ChIP-Seq(GSE44420)/Homer | 1e-3 | -8.675e+00 | 0.0010 | 117.0 | 2.50% | 745.8 | 1.76% | motif file (matrix) | svg |
| 76 | A T G C C T G A A T C G T A C G A G T C C G A T T C A G C G A T C T A G A G C T G T C A G T C A C G T A A G T C C G T A T A C G C T G A | Fox:Ebox(Forkhead,bHLH)/Panc1-Foxa2-ChIP-Seq(GSE47459)/Homer | 1e-3 | -8.656e+00 | 0.0010 | 484.0 | 10.34% | 3728.1 | 8.81% | motif file (matrix) | svg |
| 77 | T A G C C G A T A C G T A G C T A G C T A G T C A T G C A G T C A C T G A T G C A T G C G C T A | E2F7(E2F)/Hela-E2F7-ChIP-Seq(GSE32673)/Homer | 1e-3 | -8.421e+00 | 0.0013 | 152.0 | 3.25% | 1020.7 | 2.41% | motif file (matrix) | svg |
| 78 | C T A G C T G A T C A G A T C G A G C T C A T G G A C T A G T C C T G A T G C A | Tbx6(T-box)/ESC-Tbx6-ChIP-Seq(GSE93524)/Homer | 1e-3 | -8.368e+00 | 0.0013 | 661.0 | 14.12% | 5243.1 | 12.39% | motif file (matrix) | svg |
| 79 | C T G A T C A G G T A C G C T A A C T G T G A C G C A T C A T G | SCL(bHLH)/HPC7-Scl-ChIP-Seq(GSE13511)/Homer | 1e-3 | -8.275e+00 | 0.0014 | 3114.0 | 66.51% | 27109.7 | 64.07% | motif file (matrix) | svg |
| 80 | A T G C G A C T A C G T C T A G A C G T A C G T A C G T C T G A G A T C G C T A A G C T C G T A | Foxa2(Forkhead)/Liver-Foxa2-ChIP-Seq(GSE25694)/Homer | 1e-3 | -7.907e+00 | 0.0020 | 384.0 | 8.20% | 2923.7 | 6.91% | motif file (matrix) | svg |
| 81 | T G C A A G C T A C G T C T A G G A T C C T A G G A T C G T C A C T G A A G T C | CEBP(bZIP)/ThioMac-CEBPb-ChIP-Seq(GSE21512)/Homer | 1e-3 | -7.833e+00 | 0.0022 | 298.0 | 6.36% | 2213.0 | 5.23% | motif file (matrix) | svg |
| 82 | C T A G T A C G G A T C G T A C G C T A A G C T A G C T G T C A T C G A T A G C | Nanog(Homeobox)/mES-Nanog-ChIP-Seq(GSE11724)/Homer | 1e-3 | -7.829e+00 | 0.0022 | 2155.0 | 46.03% | 18439.2 | 43.58% | motif file (matrix) | svg |
| 83 | A G T C G A C T C A G T G T A C A G T C A T C G T C A G A C T G G T C A C G T A | Stat3(Stat)/mES-Stat3-ChIP-Seq(GSE11431)/Homer | 1e-3 | -7.528e+00 | 0.0029 | 336.0 | 7.18% | 2539.7 | 6.00% | motif file (matrix) | svg |
| 84 | T C A G C G T A A G T C A G C T C G T A A G T C C T G A C G T A A G T C G C A T A G T C A G T C A G T C C T G A A C T G T G C A T C G A C A T G A T C G G A T C | Ronin(THAP)/ES-Thap11-ChIP-Seq(GSE51522)/Homer | 1e-3 | -7.333e+00 | 0.0034 | 23.0 | 0.49% | 96.4 | 0.23% | motif file (matrix) | svg |
| 85 | T G C A C T G A A G T C G T C A A C T G A C T G C G T A C G T A C T G A A G C T | EWS:FLI1-fusion(ETS)/SK\_N\_MC-EWS:FLI1-ChIP-Seq(SRA014231)/Homer | 1e-3 | -7.005e+00 | 0.0047 | 398.0 | 8.50% | 3080.8 | 7.28% | motif file (matrix) | svg |
| 86 | T G A C C T A G A C T G T A G C C G A T A C T G A T G C C A T G A T C G A T C G A T C G T A G C C T G A T A G C G C T A A C T G C G T A A G C T C G T A C T G A | GATA:SCL(Zf,bHLH)/Ter119-SCL-ChIP-Seq(GSE18720)/Homer | 1e-2 | -6.732e+00 | 0.0061 | 69.0 | 1.47% | 421.9 | 1.00% | motif file (matrix) | svg |
| 87 | T C G A C G T A A G T C A G C T C G T A A G T C T C G A G C T A G A C T C G A T A G T C A G T C A G T C C T G A T C A G T G C A T C G A C A G T A T C G A G T C | GFY-Staf(?,Zf)/Promoter/Homer | 1e-2 | -6.649e+00 | 0.0065 | 33.0 | 0.70% | 166.7 | 0.39% | motif file (matrix) | svg |
| 88 | T C A G A G C T A T G C C G T A A G C T T C A G C A G T A C T G C T G A A G T C | MITF(bHLH)/MastCells-MITF-ChIP-Seq(GSE48085)/Homer | 1e-2 | -6.241e+00 | 0.0097 | 542.0 | 11.58% | 4343.9 | 10.27% | motif file (matrix) | svg |
| 89 | C A T G A C G T A G T C G A T C G A T C G A T C G C T A C T A G C T A G C T A G T C A G T C G A | EBF1(EBF)/Near-E2A-ChIP-Seq(GSE21512)/Homer | 1e-2 | -6.148e+00 | 0.0106 | 1159.0 | 24.75% | 9720.2 | 22.97% | motif file (matrix) | svg |
| 90 | C G T A C G T A C G T A G C A T G C A T T A C G G T A C G A C T A C T G C G T A A T C G A C G T G T A C C G T A A G C T | Bach1(bZIP)/K562-Bach1-ChIP-Seq(GSE31477)/Homer | 1e-2 | -6.002e+00 | 0.0121 | 34.0 | 0.73% | 180.3 | 0.43% | motif file (matrix) | svg |
| 91 | A G C T C G A T T A C G A T C G A T C G C A G T A G T C A G T C A C T G T A G C | HINFP(Zf)/K562-HINFP.eGFP-ChIP-Seq(Encode)/Homer | 1e-2 | -5.915e+00 | 0.0130 | 463.0 | 9.89% | 3686.7 | 8.71% | motif file (matrix) | svg |
| 92 | T G C A C G T A A C T G T C A G C A G T C A T G T C A G G A T C T A C G A G T C T G C A A C T G A C T G T G A C G T C A | ZNF165(Zf)/WHIM12-ZNF165-ChIP-Seq(GSE65937)/Homer | 1e-2 | -5.874e+00 | 0.0134 | 199.0 | 4.25% | 1471.4 | 3.48% | motif file (matrix) | svg |
| 93 | A G T C C G A T C T G A C G T A A C G T C A G T T C A G T G A C | Isl1(Homeobox)/Neuron-Isl1-ChIP-Seq(GSE31456)/Homer | 1e-2 | -5.793e+00 | 0.0144 | 958.0 | 20.46% | 7984.0 | 18.87% | motif file (matrix) | svg |
| 94 | C T G A C G A T C T A G T C A G G A T C C T G A T C A G G A T C C T G A A C T G A G T C G C T A A C G T A G T C G C A T | PRDM9(Zf)/Testis-DMC1-ChIP-Seq(GSE35498)/Homer | 1e-2 | -5.722e+00 | 0.0153 | 342.0 | 7.30% | 2669.7 | 6.31% | motif file (matrix) | svg |
| 95 | C T G A C T G A T A G C G A T C G C T A G T A C A C G T G A T C T G C A C G T A | Nkx2.5(Homeobox)/HL1-Nkx2.5.biotin-ChIP-Seq(GSE21529)/Homer | 1e-2 | -5.571e+00 | 0.0176 | 1185.0 | 25.31% | 9998.3 | 23.63% | motif file (matrix) | svg |
| 96 | C T A G A C T G T G C A G T C A A T G C C G T A A T C G A T G C A G T C C T A G | ZNF341(Zf)/EBV-ZNF341-ChIP-Seq(GSE113194)/Homer | 1e-2 | -5.548e+00 | 0.0179 | 554.0 | 11.83% | 4488.0 | 10.61% | motif file (matrix) | svg |
| 97 | A G T C T G C A T C G A C T G A A C T G C A T G A C G T A T G C G T C A T A C G | Erra(NR)/HepG2-Erra-ChIP-Seq(GSE31477)/Homer | 1e-2 | -5.522e+00 | 0.0181 | 1752.0 | 37.42% | 15040.1 | 35.55% | motif file (matrix) | svg |
| 98 | G A C T T C A G C T A G A G T C A G T C G T A C A G T C C T G A A G T C A G T C A G T C G A C T A G T C A C T G A T G C | KLF3(Zf)/MEF-Klf3-ChIP-Seq(GSE44748)/Homer | 1e-2 | -5.426e+00 | 0.0198 | 619.0 | 13.22% | 5057.9 | 11.95% | motif file (matrix) | svg |
| 99 | A G T C G A T C G C T A C G A T A C G T T A C G G C A T C T G A G A C T A C T G A G T C G C T A C T G A T C G A C A G T | Oct4:Sox17(POU,Homeobox,HMG)/F9-Sox17-ChIP-Seq(GSE44553)/Homer | 1e-2 | -5.332e+00 | 0.0215 | 70.0 | 1.50% | 455.4 | 1.08% | motif file (matrix) | svg |
| 100 | T C G A A C G T A C T G C T G A A G T C T C A G A G C T G T A C C G T A A G C T G A T C T C G A | JunD(bZIP)/K562-JunD-ChIP-Seq/Homer | 1e-2 | -5.317e+00 | 0.0216 | 49.0 | 1.05% | 297.7 | 0.70% | motif file (matrix) | svg |
| 101 | A G T C A T C G C T A G A G C T G A C T C T A G A G T C A G T C G C T A C A G T T C A G T C A G G A T C C T G A T C G A G A T C | RFX(HTH)/K562-RFX3-ChIP-Seq(SRA012198)/Homer | 1e-2 | -5.285e+00 | 0.0221 | 67.0 | 1.43% | 433.4 | 1.02% | motif file (matrix) | svg |
| 102 | C G T A C T A G A C T G A C T G G A C T C T A G C A G T C T A G C A T G G A T C | KLF5(Zf)/LoVo-KLF5-ChIP-Seq(GSE49402)/Homer | 1e-2 | -5.242e+00 | 0.0228 | 1478.0 | 31.57% | 12625.2 | 29.84% | motif file (matrix) | svg |
| 103 | A G T C A C G T A C T G A G C T A C G T A C G T G T C A A G T C | Foxo1(Forkhead)/RAW-Foxo1-ChIP-Seq(Fan\_et\_al.)/Homer | 1e-2 | -5.142e+00 | 0.0250 | 1036.0 | 22.13% | 8722.4 | 20.62% | motif file (matrix) | svg |
| 104 | C G T A C T G A C G T A C T A G T C G A C T A G A C T G C G T A C G T A T A C G A G C T A T C G | SpiB(ETS)/OCILY3-SPIB-ChIP-Seq(GSE56857)/Homer | 1e-2 | -5.069e+00 | 0.0266 | 154.0 | 3.29% | 1131.0 | 2.67% | motif file (matrix) | svg |
| 105 | A G T C T A G C G A C T A C G T C T A G A C G T A C G T A C G T C T G A A G T C G C T A G A C T C G T A C T A G A C T G | Foxa3(Forkhead)/Liver-Foxa3-ChIP-Seq(GSE77670)/Homer | 1e-2 | -5.045e+00 | 0.0270 | 149.0 | 3.18% | 1091.8 | 2.58% | motif file (matrix) | svg |
| 106 | C T A G A T G C A T G C C G A T A C T G G A C T A T G C G C T A T G A C A G C T T A G C G C T A | PBX1(Homeobox)/MCF7-PBX1-ChIP-Seq(GSE28007)/Homer | 1e-2 | -4.971e+00 | 0.0288 | 47.0 | 1.00% | 288.5 | 0.68% | motif file (matrix) | svg |
| 107 | T C A G A T C G G A C T A C T G G A C T C A G T C T A G C G T A G T A C C G T A C T A G A T C G | Tbx20(T-box)/Heart-Tbx20-ChIP-Seq(GSE29636)/Homer | 1e-2 | -4.817e+00 | 0.0333 | 138.0 | 2.95% | 1009.3 | 2.39% | motif file (matrix) | svg |
| 108 | C T A G T C A G C A G T T C A G A C T G A C T G G A T C C T A G A C T G C T A G T C A G A T G C | KLF14(Zf)/HEK293-KLF14.GFP-ChIP-Seq(GSE58341)/Homer | 1e-2 | -4.810e+00 | 0.0333 | 1910.0 | 40.79% | 16529.2 | 39.07% | motif file (matrix) | svg |
| 109 | C T G A C T A G A C T G G C A T C T A G G C A T A T C G C T G A C G T A G T C A | Tbx21(T-box)/GM12878-TBX21-ChIP-Seq(Encode)/Homer | 1e-2 | -4.676e+00 | 0.0376 | 508.0 | 10.85% | 4148.4 | 9.80% | motif file (matrix) | svg |
| 110 | T G C A C T G A C A T G C T A G C A G T A G T C C G T A A T G C A T G C T A C G G C A T T C A G G T C A G A T C G T A C | ERE(NR),IR3/MCF7-ERa-ChIP-Seq(Unpublished)/Homer | 1e-2 | -4.625e+00 | 0.0392 | 189.0 | 4.04% | 1436.7 | 3.40% | motif file (matrix) | svg |
