# Supplementary material for: Detailed molecular and epigenetic characterization of the pig IPEC-J2 and chicken SL-29 cell lines
Source: iScience. 2023 Feb 20;26(3):106252. doi: 10.1016/j.isci.2023.106252 (PMC10018572; doi:10.1016/j.isci.2023.106252)
Supplement: Data S2. Complete homer output for identified motifs in Chicken SL-29, related to Tables 5 and 6 — Homer motif analysis results for histone modifications H3K4me1, H3K4me3, H3K27ac, enhancers, and ATAC-seq of chicken SL-29 cell line. Parameters for possible false positives is as mentioned earlier for S5. [file mmc3.zip › Data_S2/S6/Chicken_SL_29/motif_analyis_enhancer_regions/homerResults.html]

motif\_analyis\_enhancer\_regions/ - Homer de novo Motif Results


# Homer *de novo* Motif Results (motif\_analyis\_enhancer\_regions/)

Known Motif Enrichment Results  
Gene Ontology Enrichment Results  
If Homer is having trouble matching a motif to a known motif, try copy/pasting the matrix file into
STAMP  
More information on motif finding results: HOMER
| Description of Results
| Tips
  
Total target sequences = 2915  
Total background sequences = 44116  
\* - possible false positive  

|  |  |  |  |  |  |  |  |  |
| --- | --- | --- | --- | --- | --- | --- | --- | --- |
| Rank | Motif | P-value | log P-pvalue | % of Targets | % of Background | STD(Bg STD) | Best Match/Details | Motif File |
| 1 | C A T G G T A C G T A C A C T G A T G C A G T C C G T A A C G T | 1e-55 | -1.276e+02 | 28.06% | 16.41% | 91.0bp (112.7bp) | YY2/MA0748.2/Jaspar(0.974) More Information | Similar Motifs Found | motif file (matrix) |
| 2 | A T C G G A C T T C G A C T A G C T A G A T G C A G T C A G C T | 1e-47 | -1.087e+02 | 65.52% | 52.23% | 81.6bp (115.1bp) | ZNF711(Zf)/SHSY5Y-ZNF711-ChIP-Seq(GSE20673)/Homer(0.947) More Information | Similar Motifs Found | motif file (matrix) |
| 3 | T G C A T A G C G C A T G C T A T G C A T G A C T A G C T C A G | 1e-42 | -9.805e+01 | 54.92% | 42.23% | 85.7bp (115.5bp) | BMYB(HTH)/Hela-BMYB-ChIP-Seq(GSE27030)/Homer(0.750) More Information | Similar Motifs Found | motif file (matrix) |
| 4 | A C T G T C G A A G C T G T A C A T G C A C T G A T C G G A C T | 1e-33 | -7.779e+01 | 71.42% | 60.60% | 85.4bp (119.4bp) | Elk4(ETS)/Hela-Elk4-ChIP-Seq(GSE31477)/Homer(0.689) More Information | Similar Motifs Found | motif file (matrix) |
| 5 | G A C T A C T G T A C G A C G T T C A G T C G A A T C G A C G T T A C G A T G C A T C G A C T G | 1e-17 | -4.084e+01 | 24.70% | 18.22% | 82.6bp (116.3bp) | Klf9(Zf)/GBM-Klf9-ChIP-Seq(GSE62211)/Homer(0.657) More Information | Similar Motifs Found | motif file (matrix) |
| 6 | G C T A C T A G T G A C A G T C A T C G A C T G | 1e-16 | -3.837e+01 | 70.22% | 62.80% | 87.8bp (123.2bp) | POL010.1\_DCE\_S\_III/Jaspar(0.742) More Information | Similar Motifs Found | motif file (matrix) |
| 7 | G T A C A T G C G A C T T A C G C G T A C T A G C T A G A G T C | 1e-16 | -3.707e+01 | 15.88% | 10.83% | 84.5bp (105.1bp) | TFAP2A/MA0003.4/Jaspar(0.903) More Information | Similar Motifs Found | motif file (matrix) |
| 8 | T A G C G T A C G C T A A G T C A C G T C G A T A G T C T A G C A C T G A T C G | 1e-14 | -3.398e+01 | 12.62% | 8.31% | 97.7bp (114.2bp) | ZBTB7A/MA0750.2/Jaspar(0.966) More Information | Similar Motifs Found | motif file (matrix) |
| 9 | A C G T A T G C C T A G C G T A G C T A G T A C C A G T A T G C A G T C G T A C | 1e-12 | -2.890e+01 | 1.27% | 0.29% | 83.7bp (99.3bp) | ZNF135/MA1587.1/Jaspar(0.768) More Information | Similar Motifs Found | motif file (matrix) |
| 10 \* | T C A G T C G A T C G A T C A G A T C G A C G T T C G A T A G C A G T C A T C G T A C G T A C G | 1e-11 | -2.678e+01 | 6.59% | 3.87% | 79.9bp (110.7bp) | POL008.1\_DCE\_S\_I/Jaspar(0.564) More Information | Similar Motifs Found | motif file (matrix) |
| 11 \* | A T G C A C T G A C G T C T A G C G T A A C T G C A G T T C G A A C T G A G T C A T C G A T C G | 1e-11 | -2.556e+01 | 1.30% | 0.34% | 67.1bp (119.1bp) | MSANTD3/MA1523.1/Jaspar(0.648) More Information | Similar Motifs Found | motif file (matrix) |
| 12 \* | A T C G A C G T A C G T A G C T A T C G A G C T A G C T A G C T A C G T T G C A | 1e-10 | -2.531e+01 | 4.43% | 2.32% | 87.4bp (110.0bp) | FOXE1/MA1487.1/Jaspar(0.747) More Information | Similar Motifs Found | motif file (matrix) |
| 13 \* | A T C G G C T A C G A T A T G C T C G A A T C G G A C T G A C T A G T C A T C G | 1e-10 | -2.522e+01 | 5.76% | 3.31% | 78.4bp (113.6bp) | POL001.1\_MTE/Jaspar(0.639) More Information | Similar Motifs Found | motif file (matrix) |
| 14 \* | T A G C T A C G A T C G T A G C A T C G A T C G A C G T C A G T A T C G A C G T T A C G A T C G | 1e-10 | -2.485e+01 | 10.91% | 7.46% | 84.1bp (117.1bp) | MYB(HTH)/ERMYB-Myb-ChIPSeq(GSE22095)/Homer(0.705) More Information | Similar Motifs Found | motif file (matrix) |
| 15 \* | G A T C T A G C A C T G T A C G A G C T G C T A A G C T T C A G A C T G A C T G C T A G C T G A A T G C | 1e-10 | -2.395e+01 | 0.93% | 0.19% | 81.6bp (92.6bp) | MZF1/MA0056.2/Jaspar(0.604) More Information | Similar Motifs Found | motif file (matrix) |
| 16 \* | T C G A T A C G T A C G A C G T T C A G T C G A A T C G T A C G A T G C A T G C A T C G T A G C A C T G A T C G A T C G | 1e-10 | -2.353e+01 | 2.64% | 1.15% | 77.0bp (117.8bp) | Zfx/MA0146.2/Jaspar(0.631) More Information | Similar Motifs Found | motif file (matrix) |
| 17 \* | A C T G T C A G T C G A A C T G A G T C A C T G C T A G A C T G A C G T A C T G A G T C G T A C A G T C | 1e-10 | -2.346e+01 | 1.13% | 0.28% | 81.7bp (102.9bp) | PB0133.1\_Hic1\_2/Jaspar(0.669) More Information | Similar Motifs Found | motif file (matrix) |
| 18 \* | C G A T C A T G T G C A A C G T G T A C A G T C G T A C C A T G | 1e-10 | -2.344e+01 | 9.85% | 6.67% | 83.0bp (111.7bp) | RHOXF1/MA0719.1/Jaspar(0.758) More Information | Similar Motifs Found | motif file (matrix) |
| 19 \* | A G T C A G T C A G T C C G T A A C G T G C T A G T C A A C G T G T C A A C T G A G T C A G T C A T C G A T G C A G T C | 1e-9 | -2.294e+01 | 0.21% | 0.00% | 100.5bp (0.0bp) | ZNF410/MA0752.1/Jaspar(0.650) More Information | Similar Motifs Found | motif file (matrix) |
| 20 \* | C T G A A G T C A C T G C G T A A G C T T A G C | 1e-9 | -2.287e+01 | 42.71% | 36.97% | 86.6bp (113.8bp) | HOXA9/MA0594.2/Jaspar(0.654) More Information | Similar Motifs Found | motif file (matrix) |
| 21 \* | A G C T C A G T T A G C A C G T C G A T A C T G C T G A G C T A | 1e-9 | -2.284e+01 | 4.01% | 2.11% | 82.9bp (94.3bp) | Stat5a/MA1624.1/Jaspar(0.752) More Information | Similar Motifs Found | motif file (matrix) |
| 22 \* | C A T G A T C G G T A C A T G C T A C G A T C G C A T G A T G C G A C T A T C G | 1e-9 | -2.260e+01 | 35.75% | 30.29% | 84.2bp (117.7bp) | POL002.1\_INR/Jaspar(0.647) More Information | Similar Motifs Found | motif file (matrix) |
| 23 \* | T A C G G C T A C T A G T C A G A T C G A T G C A C G T A C G T A G T C A T C G A C G T A T C G | 1e-9 | -2.249e+01 | 0.51% | 0.06% | 73.2bp (75.6bp) | Npas4(bHLH)/Neuron-Npas4-ChIP-Seq(GSE127793)/Homer(0.628) More Information | Similar Motifs Found | motif file (matrix) |
| 24 \* | A G T C C A G T A C G T T A G C A G C T A C G T T G C A G T A C A G C T G C A T | 1e-9 | -2.224e+01 | 2.44% | 1.05% | 83.6bp (98.8bp) | POL008.1\_DCE\_S\_I/Jaspar(0.666) More Information | Similar Motifs Found | motif file (matrix) |
| 25 \* | A C T G A T C G A T G C C A G T A C T G T C G A T C G A T A C G A C T G T A G C T G A C C T A G T A G C A C T G A T C G | 1e-9 | -2.220e+01 | 2.50% | 1.09% | 82.6bp (121.6bp) | Zfx/MA0146.2/Jaspar(0.604) More Information | Similar Motifs Found | motif file (matrix) |
| 26 \* | C A G T G C T A T A G C C G T A A C G T G A T C C A G T A C G T | 1e-9 | -2.206e+01 | 3.16% | 1.53% | 88.4bp (92.3bp) | POL005.1\_DPE/Jaspar(0.671) More Information | Similar Motifs Found | motif file (matrix) |
| 27 \* | A C G T A G C T G A C T A C G T G A T C A C G T T A G C A C G T A G T C G A C T | 1e-9 | -2.104e+01 | 4.25% | 2.36% | 90.0bp (118.4bp) | PRDM1/MA0508.3/Jaspar(0.774) More Information | Similar Motifs Found | motif file (matrix) |
| 28 \* | C T G A C T G A A G T C G T C A A G T C C G A T A G T C G T C A A C T G G T A C A C G T A C T G A G T C | 1e-8 | -1.986e+01 | 0.34% | 0.02% | 83.9bp (62.8bp) | NHLH1/MA0048.2/Jaspar(0.730) More Information | Similar Motifs Found | motif file (matrix) |
| 29 \* | A G C T G A T C G A C T G A T C T C G A A C T G T A G C T A C G T C G A T A C G | 1e-8 | -1.958e+01 | 7.75% | 5.19% | 84.6bp (107.0bp) | Zic(Zf)/Cerebellum-ZIC1.2-ChIP-Seq(GSE60731)/Homer(0.750) More Information | Similar Motifs Found | motif file (matrix) |
| 30 \* | A C T G A C T G G T C A C G T A A C T G A T G C G T A C A G T C A G T C T C G A A C T G A C T G C G T A | 1e-8 | -1.843e+01 | 0.17% | 0.00% | 60.8bp (47.4bp) | REL/MA0101.1/Jaspar(0.673) More Information | Similar Motifs Found | motif file (matrix) |
| 31 \* | A G T C C G T A A C T G A C G T C G T A A G T C A G T C C G T A A G T C A G T C C G T A A C T G A C G T C G T A A G T C | 1e-8 | -1.842e+01 | 85.97% | 82.08% | 82.2bp (101.8bp) | PB0196.1\_Zbtb7b\_2/Jaspar(0.628) More Information | Similar Motifs Found | motif file (matrix) |
| 32 \* | C G T A G C T A G T A C T A C G C A T G C G A T G T C A A T G C | 1e-7 | -1.783e+01 | 7.31% | 4.94% | 90.2bp (113.9bp) | OVOL2/MA1545.1/Jaspar(0.795) More Information | Similar Motifs Found | motif file (matrix) |
| 33 \* | A T C G A C T G A C G T A G C T C T A G A G C T A C G T A C G T C G T A A C G T A C T G A C T G A C T G A C T G A T G C | 1e-7 | -1.755e+01 | 55.99% | 50.92% | 82.9bp (96.7bp) | HOXB13(Homeobox)/ProstateTumor-HOXB13-ChIP-Seq(GSE56288)/Homer(0.647) More Information | Similar Motifs Found | motif file (matrix) |
| 34 \* | A G T C A G C T A T C G A T C G T C A G A T G C G T A C A C G T T A C G C A T G T A C G A T G C A T G C G A C T C T A G | 1e-7 | -1.711e+01 | 1.48% | 0.57% | 72.1bp (122.2bp) | Zfx/MA0146.2/Jaspar(0.782) More Information | Similar Motifs Found | motif file (matrix) |
| 35 \* | C G T A C G T A A C G T A G T C A C T G A G T C A C T G G T A C A C G T A C T G | 1e-6 | -1.535e+01 | 0.24% | 0.02% | 57.1bp (87.1bp) | PB0185.1\_Tcf1\_2/Jaspar(0.579) More Information | Similar Motifs Found | motif file (matrix) |
| 36 \* | G C T A A T G C T C G A G C T A T A G C A T C G C T G A G T A C | 1e-6 | -1.500e+01 | 7.55% | 5.34% | 81.2bp (103.7bp) | KLF9/MA1107.2/Jaspar(0.598) More Information | Similar Motifs Found | motif file (matrix) |
| 37 \* | A C G T C G A T A T G C C G A T C T G A G T A C A C G T C G T A A C T G A C G T G A T C A C T G C G T A | 1e-5 | -1.351e+01 | 0.21% | 0.01% | 59.9bp (57.1bp) | VENTX/MA0724.1/Jaspar(0.602) More Information | Similar Motifs Found | motif file (matrix) |
| 38 \* | A G T C A G C T T G C A A C G T A C T G A G T C A G T C T C A G A C G T A C T G A G T C A G C T G C T A | 1e-5 | -1.351e+01 | 0.21% | 0.01% | 87.6bp (55.2bp) | PB0029.1\_Hic1\_1/Jaspar(0.575) More Information | Similar Motifs Found | motif file (matrix) |
| 39 \* | A G T C A G T C A C T G A C G T A T G C C G T A C T A G A G T C G A C T A C T G A G T C C G T A A T C G A G T C C G T A | 1e-5 | -1.326e+01 | 0.31% | 0.04% | 95.5bp (53.5bp) | PRDM9(Zf)/Testis-DMC1-ChIP-Seq(GSE35498)/Homer(0.576) More Information | Similar Motifs Found | motif file (matrix) |
| 40 \* | A G T C A C T G A C T G C A T G T A G C A G T C A C T G A T C G C T A G T G A C A G T C A C T G A T C G | 1e-5 | -1.280e+01 | 15.75% | 12.84% | 86.6bp (123.3bp) | KLF15/MA1513.1/Jaspar(0.601) More Information | Similar Motifs Found | motif file (matrix) |
| 41 \* | A T G C A T C G C G A T A C T G A G C T G C A T C T A G C G A T C A T G G T A C T A G C A G T C A T G C | 1e-5 | -1.279e+01 | 20.27% | 17.03% | 84.8bp (105.6bp) | PB0120.1\_Foxj1\_2/Jaspar(0.717) More Information | Similar Motifs Found | motif file (matrix) |
| 42 \* | A T G C A G T C A G T C T A C G T G A C G T C A T A G C C G T A A T C G T A G C T G A C C T A G A T G C | 1e-5 | -1.217e+01 | 9.02% | 6.85% | 84.7bp (117.5bp) | PB0151.1\_Myf6\_2/Jaspar(0.611) More Information | Similar Motifs Found | motif file (matrix) |
| 43 \* | A G T C C G T A C G T A A C T G A G T C A C G T A C G T A G T C C G T A A C T G A G T C A C G T A C T G | 1e-5 | -1.160e+01 | 0.21% | 0.02% | 88.1bp (44.1bp) | Nr2e3/MA0164.1/Jaspar(0.712) More Information | Similar Motifs Found | motif file (matrix) |
| 44 \* | T A G C T A G C T A C G T A G C C A T G G T A C C G T A C A T G G T A C T G C A C T A G T A C G T A G C T A C G T A C G | 1e-4 | -1.046e+01 | 13.83% | 11.38% | 82.9bp (112.5bp) | Zic1::Zic2/MA1628.1/Jaspar(0.824) More Information | Similar Motifs Found | motif file (matrix) |
| 45 \* | A G T C C T A G A C G T A C G T T C G A A G T C A C T G A G T C A C G T A C T G G T A C A C G T A T G C | 1e-4 | -9.934e+00 | 0.86% | 0.34% | 90.7bp (119.9bp) | Spz1/MA0111.1/Jaspar(0.575) More Information | Similar Motifs Found | motif file (matrix) |
| 46 \* | T A C G A T C G T A C G A T G C A G C T A T G C G A C T A C G T A G T C A T C G | 1e-4 | -9.594e+00 | 3.91% | 2.68% | 72.3bp (114.2bp) | ZNF341(Zf)/EBV-ZNF341-ChIP-Seq(GSE113194)/Homer(0.778) More Information | Similar Motifs Found | motif file (matrix) |
| 47 \* | C A G T T G C A C T A G G A T C A G C T C G T A G C A T T C A G T C G A T C A G | 1e-3 | -9.141e+00 | 2.13% | 1.27% | 92.3bp (102.7bp) | Nkx2.5(Homeobox)/HL1-Nkx2.5.biotin-ChIP-Seq(GSE21529)/Homer(0.532) More Information | Similar Motifs Found | motif file (matrix) |
| 48 \* | A G T C C G T A A C T G A G T C A C G T A C T G A C T G A C T G C G T A A C G T C G T A A C G T A C T G A C T G A C T G | 1e-3 | -7.962e+00 | 0.10% | 0.00% | 59.7bp (47.2bp) | Tcf21(bHLH)/ArterySmoothMuscle-Tcf21-ChIP-Seq(GSE61369)/Homer(0.638) More Information | Similar Motifs Found | motif file (matrix) |
| 49 \* | C G T A C G A T C G T A C G A T C G T A G A C T C T G A A G C T C T G A A C G T C G T A C G A T C G T A C G A T C T G A | 1e-2 | -6.795e+00 | 0.10% | 0.01% | 21.8bp (12.3bp) | OCT:OCT(POU,Homeobox)/NPC-OCT6-ChIP-Seq(GSE43916)/Homer(0.783) More Information | Similar Motifs Found | motif file (matrix) |
| 50 \* | C A G T G T A C A C G T T G C A G A C T C G A T G C T A G A T C C T G A G T A C G T C A G T A C C A T G G C T A G T C A | 1e-2 | -6.183e+00 | 13.65% | 11.89% | 87.0bp (103.6bp) | TBX20/MA0689.1/Jaspar(0.509) More Information | Similar Motifs Found | motif file (matrix) |
| 51 \* | A T G C A T C G A C G T A G T C C T A G C G A T A G T C A C T G A C G T A G T C C T A G A C G T A G T C A C T G G A C T | 1e-1 | -3.416e+00 | 0.14% | 0.04% | 37.6bp (10.3bp) | PB0131.1\_Gmeb1\_2/Jaspar(0.644) More Information | Similar Motifs Found | motif file (matrix) |
