# Supplementary material for: Detailed molecular and epigenetic characterization of the pig IPEC-J2 and chicken SL-29 cell lines
Source: iScience. 2023 Feb 20;26(3):106252. doi: 10.1016/j.isci.2023.106252 (PMC10018572; doi:10.1016/j.isci.2023.106252)
Supplement: Data S2. Complete homer output for identified motifs in Chicken SL-29, related to Tables 5 and 6 — Homer motif analysis results for histone modifications H3K4me1, H3K4me3, H3K27ac, enhancers, and ATAC-seq of chicken SL-29 cell line. Parameters for possible false positives is as mentioned earlier for S5. [file mmc3.zip › Data_S2/S6/Chicken_SL_29/motif_analyis_enhancer_regions/homerResults/motif1.similar.html]

motif1

## Information for motif1

C
A
T
G
G
T
A
C
G
T
A
C
A
C
T
G
A
T
G
C
A
G
T
C
C
G
T
A
A
C
G
T
  
Reverse Opposite:  

C
G
T
A
A
C
G
T
C
T
A
G
A
T
C
G
G
T
A
C
A
C
T
G
C
A
T
G
G
T
A
C
  

|  |  |
| --- | --- |
| p-value: | 1e-55 |
| log p-value: | -1.276e+02 |
| Information Content per bp: | 1.875 |
| Number of Target Sequences with motif | 818.0 |
| Percentage of Target Sequences with motif | 28.06% |
| Number of Background Sequences with motif | 7237.7 |
| Percentage of Background Sequences with motif | 16.41% |
| Average Position of motif in Targets | 141.6 +/- 91.0bp |
| Average Position of motif in Background | 149.8 +/- 112.7bp |
| Strand Bias (log2 ratio + to - strand density) | -0.0 |
| Multiplicity (# of sites on avg that occur together) | 1.15 |
| Motif File: | file (matrix) reverse opposite |

### Similar de novo motifs found

|  |  |  |  |  |  |  |  |
| --- | --- | --- | --- | --- | --- | --- | --- |
| Rank | Match Score | Redundant Motif | P-value | log P-value | % of Targets | % of Background | Motif file |
| 1 | 0.899 | A G T C A C T G G T A C A G T C C G T A A C G T | 1e-42 | -98.603945 | 29.40% | 18.80% | motif file (matrix) |
| 2 | 0.838 | A C T G A G T C T G A C C A T G T A G C A G T C T C G A A G C T T C A G C A T G A G T C C A T G | 1e-38 | -89.594203 | 15.99% | 8.46% | motif file (matrix) |
| 3 | 0.899 | C T G A A T C G A T C G G A T C T G A C A C T G A T G C T G A C G T C A A G C T | 1e-36 | -84.456601 | 34.17% | 23.70% | motif file (matrix) |
| 4 | 0.867 | T A G C T C A G T A G C T A G C T C G A C A G T A T C G T A C G G T A C C T A G T A C G T A G C T A C G T A C G A T G C | 1e-32 | -75.338699 | 19.55% | 11.80% | motif file (matrix) |
| 5 | 0.731 | A G T C C T A G T G A C T G A C T C G A A C G T C T A G C G T A G A C T A T C G | 1e-29 | -67.542758 | 46.76% | 36.48% | motif file (matrix) |
| 6 | 0.647 | G T A C C G A T C T A G T A C G G T A C T A G C A C T G A T G C | 1e-26 | -61.382531 | 39.18% | 29.80% | motif file (matrix) |
| 7 | 0.928 | T A G C A C T G T A C G G C T A C G A T C T A G C A T G G A T C A T C G A T C G T G A C A T G C A T G C | 1e-25 | -58.447169 | 50.36% | 40.70% | motif file (matrix) |
| 8 | 0.792 | A T G C T A G C C T A G G T A C A T G C T G C A A G C T T A C G C T A G T G A C A C T G T A C G T A G C | 1e-25 | -58.149959 | 25.21% | 17.46% | motif file (matrix) |
| 9 | 0.621 | C T A G A T G C A T G C A T G C A T C G A T G C G C A T T C G A | 1e-22 | -52.114098 | 71.05% | 62.32% | motif file (matrix) |
| 10 | 0.810 | T A C G T A G C T C G A T A C G A C T G T G A C G A T C A C T G A T G C G T A C T G C A C A G T | 1e-19 | -45.107912 | 10.81% | 6.29% | motif file (matrix) |
| 11 | 0.816 | C G T A T G C A C T G A T C G A G A C T C T A G A C T G T G A C C T A G T A C G T G A C A T C G A T C G | 1e-19 | -43.902397 | 8.78% | 4.80% | motif file (matrix) |
| 12 | 0.720 | T C G A T C G A T C G A T C G A A G C T T A C G T A C G T G A C A T C G T A C G A G C T A T C G | 1e-16 | -39.106585 | 6.28% | 3.16% | motif file (matrix) |
| 13 | 0.739 | T A G C T A G C C T A G G T A C T A G C C G T A A C G T A T C G C A T G G A T C T A G C A T G C | 1e-12 | -29.333875 | 6.76% | 3.88% | motif file (matrix) |
| 14 | 0.752 | A G T C C T G A A T C G A G T C C T A G A G T C T A G C A C T G A G T C A G T C C G T A A C G T A G T C A C G T A C G T | 1e-12 | -28.592769 | 2.30% | 0.83% | motif file (matrix) |
| 15 | 0.790 | C G A T A T G C A G T C A C T G A G T C A G T C A T C G A T G C G A T C G T C A C A G T G T A C | 1e-11 | -27.356356 | 2.20% | 0.79% | motif file (matrix) |
| 16 | 0.616 | A G T C C T G A C G T A C A T G G T C A A G C T A C T G A C T G A G T C C T G A A C T G A T G C A C T G G C A T A G T C | 1e-8 | -19.023589 | 0.31% | 0.02% | motif file (matrix) |
| 17 | 0.601 | C T A G A C T G C G T A C T G A A C T G C T A G A G C T A T C G C T G A A G T C A C T G C T A G A G T C A C T G A C T G | 1e-7 | -16.700096 | 0.75% | 0.19% | motif file (matrix) |
| 18 | 0.714 | G T A C A C T G A T C G A G T C C G T A G A C T | 1e-5 | -12.106892 | 12.25% | 9.74% | motif file (matrix) |
| 19 | 0.874 | A C G T A C T G A C T G A G T C A C T G A C T G | 1e-3 | -7.948107 | 99.83% | 99.39% | motif file (matrix) |
