# Supplementary material for: Detailed molecular and epigenetic characterization of the pig IPEC-J2 and chicken SL-29 cell lines
Source: iScience. 2023 Feb 20;26(3):106252. doi: 10.1016/j.isci.2023.106252 (PMC10018572; doi:10.1016/j.isci.2023.106252)
Supplement: Data S2. Complete homer output for identified motifs in Chicken SL-29, related to Tables 5 and 6 — Homer motif analysis results for histone modifications H3K4me1, H3K4me3, H3K27ac, enhancers, and ATAC-seq of chicken SL-29 cell line. Parameters for possible false positives is as mentioned earlier for S5. [file mmc3.zip › Data_S2/S6/Chicken_SL_29/motif_analyis_enhancer_regions/homerResults/motif13.similar.html]

motif13

## Information for motif13

A
T
C
G
G
C
T
A
C
G
A
T
A
T
G
C
T
C
G
A
A
T
C
G
G
A
C
T
G
A
C
T
A
G
T
C
A
T
C
G
  
Reverse Opposite:  

T
A
G
C
A
C
T
G
C
T
G
A
C
T
G
A
A
T
G
C
A
C
G
T
A
T
C
G
G
C
T
A
C
G
A
T
A
T
G
C
  

|  |  |
| --- | --- |
| p-value: | 1e-10 |
| log p-value: | -2.522e+01 |
| Information Content per bp: | 1.691 |
| Number of Target Sequences with motif | 168.0 |
| Percentage of Target Sequences with motif | 5.76% |
| Number of Background Sequences with motif | 1460.8 |
| Percentage of Background Sequences with motif | 3.31% |
| Average Position of motif in Targets | 134.2 +/- 78.4bp |
| Average Position of motif in Background | 145.0 +/- 113.6bp |
| Strand Bias (log2 ratio + to - strand density) | -0.1 |
| Multiplicity (# of sites on avg that occur together) | 1.08 |
| Motif File: | file (matrix) reverse opposite |

### Similar de novo motifs found

|  |  |  |  |  |  |  |  |
| --- | --- | --- | --- | --- | --- | --- | --- |
| Rank | Match Score | Redundant Motif | P-value | log P-value | % of Targets | % of Background | Motif file |
| 1 | 0.644 | A T G C A C T G C T A G C A G T C G A T A G T C A C G T C T A G A C G T A G C T A G T C A C T G A T C G A G C T G A C T | 1e-10 | -25.188876 | 62.40% | 56.28% | motif file (matrix) |
| 2 | 0.607 | T A G C A T C G C G A T C T G A A T G C G A C T | 1e-9 | -22.360280 | 35.27% | 29.86% | motif file (matrix) |
| 3 | 0.710 | C T G A A T G C T G A C C T A G G T C A T C G A G T A C A T C G T A C G C T G A C T G A T G A C | 1e-5 | -13.096234 | 0.96% | 0.34% | motif file (matrix) |
