# Supplementary material for: Detailed molecular and epigenetic characterization of the pig IPEC-J2 and chicken SL-29 cell lines
Source: iScience. 2023 Feb 20;26(3):106252. doi: 10.1016/j.isci.2023.106252 (PMC10018572; doi:10.1016/j.isci.2023.106252)
Supplement: Data S2. Complete homer output for identified motifs in Chicken SL-29, related to Tables 5 and 6 — Homer motif analysis results for histone modifications H3K4me1, H3K4me3, H3K27ac, enhancers, and ATAC-seq of chicken SL-29 cell line. Parameters for possible false positives is as mentioned earlier for S5. [file mmc3.zip › Data_S2/S6/Chicken_SL_29/motif_analyis_enhancer_regions/homerResults/motif2.similar.html]

motif2

## Information for motif2

A
T
C
G
G
A
C
T
T
C
G
A
C
T
A
G
C
T
A
G
A
T
G
C
A
G
T
C
A
G
C
T
  
Reverse Opposite:  

T
C
G
A
C
T
A
G
A
T
C
G
A
G
T
C
G
A
T
C
A
G
C
T
C
T
G
A
T
A
G
C
  

|  |  |
| --- | --- |
| p-value: | 1e-47 |
| log p-value: | -1.087e+02 |
| Information Content per bp: | 1.667 |
| Number of Target Sequences with motif | 1910.0 |
| Percentage of Target Sequences with motif | 65.52% |
| Number of Background Sequences with motif | 23038.8 |
| Percentage of Background Sequences with motif | 52.23% |
| Average Position of motif in Targets | 154.4 +/- 81.6bp |
| Average Position of motif in Background | 149.7 +/- 115.1bp |
| Strand Bias (log2 ratio + to - strand density) | 0.0 |
| Multiplicity (# of sites on avg that occur together) | 2.01 |
| Motif File: | file (matrix) reverse opposite |

### Similar de novo motifs found

|  |  |  |  |  |  |  |  |
| --- | --- | --- | --- | --- | --- | --- | --- |
| Rank | Match Score | Redundant Motif | P-value | log P-value | % of Targets | % of Background | Motif file |
| 1 | 0.940 | T A C G A C T G A C T G A G T C A G T C A C G T | 1e-46 | -107.050654 | 60.00% | 46.69% | motif file (matrix) |
| 2 | 0.904 | T C G A C A T G T A C G A G T C A T G C A G C T T A C G C A T G A T C G C T G A | 1e-45 | -105.063310 | 42.85% | 30.32% | motif file (matrix) |
| 3 | 0.783 | T G C A C T A G A T C G A T G C T G A C A C G T T A G C C T G A | 1e-38 | -89.281463 | 35.75% | 24.82% | motif file (matrix) |
| 4 | 0.629 | A C T G C A G T T C G A T G C A T C A G A T C G A T C G A T G C A T G C A C T G | 1e-36 | -83.527828 | 55.57% | 43.87% | motif file (matrix) |
| 5 | 0.804 | A G T C A C T G A C T G A C T G A G T C A G T C | 1e-32 | -75.670811 | 66.17% | 55.22% | motif file (matrix) |
| 6 | 0.680 | C T G A A T C G A T C G A T G C A T G C A C G T A T C G T A C G T A C G G A T C G A T C G C A T | 1e-26 | -61.605739 | 13.48% | 7.64% | motif file (matrix) |
| 7 | 0.674 | C A G T A T C G T A C G C T A G G T A C A G T C A G T C T A C G T A C G A C G T | 1e-25 | -59.082602 | 45.18% | 35.64% | motif file (matrix) |
| 8 | 0.858 | A C T G A C T G A G T C A G T C A G T C C G T A | 1e-24 | -56.856306 | 51.29% | 41.74% | motif file (matrix) |
| 9 | 0.697 | C A G T T A C G G T C A A C T G A T C G A C T G T A G C A T G C | 1e-19 | -44.270591 | 30.09% | 22.79% | motif file (matrix) |
| 10 | 0.634 | G T A C A T C G T A G C A C T G T A C G T A G C T A G C G A C T A T C G T C G A T A G C T A G C | 1e-18 | -42.709120 | 16.50% | 11.00% | motif file (matrix) |
| 11 | 0.719 | T C G A T A C G A C T G T G A C G T A C G C A T G A T C A G T C | 1e-17 | -39.640084 | 50.87% | 42.98% | motif file (matrix) |
| 12 | 0.618 | T C G A T A C G T A C G T A G C A G T C A T G C T A C G T A C G A T C G T A G C A G T C A C G T A T C G | 1e-16 | -39.019703 | 58.25% | 50.40% | motif file (matrix) |
| 13 | 0.722 | C A G T A T G C C G A T T A C G T G C A A C T G C T A G A G T C A G T C A T C G | 1e-16 | -38.436005 | 6.59% | 3.40% | motif file (matrix) |
| 14 | 0.608 | A C G T T A C G T C G A T C A G A C T G T C A G A T G C A G T C A T C G A T C G T C A G A T G C | 1e-16 | -38.192092 | 26.55% | 20.09% | motif file (matrix) |
| 15 | 0.606 | T G A C G T A C T C A G T A C G A T G C T G A C A T G C T C G A T C G A T G A C T C G A A T G C | 1e-15 | -35.626508 | 9.91% | 6.03% | motif file (matrix) |
| 16 | 0.657 | A T G C C A T G A T C G G A T C G T A C G C A T C G T A T A C G A G T C T C A G | 1e-14 | -34.435027 | 7.31% | 4.08% | motif file (matrix) |
| 17 | 0.673 | T G C A T C A G A T C G T A G C A T G C A G C T A T G C A T C G A T G C C A G T C T A G C A T G | 1e-14 | -34.151783 | 8.92% | 5.33% | motif file (matrix) |
| 18 | 0.667 | A T G C C A G T A C T G A C G T T C G A A T C G C A T G A T G C A G T C A T C G G T A C A C T G A T C G | 1e-13 | -31.468953 | 8.89% | 5.43% | motif file (matrix) |
| 19 | 0.766 | A G T C G A T C A G T C A C T G A T C G T C A G A C T G T A G C G A T C A G C T G A C T T G A C | 1e-13 | -31.181625 | 11.32% | 7.41% | motif file (matrix) |
| 20 | 0.664 | G C T A T A C G T A C G T C A G T A C G A G T C G T A C A T G C C A T G T A G C | 1e-13 | -30.281559 | 9.26% | 5.79% | motif file (matrix) |
| 21 | 0.616 | A G T C A G T C A C T G A C T G A C T G A G T C | 1e-13 | -30.067127 | 99.93% | 98.75% | motif file (matrix) |
| 22 | 0.620 | T A C G C G A T T A C G T A C G A C T G T A C G A T G C A T G C A G T C A C T G A T G C C A G T | 1e-12 | -29.918632 | 11.29% | 7.45% | motif file (matrix) |
| 23 | 0.638 | A C T G G T A C C A T G A C T G T A C G A T G C A G T C A G C T A C T G T C A G A T C G T C A G | 1e-12 | -28.394186 | 3.74% | 1.74% | motif file (matrix) |
| 24 | 0.630 | C T G A C T A G A T C G A G T C A G T C A C T G A G T C C T A G A T C G A T G C A T G C A G T C T A C G | 1e-12 | -28.308846 | 7.89% | 4.81% | motif file (matrix) |
| 25 | 0.666 | A T C G G T C A A C T G A C T G A G T C A G T C A G C T G T C A A T G C A G T C A T G C A T G C A C T G C T A G T G A C | 1e-8 | -18.977557 | 0.75% | 0.16% | motif file (matrix) |
