# Supplementary material for: Detailed molecular and epigenetic characterization of the pig IPEC-J2 and chicken SL-29 cell lines
Source: iScience. 2023 Feb 20;26(3):106252. doi: 10.1016/j.isci.2023.106252 (PMC10018572; doi:10.1016/j.isci.2023.106252)
Supplement: Data S2. Complete homer output for identified motifs in Chicken SL-29, related to Tables 5 and 6 — Homer motif analysis results for histone modifications H3K4me1, H3K4me3, H3K27ac, enhancers, and ATAC-seq of chicken SL-29 cell line. Parameters for possible false positives is as mentioned earlier for S5. [file mmc3.zip › Data_S2/S6/Chicken_SL_29/motif_analyis_enhancer_regions/homerResults/motif3.similar.html]

motif3

## Information for motif3

T
G
C
A
T
A
G
C
G
C
A
T
G
C
T
A
T
G
C
A
T
G
A
C
T
A
G
C
T
C
A
G
  
Reverse Opposite:  

A
G
T
C
A
T
C
G
A
C
T
G
A
C
G
T
C
G
A
T
C
G
T
A
A
T
C
G
A
C
G
T
  

|  |  |
| --- | --- |
| p-value: | 1e-42 |
| log p-value: | -9.805e+01 |
| Information Content per bp: | 1.446 |
| Number of Target Sequences with motif | 1601.0 |
| Percentage of Target Sequences with motif | 54.92% |
| Number of Background Sequences with motif | 18627.6 |
| Percentage of Background Sequences with motif | 42.23% |
| Average Position of motif in Targets | 151.2 +/- 85.7bp |
| Average Position of motif in Background | 150.5 +/- 115.5bp |
| Strand Bias (log2 ratio + to - strand density) | -0.2 |
| Multiplicity (# of sites on avg that occur together) | 1.44 |
| Motif File: | file (matrix) reverse opposite |

### Similar de novo motifs found

|  |  |  |  |  |  |  |  |
| --- | --- | --- | --- | --- | --- | --- | --- |
| Rank | Match Score | Redundant Motif | P-value | log P-value | % of Targets | % of Background | Motif file |
| 1 | 0.638 | A T C G A C T G A C G T C T A G C T G A A C T G C G A T T C A G T C A G A T G C | 1e-30 | -70.518784 | 21.85% | 13.90% | motif file (matrix) |
| 2 | 0.708 | C G A T G C T A T G A C A T G C C A T G C A T G | 1e-30 | -69.549042 | 77.32% | 67.65% | motif file (matrix) |
| 3 | 0.646 | A G T C T C A G A G T C C A G T G T C A G T C A T A G C T G C A | 1e-26 | -61.946704 | 26.62% | 18.44% | motif file (matrix) |
| 4 | 0.654 | A C T G A C G T C T A G C G T A A C T G A C G T | 1e-21 | -49.706678 | 17.26% | 11.22% | motif file (matrix) |
| 5 | 0.629 | T A G C C T A G A C G T G C T A C G T A C A T G | 1e-17 | -40.072649 | 34.61% | 27.33% | motif file (matrix) |
| 6 | 0.842 | G C A T T G C A C T G A T G A C G T A C A C T G | 1e-15 | -36.788439 | 72.21% | 65.07% | motif file (matrix) |
| 7 | 0.617 | A T C G T A G C T A C G A T C G A T G C T A G C T A C G A C T G C G A T T G C A T C G A A T C G | 1e-15 | -35.642412 | 51.70% | 44.22% | motif file (matrix) |
| 8 | 0.606 | A T G C G T A C G A C T A T C G G T C A A G C T T A G C T C A G | 1e-8 | -20.127755 | 5.39% | 3.26% | motif file (matrix) |
